# The biomechanical role of the chondrocranium and sutures in a lizard cranium

**DOI:** 10.1098/rsif.2017.0637

**Published:** 2017-12-20

**Authors:** Marc E. H. Jones, Flora Gröning, Hugo Dutel, Alana Sharp, Michael J. Fagan, Susan E. Evans

**Affiliations:** 1 School of Biological Sciences, The University of Adelaide, North Terrace, Adelaide, South Australia 5005, Australia; 2 South Australian Museum, North Terrace, Adelaide, South Australia 5001, Australia; 3 School of Medicine, Medical Sciences and Nutrition, University of Aberdeen, Aberdeen AB25 2ZD, UK; 4 School of Engineering and Computer Science, Medical and Biological Engineering Research Group, University of Hull, Hull HU6 7RX, UK; 5 Research Department of Cell and Developmental Biology, UCL, University College London, Anatomy Building, Gower Street, London WCIE 6BT, UK

**Keywords:** chondrocranium, finite element analysis, skull, sutures, septum, cartilage

## Abstract

The role of soft tissues in skull biomechanics remains poorly understood. Not least, the chondrocranium, the portion of the braincase which persists as cartilage with varying degrees of mineralization. It also remains commonplace to overlook the biomechanical role of sutures despite evidence that they alter strain distribution. Here, we examine the role of both the sutures and the chondrocranium in the South American tegu lizard *Salvator merianae*. We use multi-body dynamics analysis (MDA) to provide realistic loading conditions for anterior and posterior unilateral biting and a detailed finite element model to examine strain magnitude and distribution. We find that strains within the chondrocranium are greatest during anterior biting and are primarily tensile; also that strain within the cranium is not greatly reduced by the presence of the chondrocranium unless it is given the same material properties as bone. This result contradicts previous suggestions that the anterior portion (the nasal septum) acts as a supporting structure. Inclusion of sutures to the cranium model not only increases overall strain magnitudes but also leads to a more complex distribution of tension and compression rather than that of a beam under sagittal bending.

## 1. Introduction

Lizards and tuatara are ideal taxa for investigating the evolution of skull mechanics as they exhibit a wide range of skull shapes, muscle arrangements, feeding behaviours and life styles (e.g. [1–3]). Accurate computer models of the skull generated using X-ray computed tomography and quantitative analytical approaches such as multi-body dynamics (MDA) and finite element analysis (FEA) provide powerful tools for testing specific hypotheses [4–8]. However, the role of soft-tissue structures such as the cartilaginous chondrocranium and fibrocellular cranial sutures, which are known to vary dramatically in structure among lepidosaurs (e.g. [2,9]), remains poorly known.

The chondrocranium is the portion of the braincase represented by cartilage [2,9–11]. In the majority of vertebrate taxa, the chondrocranium is largely replaced by bone during skull development but in tetrapods this replacement occurs relatively late [12]. The chondrocranium is derived from six components: the parachordals (which provide the posterior base of the braincase); the occipital and preoccipital arches (which support the posterior part of the brain); the otic capsules (which house the inner ear); the orbital cartilages (which sit medial to the eyes); the nasal capsules (which house the nasal apparatus) and a pair of rod-like trabeculae cranii that extend between the parachordals and nasal capsules beneath the orbital cartilage and interorbital septum [9,13]. In lepidosaurs, a significant part of the anteriormost portion of the chondrocranium is retained [9,13], e.g. the interorbital septum and framework of slender bars (derived from the orbital cartilage and posterior ends of the trabeculae cranii), the nasal septum (derived from the anterior ends of the trabeculae cranii) and the nasal capsules. By contrast, in mammals, the nasal cartilage is one of few components to persist into adulthood [14] but it appears to play a major role in the development of the snout [15–17].

Variation in the structure, histology and mineralization of the chondrocranium among vertebrates may be related to the strain it is subject to in life [18]. Its material properties are anisotropic due to the amounts and arrangements of constituent collagen and proteoglycans [18–20]. Within lepidosaurs, the chondrocranium also shows dramatic variation in structure and mineralization [9,10,13,21–24]. In many lizard taxa (including *Salvator*), the pila metoptica of the orbital cartilage ossifies to form an orbitosphenoid bone, whereas this element is absent in *Sphenodon*, gekkotans, dibamids and some anguimorphs [2,9]. Also, the pila antotica may ossify as a pleurosphenoid (in some gekkotans), the trabeculae cranii may mineralize as a septosphenoid (some gekkotans), other portions of the planum supraseptale ossify ventrally from the frontal bones (e.g. gekkotans, varanids), and a dermal parasphenoid rostrum supporting the interorbital septum may be present or absent (e.g. scincoids, most gekkotans) [2,9].

Given that the largely vertical structure of the chondrocranium lies along the mid-sagittal plane between the rostrum and ossified posterior braincase, one might expect it to affect strain distribution in adjacent bones. In humans and other mammals, the nasal septum has been inferred to represent an important vertical support because its absence (due to experimental removal, trauma or congenital circumstances) results in hypoplasia and/or partial displacement of the rostral elements [15,25,26]. *In vivo* strain measurements of the nasal septum in pigs reveal that during feeding it is subject to anteroposterior compression rather than dorsoventral compression. Therefore, a role related to absorbing dynamic strains that arise from feeding was suggested instead [27] and biomechanical investigations seem to support this possibility [27,28]. An examination of the nasal septum in crocodiles [20] found it to be formed of hyaline cartilage, with elastic fibres in the enclosing perichondrium. However, the authors identified an additional cord of collagen and elastic fibres running below the septum in the midline and suggested that this ‘tension cord’ might serve to resist tensile strains and stabilize the elongate rostrum during long-axis bending of the cranium. Investigation of the mechanical role of the chondrocranium in lepidosaurs is largely unexplored. Cleared and stained specimens of *Anolis* have been used to identify which parts of the chondrocranium buckle during mesokinetic flexion in the skull roof at the fronto-parietal joint [29]. A chondrocranium seems to have been included in the finite element model of an *Iguana* cranium ([30], figure 5) but its role was not reported or discussed.

The cranial sutures are fibrocellular joints between the bones of the skull [31,32]. Although often viewed as sites of bone growth [33] they are certainly not the only source of growth [34,35]. In the frame-like skulls of lepidosaurs, cranial sutures tend to involve large overlaps that may become more extensive with age [2,32]. There is also variation in their detailed histology, such as the arrangement of fibres and the contribution from cartilage [36,37]. In some lepidosaurs, several cranial sutures allow significant flexion between bones, movement often referred to as cranial kinesis, that may facilitate improved prey capture, prey handling, or bite force, or they may provide shock absorption (e.g. [1,38–40]). Sutures are known to have significantly different material properties to the surrounding bone [31,41] and *in vivo* work on mammals (e.g. [42,43]) and fish [44] suggests that patent sutures affect strain within the skull. Nevertheless, several studies analysing lepidosaur skulls with FEA have not included or greatly discussed sutures [45–47]. Although sutures are often considered to reduce strain (e.g. [48]), models that do incorporate sutures indicate that strain may actually be higher but more evenly distributed [49–51]. Moreover, how the strain is distributed is likely to be connected to the three-dimensional shape of the sutures [32]. Wider comparisons are clearly necessary.

Here, we investigate the role of both the chondrocranium and the sutures in the South American tegu lizard, *Salvator merianae* using load cases generated by a biomechanical model with wrapped muscles and minimal constraints (previously validated and published in [52]) in addition to a finite element model comprising both bone and soft tissue.

## 2. Material and methods

The specimen material comprised two adult female specimens (T1 and T3) of the South American tegu lizard, *S. merianae* (=*Tupinambis merianae*) [53]. This taxon and closely related species (such as *Dracaena guianensis*) are relatively well known in terms of their specific differences and phylogenetic relationships [53,54], dentition [55,56], diet [57], skull shape [56,58], fossil record [59,60], prey transport behaviour [61,62], physiology [63] and jaw muscles [52,64]. Tegus are known to eat plant material as well as taking a wide variety of prey items including both vertebrates and invertebrates (e.g. [57]). They have a relatively heterodont dentition [55,59] and their deep jaws are powerful [52,65].

Specimen T1's cranial length (as measured directly between the anteroventral point of the premaxilla and ventrolateral end of the left quadrate) is 92 mm and the width (as measured between the lateral surfaces of the base of the quadrates) is 56 mm. Specimen T3's cranial length and width are 105 mm and 62 mm, respectively. The dimensions of both specimens are within the range reported by Colli [57] for adult animals. The sutures and chondrocranium of *Salvator* have not previously been described.

**Figure 1.**
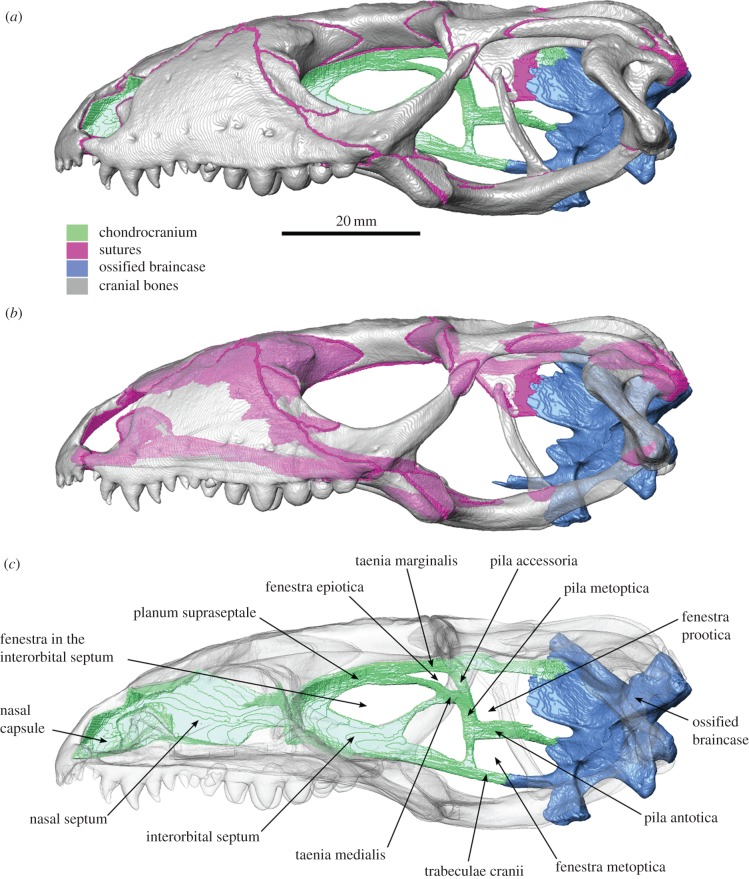
Fully segmented skull of *Salvator merianae* (=*Tupinambis merianae*), based on X-ray computed tomography, in lateral view with the cranial bones (*a*) opaque, (*b*) transparent to show the sutural overlaps and (*c*) transparent to show details of the chondrocranium. Scale bar, 20 mm.

An anatomically accurate three-dimensional computer model of the cranium, lower jaws and joint surfaces was built using a micro-computed X-ray tomography (μCT) dataset and the image segmentation software Avizo 6.3 (Visualization Sciences Group). Specimen T1 was scanned at the University of Hull, UK, using a X-Tek HMX 160 µCT system (X-Tek Systems Ltd, UK) and the following scan parameters: beryllium target, 113 kV, 25 µA; aperture 75%; 1000 projections. To reduce beam hardening the X-rays were filtered through a 0.1 copper plate. The final voxel size was 0.11 mm. The three-dimensional space occupied by the sutures was manually segmented out from the CT data so that every bone in the computer model was isolated from its neighbours by suture material (figure 1*a,b*; electronic supplementary material, figures S1–S8, cf. [51]). These sutures were typically 0.2 mm thick but reached approximately 0.5 mm thick in three places: between the premaxilla and maxilla; between the palatine and prefrontal; and between the quadrate, squamosal and parietal. Spaces within the bones were filled to facilitate meshing (electronic supplementary material, figure S9). We also generated a representation of the chondrocranium between the ossified braincase and nasal capsule (figure 1*c*). The majority of this structure has a thickness of between 0.5 and 1.5 mm (electronic supplementary material, figure S10). As in the lacertid lizard *Acanthodactylus* [13], the chondrocranium is made up of nasal capsules, a deep nasal septum and a deep interorbital septum, as well as continuous taenia marginalis, taenia medialis, pila accessoria, pila metopica and pila antotica.

Specimen T3 was cut parasagitally and the bones of the larger side were disarticulated using pig pancreatin (VWR International, CAS no.: 8049-47-6) to permit examination of the cranial suture structure under a binocular microscope. Twelve nanoindentation measurements were performed on the maxilla, which provided a Young's modulus of 17 032 ± 516 MPa (table 1).

**Table 1.** Material properties used in the FE model.

material	model	*E* (MPa)	*ν*	source of the data
bone	linear elastic	17 000	0.3	nano-indentation
teeth	linear elastic	17 000	0.3	see main text
suture	linear elastic	20	0.3	Kupczik *et al*. [41]
chondrocranium	linear elastic	20; 200; 17 000	0.3	see main text

A multi-body model of specimen T1 was constructed in the multi-body dynamics analysis (MDA) software ADAMS (MSC Software Corp.), using five rigid body models (most of the cranium, two quadrates and two lower jaws), as well as representation of the muscles (involving 116 strands) based on detailed dissections (as previously described in [52]; see also [7,66,67]). Movement was permitted between the quadrates and rest of the cranium (streptostyly) to be consistent with the degree of mobility found in the specimens prior to dissection of the jaw muscles [52].

MDA, using information from muscle physiological cross-sectional areas, was used to predict the loading from each muscle group and the resulting total bite force (electronic supplementary material, table S1). Bite force predictions from the models using a muscle intrinsic strength of 40 N cm^−2^ closely matched results from previous *in vivo* bite force experiments on the same individual animal [52] (electronic supplementary material, table S2).

For the FEA the model of the specimen T1 cranium was converted into a tetrahedral mesh consisting of 1.4 million solid (10 node) higher order elements. The sutures were necessarily enlarged during this process but remained less than 0.4 mm thick (electronic supplementary material, figure S11). The FEA was performed in ANSYS (Swanson Analysis Systems, Canonsburg, PA, USA). All the structures of the skull were modelled as linear elastic, isotropic materials, with a Poisson's ratio of 0.3. Bones and teeth were given Young's modulus values of 17 000 MPa using the nanoindentation results which compared well with values used elsewhere (cf. [46,51,68]). For ease of model segmentation and because we were not interested in the detailed strains in the teeth, we used the same property values for both teeth and bones. Variable bone properties have been used in finite element models before (e.g. [69,70]). However, whereas the variation in bone material properties can be relatively high (e.g. [71]), it is markedly less than the difference between bone and suture properties, and hence the effect of including sutures in the model will be significantly greater than the effect of including variable bone properties.

To investigate the effect of the chondrocranium on the strain distribution over the skull, we performed three FEA with Young's modulus values for the chondrocranium of 17 000 MPa (like bone), 200 MPa and 20 MPa (like cartilage). We are not aware of any published values for Young's modulus of the lepidosaur chondrocranium. Values for other types of vertebrate cartilage are available (e.g. [72–78]) but vary according to differences in histology, degree of mineralization, sample thickness, specimen preparation and the rate of loading used. Thus, a large range of values can be found in the literature for Young's modulus (0.5–564 MPa) of cartilage (e.g. [18,73,75,76,78–80]). Many values are derived from measurements under slow loading of unmineralized hyaline cartilage, but reported Young's modulus values are typically below 10 MPa (e.g. [78]). Values for the nasal septum of mammals tend to be less than 5 MPa [18,76,80]. By contrast, impact loading of unmineralized articular cartilage has yielded values of 50–200 MPa [77,81]. Our lower Young's modulus value of 20 MPa for the chondrocranium is closer to published values for impact loading, rather than slow loading, as we model fast forceful bites. Our higher value of 200 MPa lies at the upper end for impact loading of unmineralized articular cartilage and within the range for quasi-static loading of mineralized cartilage (20–564 MPa, [73]). We acknowledge that the properties of the chondrocranium are almost certainly not isotropic [18–20]. Moreover, we do not allocate the portion of chondrocranium which in *Salvator* forms an orbitosphenoid with the material properties of bone.

The cranial sutures were given a value of 20 MPa (cf. [41]). Material properties of the sutures are unknown for teiid lizards but they are likely to be more similar to those of sutures in other vertebrates than they are to those of bone.

FEA was used to predict the strain distribution in the cranium and the chondrocranium for both anterior biting (approx. 200 N, bite point 1 in [52]) and posterior biting (approx. 300 N, bite point 3 in [52]). Constraints were applied to the model to mimic the physiological boundary conditions experienced during biting. One node on each of the tips of the loaded teeth was constrained in all directions, and one node on the base of each quadrate constrained in a vertical direction. We employed these constraints because we aimed for a minimally constrained model to limit artefacts and we wanted the loading to include the horizontal components of bite force for a prey that was effectively immobile. Bite forces were thus modelled as reaction forces. To simulate the muscle forces, we imported the maximum muscle force magnitude and direction and coordinates of muscle origins for each muscle strand from our MDA model of the same specimen (T1). We examined 1st principal (most tensile) and 3rd principal (most compressive) strains as well as von Mises strain (a combination of all principal strains). Element solutions calculated in ANSYS were then exported and converted into .vtk files to be visualized in the software Paraview [82]. Tension–compression dominance contour plots were created by comparing the absolute values of the 1st (*ɛ*_1_) and 3rd (*ɛ*_3_) principal strain for each element of the mesh, and assigning an arbitrary value of 0 if *ɛ*_1_ > *ɛ*_3_, and 1 if *ɛ*_1_ < *ɛ*_3_ for each element. Hence, this contour plot gives information about the regions that are dominated by tensile or compressive strain without further information concerning their magnitude.

Quantitative estimates of the overall strain differences along the length of the models were obtained by dividing the cranium and chondrocranium into 10 sections and calculating a mean strain magnitude within each of those sections. Post-processing of the ANSYS results to generate .*vtk* files and quantitative data analyses were performed in the software R [83].

## 3. Results

### 3.1. Finite-element results: strains within the chondrocranium

Strain within the chondrocranium was much higher when sutures were included in the model of the cranium and slightly more regionalized but generally similar to the model without sutures. Alteration to the material properties of the chondrocranium made little difference to relative strain distribution, only magnitude.

**Figure 2.**
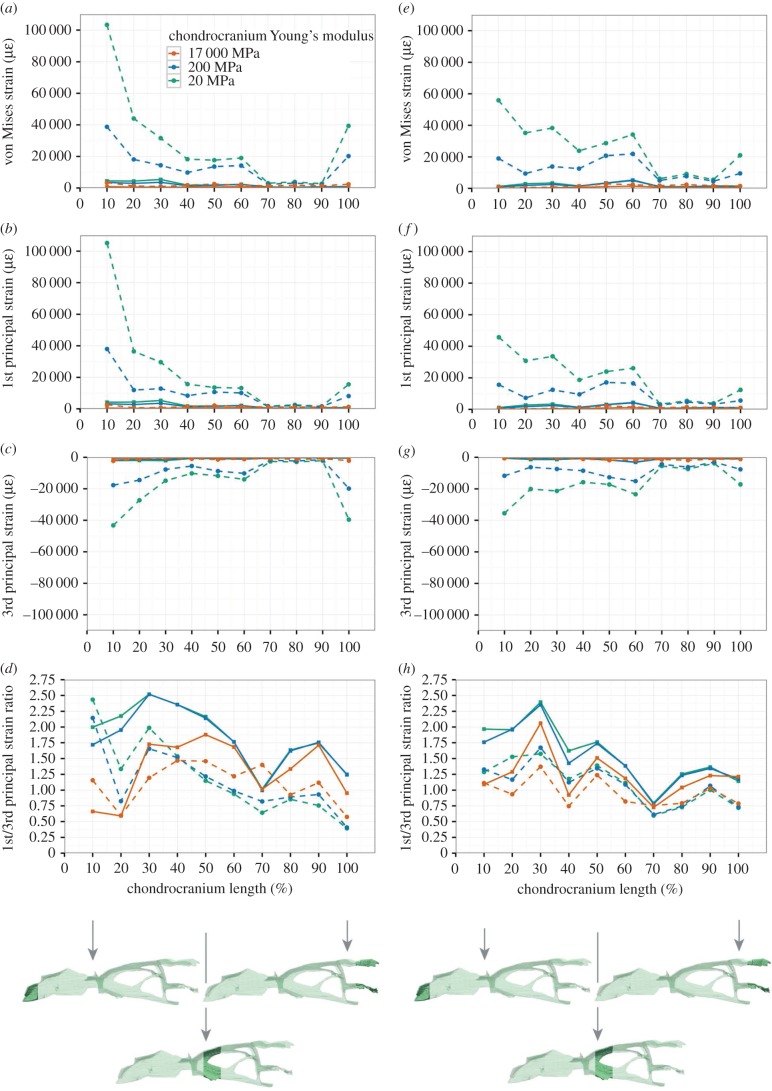
Strain plots within the chondrocranium during (*a–d*) anterior biting and (*e–h*) posterior unilateral biting. (*a,e*) von Mises strain, (*b,f*) 1st principal strain, (*c,g*) 3rd principal strain and (*d,h*) 1st/3rd principal strain ratio. Dashed lines represent models with patent sutures.

**Figure 3.**
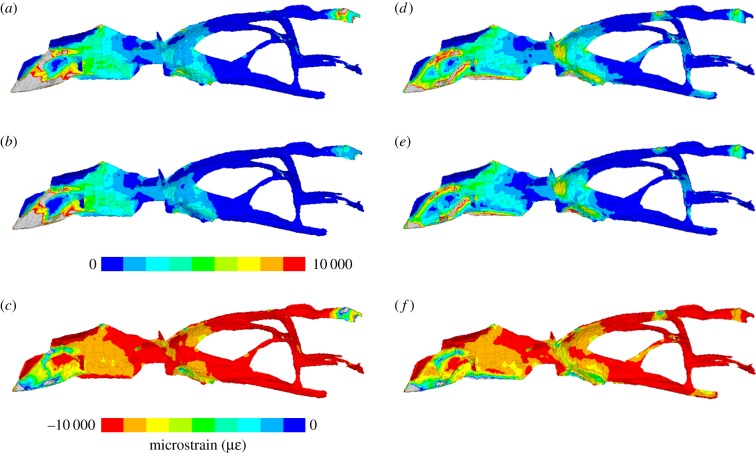
Strain contour plots within the chondrocranium in left lateral view from a model with a Young's modulus value of 20 MPa in a cranium with sutures included. (*a–c*) Anterior biting and (*d–f*) posterior biting, (*a,d*) von Mises strain, (*b,e*) 1st principal strain and (*c,f*) 3rd principal strain.

In both load cases (anterior and posterior biting) for the cranium with sutures, the greatest strains were located anteriorly rather than posteriorly, and the greatest strains were tensile rather than compressive (figure 2). The pattern for 1st principal (tensile) strains was very similar to that of von Mises strains but for 3rd principal (compressive) strains the difference in magnitude between strains located anteriorly and those located posteriorly was much less (particularly for posterior biting). During anterior (bilateral) biting, the highest strain magnitudes occurred in the anterior 10% of the chondrocranium (figure 2*a–c*): the anteroventral end of the nasal capsule and nasal septum. Strains were also present within the nasal septum, interorbital septum and the posterior most ends of the paired taenia marginalis (figure 3*a–c*). During posterior biting (unilateral), strains in the nasal septum and posterior-most ends of the paired taenia marginalis were less than half the magnitude observed during anterior biting. However, magnitudes were higher in the portion of chondrocranium between 30% and 70% of its length (figure 2*e–g*), which includes the interorbital septum (figure 3*d–f*). Also during posterior biting, in the region of the pila metoptica there is a slight increase in tensile strain (figure 3*d–f*).

### 3.2. Finite element results: cranium under anterior loading

**Figure 4.**
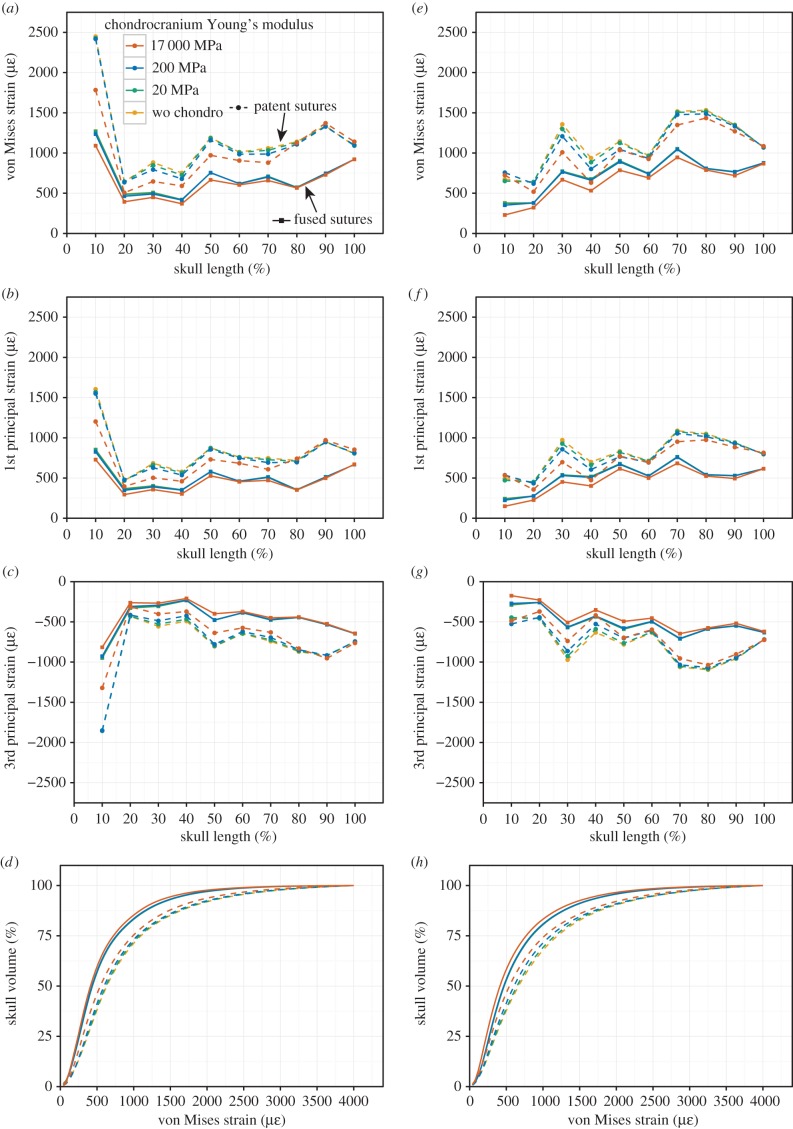
Strain plots for the cranium during (*a–d*) anterior biting and (*e–h*) posterior unilateral biting. (*a,e*) von Mises strain, (*b,f*) 1st principal strain, (*c,g*) 3rd principal strain and (*d,h*) cumulative strain plots. Dashed line = patent sutures.

**Figure 5.**
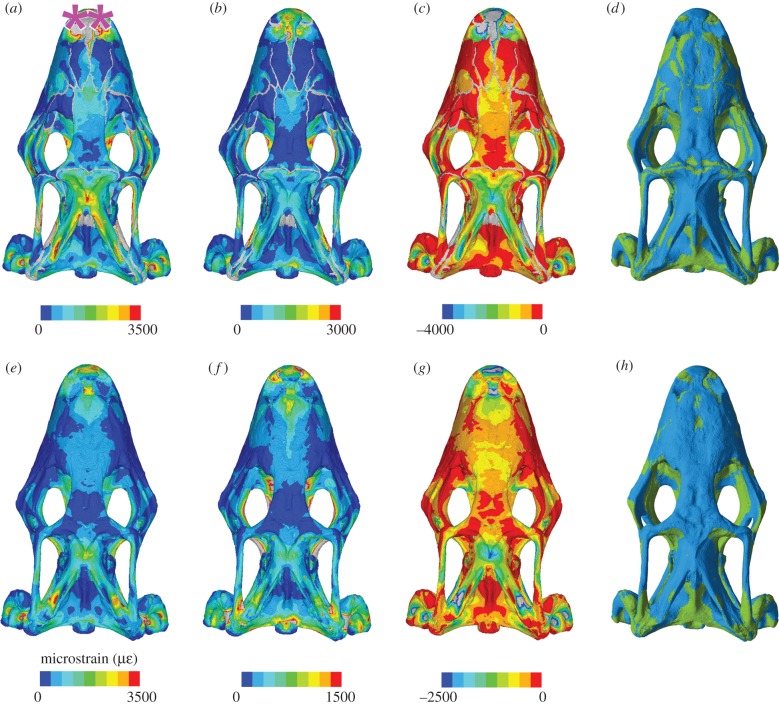
Strain contour plots and tension–compression dominance plots of the cranium under anterior loading in dorsal view with a chondrocranium of 200 MPa and sutures present (*a–d*) and absent (*e–h*) showing: von Mises strain (*a,e*), 1st principal strains (*b,f*), 3rd principal strains (*c,g*) and plots of whether strains are dominated by tension (green) or compression (blue). The pink asterisks indicate the location along the tooth row at which loading was applied (*a–h*). In the contour plots, strain values are given in microstrain and the sutures are shown in grey (*a–c* and *e–g*).

During anterior (bilateral) biting in the models without sutures the greatest values of von Mises strain occur in the anterior 20% of the cranium: strain was twice that of most other sections and three times that of the section between 20% and 40% of the length of the cranium (figure 4*a–c*). Specific regions of high strain include compression within the premaxilla and anterior end of the maxilla (near the location of the bite) as well as in the parietal (figure 5), and tension in the midpoint of the vomers, palatines, base of the braincase (basioccipital), the posterior ends of the pterygoids and around the pterygoid–quadrate joints (electronic supplementary material, figure S15).

Inclusion of cranial sutures resulted in greater strain magnitudes so that the total volume of skull bone that underwent more than 500 microstrain was approximately 60% rather than approximately 43% (figure 4*d*). The extent of von Mises strain and first principal strain along the anterior 70% of cranium length was accentuated and there was a relatively large increase in strain within the section between 80% and 90% along the length of the cranium (figure 4*a–c*). Specific locations of increased strain included the premaxilla, the facial process of the maxilla, the jugal (suborbital rim, postorbital bar), the anterior part of the frontal, the upper temporal bar (postorbital-squamosal), the vomers, the palatines and the posterior ends of the pterygoids (figure 5; electronic supplementary material, figures S13–S21 and S39). However, there was a decrease in strain in the lateral parts of the nasals, posterior ventral end of the frontal, the squamosals and the heads of the quadrates (figure 5; electronic supplementary material, figures S13–S21 and S39).

In both models, with and without sutures, the cranium roof was dominated by compression (electronic supplementary material, figure S33), whereas the palate and sphenoid were primarily under tension (electronic supplementary material, figure S34), indicating sagittal bending of the whole cranium. Areas of high tensile strain included the postorbital bar, the upper temporal bar, the vomers and the palatines, whereas high compressive strains were found in the premaxilla and along the lateral edges of the posterior processes of the pterygoids (electronic supplementary material, figures S13–S21 and S33 and S35–S36).

Addition of a chondrocranium made little difference to strain magnitudes within sections along the length of the cranium (figure 4*a–c*) or to general strain distribution on the bone surface (electronic supplementary material, figures S13–S21) except in the model with sutures where the chondrocranium was modelled as bone (17 000 MPa). Here, the levels of strain were 10–30% lower in the anterior 80% of the cranium compared to strain levels in other models with sutures (figure 4*a–c*). These results are not unexpected given that the additional bone along the midline of the model will make it more resistant to strain. The pattern of strain distribution in the cranium with a chondrocranium modelled at 20 MPa was very similar to those modelled without a chondrocranium. Those with a chondrocranium modelled at 200 MPa showed slightly lower strains (less than 10%) in some parts of the cranium compared to the cranium with no chondrocranium (figure 4*a–c*), perhaps most obviously in the premaxilla and nasals (electronic supplementary material, figures S17 and S21). Modelling the chondrocranium as bone rather than cartilage also altered the dominance of tension versus compression in some areas (electronic supplementary material, figures S31–S34), e.g. the area dominated by tension at the posterior end of the frontal is larger, whereas those in the nasals and anterior end of the frontal diminish (electronic supplementary material, figures S31 and S32).

### 3.3. Finite element results: cranium under posterior loading

During posterior biting (unilateral on the left side) in the models without sutures the greatest overall strain values occurred in the section 70–80% along the length of the cranium (just behind the postorbital bar), whereas the lowest strain values occurred in the anterior 20% of the cranium (figure 4*e–g*). Strain was also high at 30%: the location of the bite point. The highest strain during posterior biting was located in the left facial process of the maxilla (above the location of the bite), left pterygoid, left upper temporal bar, base of the braincase, body of the right palatine and right parietal (figure 6). The unilateral posterior biting resulted in very high (compressive) strains in the contralateral side of the parietal, whereas the ipsilateral side experienced only low strains (figure 6; electronic supplementary material, figures S22–S308).

**Figure 6.**
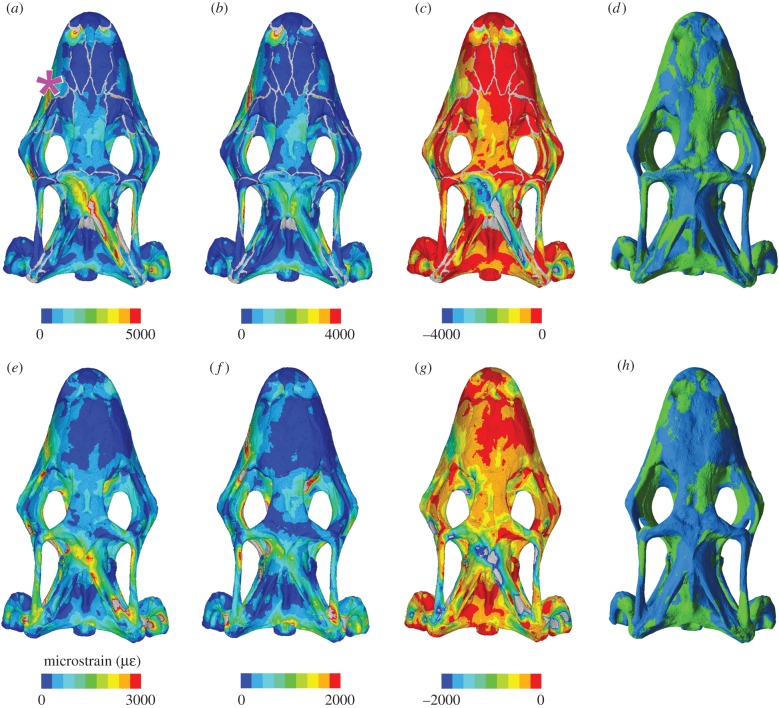
Strain contour plots and tension–compression dominance plots of the cranium under posterior loading in dorsal view with a chondrocranium of 200 MPa and sutures present (*a–d*) and absent (*e–h*) showing: von Mises strain (*a,e*), 1st principal strains (*b,f*), 3rd principal strains (*c,g*) and plots of whether strains are dominated by tension (green) or compression (blue). The pink asterisk indicates the location along the tooth row at which loading was applied (*a–h*). In the contour plots, strain values are given in microstrain and the sutures are shown in grey (*a–c* and *e–g*).

As with anterior biting, the sphenoid and most of the palate were primarily under tension, whereas the cranium roof showed large areas of compression, albeit with a notably asymmetrical strain distribution (figure 6; electronic supplementary material, figures S35–S38). Tension dominated the nasal and lateral face of the maxilla on the biting side, parts of the frontal on the opposite side, and the roof of the braincase (electronic supplementary material, figure S41). Among the models with sutures there was also an asymmetry in the loading of the parietal processes with the ipsilateral process under substantial compression (figure 6). Compression also dominated within the ventral portion of left maxilla above the bite point, the right nasal, the left prefrontal, the left side of the frontal and the posterior process of the left pterygoid (electronic supplementary material, figures S27, S30, S38 and S41).

Consistent with anterior biting, the inclusion of cranial sutures increased strain so that the total volume of skull bone that underwent at least 500 microstrain was much greater when sutures were included (60%) than when they were not (approx. 45%) (figure 4*h*). The von Mises strain increased in all sections along the cranium but not to the same extent (figure 4*e–g*). The greatest proportional increase in strain occurred in the section where the bite occurred, at 30% along the length of the cranium (from approx. 750 to 1250 microstrain), and where the jaw muscles were located in the posterior 30% of the cranium (from approx. 750 to 1500). Strains within the posterior section were greater during posterior biting than they were during anterior biting (1500 versus 1400), but strain in the anterior most section was about three times less (750 versus 2500) (figure 4*e–g*).

With sutures present, the strain differences within the parietal and upper temporal bars were accentuated (figure 6; electronic supplementary material, figure S41), and strain was greater in the anteroventral portion of the left maxilla, the facial process of the right maxilla and the lateral process of the left pterygoid. However, strains within the left prefrontal, nasal, lacrimal, postorbital, squamosal and quadrate, as well as the right palatine and right pterygoid were relatively lower (electronic supplementary material, figures S22–S24).

The addition of a chondrocranium again had little effect on the bone strain magnitudes regardless of material properties used (figure 4*e–g*). The strains in a cranium with a chondrocranium modelled at 20 MPa were very slightly lower than one modelled with no chondrocranium. When the chondrocranium was modelled at 200 MPa, strains were lower in the central part of the cranium but it increased slightly at the anterior tip. When the chondrocranium was modelled as bone (17 000 MPa) strains were again lower in the sections representing 20–40% and 70–90% along the cranium (figure 4*e–g*).

## 4. Discussion

Representation of soft-tissue structures in biomechanical models is critical for a more complete understanding of skull mechanics [41,46,49,51,68,84], but it is important to identify which tissues have a significant role. Here, we analysed the role of the sutures and chondrocranium in a lizard cranial model.

Strains within the chondrocranium of *S. merianae* (as modelled) were twice as great during anterior biting compared to posterior biting. This result likely reflects the vulnerability of this mid-sagittal structure to the greater long-axis sagittal bending of the skull during anterior versus posterior biting [18]. Our model was given isotropic material properties, although the nasal septum and trabeculae cranii were modelled as thicker than the interorbital and orbito-temporal regions. Nevertheless, even with posterior bites, strains within the posterior part of the chondrocranium (80–100% along the full length) were often less than half of those of the anterior part (0–70% along the full length). This suggests that, in life, the anterior part of the chondrocranium may have different material properties to the posterior part. This has yet to be investigated in detail in lizards, but Klenner *et al*. [20] found differences in histology between different parts of the septum nasi and septum interorbitale in *Caiman crocodilus*. In pigs, the anterior nasal septum was found to have a higher compressive stiffness and lower tensile stiffness than the posterior portion [18] which should result in lower equivalent compressive strains. Similarly, in our model of the lizard chondrocranium, the highest strains are observed to be tensile rather than compressive. If this is also true *in vivo*, then the lizard nasal septum is unlikely to be acting primarily as a compressive bracing structure for the rostrum, as has been suggested for mammals [20,25,26] where much of it ossifies in adults. Nevertheless, in lizards with mesokinesis (flexion at the fronto-parietal suture), compressive strain might be predicted to dominate the chondrocranium, particularly in the ventral part of the orbito-temporal region.

The observation that strain distribution within the chondrocranium appears to be sensitive to bite location may help to explain the variable degrees of mineralization that occur in different squamate taxa, for example, the ossification of the pila metoptica as an orbitosphenoid. However, although there is an increase in strain in the pila metoptica and posterior part of the taenia marginalis (figure 1) in posterior biting, the increase appears to be relatively minor (figures 2 and 3) and other factors are likely to play a role. Our models did not include the eye muscles that attach to the orbital cartilage and are probably a source of additional strain [21]. We also focused on an adult specimen of a relatively large robust lizard that might not be representative of juvenile specimens or those with a high degree of intracranial flexibility [29].

Overall, therefore, our analyses have shown that the inclusion of a cartilaginous chondrocranium made little difference to strain magnitudes in the overlying cranial bones, irrespective of biting location (figure 2*a*). However, as in previous studies [49,51] but with a more detailed model, we have shown that the presence of cranial sutures increases overall strain within the cranium, and reduces areas of very low strain in which bone maintenance could be compromised. Sutures alter strain distribution qualitatively (not just quantitatively) to the extent that comparisons across species using models that do not include sutures may be of limited value. Our results are consistent with the hypothesis that a relatively even strain distribution exists in life and is necessary for appropriate growth and bone turnover [46,85]. During anterior biting in the model without sutures almost all the palate and ventral brain case were under tension, whereas the cranial roof was in compression (figure 5). The cranium essentially behaved like a simple beam under sagittal bending. In contrast, when sutures are present there is a more complex distribution of tension and compression: the structure is split into several components, resulting in some areas of tension on the dorsal surface of the cranium. This more complex pattern is consistent with *in vivo* strain results found for other vertebrate taxa (e.g. [43,44]).

Despite growing evidence that sutures have a significant role in skull biomechanics (e.g. [42–44,49,51]), they are frequently not included within analyses due a persistent perception that they may not be important enough to justify modelling their complex three-dimensional anatomy. Wang *et al*. [86] is often cited as an example of an analysis where inclusion of sutures did not affect strain distribution. However, this study focused on primates which have fewer sutures than most reptiles [86] and possess globe-like skulls rather than frame-like skulls [32,51]. Moreover, the model used by Wang *et al*. [86] only included eight facial sutures; the many sutures in the basicranium and cranial vault were ignored so that the face was essentially attached around its perimeter to a much larger, rigid structure. We are, therefore, not confident that this study provides conclusive evidence that sutures have no significant biomechanical role in vertebrate skulls. Moreover, we urge greater effort to include sutures within biomechanical models (particularly when studying reptiles) so that progress can be made towards investigating them fully.

Our results provide some insights into the functional morphology of *S. merianae*, a taxon that is known to actively predate other vertebrates [57]. Posterior bites deliver the greatest bite forces [52]. The high compressive strains that we observed in the contralateral parietal process during unilateral posterior biting suggest that these processes play an important role in supporting the skull from the torsion associated with this type of biting (figure 6). The orientation of the parietal processes within the skull (in dorsal view) sits along a line between posterior teeth, where high bite forces are generated, and the base of the contralateral quadrate where the joint reaction forces occur. The principal strain plots show peak dominant compressive strains in the contralateral side of the parietal, but the tensile strains are generally lower. Given that the length, orientation and cross-sectional shape of these processes varies considerably among squamate taxa [2], this is clearly a potentially informative area for further research.

## 5. Conclusion

Our results do not support suggestions from work on other taxa that the nasal septum in lizards functions as a supporting structure within the nasal cavity [15,25,26] or that it acts as a stress dampener [18,27,28]. As modelled in the South American tegu lizard, *S. merianae*, the chondrocranium mainly experiences tension but its inclusion does not significantly reduce strain from surrounding bones. Strain within the chondrocranium is unevenly distributed and differs according to bite position, a finding that may explain differences in mineralization among different species but this requires further investigation. Without sutures, the cranium behaves like a simple beam under sagittal bending but when sutures are included and the cranium is split up into several connected components, tensile strain is observed in some areas on the dorsal surface. Our results are consistent with previous studies that show inclusion of sutures can radically alter strain distribution, indicating that, in life, sutures may be important in ensuring that all regions of the skull experience a threshold level of strain for bone maintenance [51]. Incomplete representation of soft tissues appears to be a likely reason why some models are unable to replicate strains measured *ex vivo* or *in vivo*.

## Supplementary Material

Additional figures of the models and contour plots

## Supplementary Material

SI Table 1

## Supplementary Material

SI Table 2

## Data accessibility

Electronic supplementary material, additional figures: further images of the segmented cranium as well as strain contour and tension–compression dominance plots in dorsal, ventral and lateral view. Electronic supplementary material, table S1: muscle characteristics used in the MDA and FEA. Electronic supplementary material, table S2: comparison between bite force estimates from multi-body dynamics, finite-element analysis and *in vivo* measurements.

## References

[1] Schwenk K 2000 Feeding in lepidosaurs. In Feeding form, function, and evolution in tetrapod vertebrates (ed. Schwenk K), pp. 175–291. San Diego, CA: Academic Press.

[2] Evans SE 2008 The skull of lizards and tuatara. In Biology of the Reptilia, vol. 20, Morphology H: the skull of Lepidosauria (eds Gans C, Gaunt AS, Adler K), pp. 1–344. Ithaca, NY: Society for the Study of Amphibians and Reptiles.

[3] Daza JD, Diogo R, Johnston P, Abdala V 2011 Jaw adductor muscles across lepidosaurs: a reappraisal. Anat. Rec. 294, 1765–1782. (doi:10.1002/ar.21467) 10.1002/ar.21467 21901848

[4] Rayfield EJ 2007 Finite element analysis and understanding the biomechanics and evolution of living and fossil organisms. Annu. Rev. Earth Planet. Sci. 35, 541–576. (doi:10.1146/annurev.earth.35.031306.140104)

[5] Moazen M, Curtis N, Evans SE, O'Higgins P, Fagan MJ 2008 Rigid-body analysis of a lizard skull: modelling the skull of *Uromastyx hardwickii*. J. Biomech. 41, 1274–1280. (doi:10.1016/j.jbiomech.2008.01.012) 18308322 10.1016/j.jbiomech.2008.01.012

[6] Moazen M, Curtis N, Evans SE, O'Higgins P, Fagan MJ 2008 Combined finite element and multibody dynamics analysis of biting in a *Uromastyx hardwickii* lizard skull. J. Anat. 213, 499–508. (doi:10.1111/j.1469-7580.2008.00980.x) 19014357 10.1111/j.1469-7580.2008.00980.x PMC2667544

[7] Curtis N 2011 Craniofacial biomechanics: an overview of recent multibody modelling studies. J. Anat. 218, 16–25. (doi:10.1111/j.1469-7580.2010.01317.x) 21062283 10.1111/j.1469-7580.2010.01317.x PMC3039777

[8] Marcé-Nogué J, Kłodowski A, Sánchez M, Gil L 2015 Coupling finite element analysis and multibody system dynamics for biological research. Palaeontol. Electron. 18, 1–14.

[9] Bellairs A, Kamal AM 1981 The chondrocranium and the development of the skull in recent reptiles. In Biology of the Reptilia, Development, vol. 11 (eds Gans C, Parsons TS), pp. 1–264. London, UK: Academic Press.

[10] De Beer GR 1930 The early development of the chondrocranium of the lizard. Quart. J. Microp. Sci. 73, 606–739.

[11] De Beer GD 1937 The development of the vertebrate skull. Oxford, UK: Oxford University Press.

[12] Schoch RR 2006 Skull ontogeny: developmental patterns of fishes conserved across major tetrapod clades. Evol. Dev. 8, 524–536. (doi:10.1111/j.1525-142X.2006.00125.x) 17073936 10.1111/j.1525-142X.2006.00125.x

[13] Kamal AM, Abdeen AM 1972 The development of the chondrocranium of the lacertid lizard, *Acanthodactylus boskiana*. J. Morphol. 137, 289–334. (doi:10.1002/jmor.1051370304) 10.1002/jmor.1051370304 30366483

[14] Sánchez-Villagra M, Forasiepi AM 2017 On the development of the chondrocranium and the histological anatomy of the head in perinatal stages of marsupial mammals. Zool. Lett. 3, 1–33. (doi:10.1186/s40851-017-0062-y) 10.1186/s40851-017-0062-y PMC5303607 28203388

[15] Kemble JVH 1973 Underdevelopment of the maxilla related to absence of the cartilaginous nasal septum. Br. J. Plast. Surg. 26, 266–270. (doi:10.1016/0007-1226(73)90012-X) 4726106 10.1016/0007-1226(73)90012-x

[16] Gange RJ, Johnston LE 1974 The septopremaxillary attachment and midfacial growth: an experimental study on the albino rat. Am. J. Orthod. 66, 71–81. (doi:10.1016/0002-9416(74)90194-8) 4525652 10.1016/0002-9416(74)90194-8

[17] Copray JC 1986 Growth of the nasal septal cartilage of the rat *in vitro*. J. Anat. 144, 99–111. 3693052 PMC1166466

[18] Al Dayeh AA, Herring SW 2014 Compressive and tensile mechanical properties of the porcine nasal septum. J. Biomech. 40, 154–161. (doi:10.1016/j.jbiomech.2013.09.026) 10.1016/j.jbiomech.2013.09.026 PMC3910320 24268797

[19] Xia Y, Zheng S, Szarko M, Lee J 2012 Anisotropic properties of bovine nasal cartilage. Microsc. Res. Tech. 75, 300–306. (doi:10.1002/jemt.21058) 21823202 10.1002/jemt.21058 PMC3222710

[20] Klenner S, Witzel U, Paris F, Distler C 2016 Structure and function of the septum nasi and the underlying tension chord in crocodylians. J. Anat. 228, 113–124. (doi:10.1111/joa.12404) 26552989 10.1111/joa.12404 PMC4694159

[21] Pearson HS 1921 The skull and some related structures of a late embryo of *Lygosoma*. J. Anat. 56, 20–43. 17103934 PMC1262962

[22] Zada S 1981 The fully formed chondrocranium of the agamid lizard, *Agama pallida*. J. Morphol. 170, 43–54. (doi:10.1002/jmor.1051700104) 7288886 10.1002/jmor.1051700104

[23] Hugi J, Mitgutsch C, Sánchez-Villagra MR 2010 Chondrogenic and ossification patterns and sequences in White's skink *Liopholis whitii* (Scincidae, Reptilia). Zoosyst. Evol. 86, 21–32. (doi:10.1002/zoos.200900011)

[24] Hernández-Jaimes C, Jerez A, Ramírez-Pinilla MP 2012 Embryonic development of the skull of the Andean lizard *Ptychoglossus bicolor* (Squamata, Gymnophthalmidae). J. Anat. 221, 285–302. (doi:10.1111/j.1469-7580.2012.01549.x) 22881276 10.1111/j.1469-7580.2012.01549.x PMC3458248

[25] Moss ML, Bromberg BE, Song IG, Eisenman G 1968 The passive role of nasal septal cartilage in mid-facial growth. Plastic. Recon. Surg. 41, 536–542. (doi:10.1097/00006534-196806000-00004) 10.1097/00006534-196806000-00004 5654895

[26] Stenström SJ, Thilander BL 1970 Effects of nasal septal cartilage resections on young guinea pigs. Plastic. Recon. Surg. 45, 160–170. (doi:10.1097/00006534-197002000-00010) 10.1097/00006534-197002000-00010 5411897

[27] Al Dayeh AA, Raffery KL, Egbert M, Herring SW 2009 Deformation of nasal septal cartilage during mastication. J. Morphol. 270, 1209–1218. (doi:10.1002/jmor.10750) 19434723 10.1002/jmor.10750 PMC2786896

[28] Lee SJ, Liong K, Lee HP 2010 Deformation of nasal septum during nasal trauma. Laryngoscope 120, 1931–1939. (doi:10.1002/lary.21072) 20824645 10.1002/lary.21072

[29] Hallermann J 1992 Morphological significance of the orbitotemporal region in amphikinetic skulls of juvenile iguanians (Squamata). Zool. J. Anat. 122, 203–206.

[30] Simões TR, Funston GF, Vafaeian B, Nydam RL, Caldwell MW 2016 Reacquisition of the lower temporal bar in sexually dimorphic fossil lizards provides a rare case of convergent evolution. Sci. Rep. 6, 24087 (doi:10.1038/srep24087) 27071447 10.1038/srep24087 PMC4829860

[31] Herring SW 2000 Sutures and craniosynostosis: a comparative, functional and evolutionary perspective. In Craniosynostosis (eds Cohen MM, MacLean RE), pp. 3–10, 2nd edn Oxford, UK: Oxford University Press.

[32] Jones MEH, Curtis N, Fagan MJ, O'Higgins P, Evans SE 2011 Hard tissue anatomy of the cranial joints in *Sphenodon* (Rhynchocephalia): sutures, kinesis, and skull mechanics. Palaeontol. Electron. 14, 1–92.

[33] Opperman LA 2000 Cranial sutures as intramembranous bone growth sites. Dev. Dynam. 219, 472–485. (doi:10.1002/1097-0177(2000)9999:9999<::AID-DVDY1073>3.0.CO;2-F) 10.1002/1097-0177(2000)9999:9999<::AID-DVDY1073>3.0.CO;2-F 11084647

[34] Sarnat BG 2008 Some factors related to experimental snout growth. J. Craniofac. Surg. 19, 1308–1314. (doi:10.1097/SCS.0b013e3181843532) 18812856 10.1097/SCS.0b013e3181843532

[35] Weickenmeier J, Fischer C, Carter F, Kuhl E, Goriely A 2017 Dimensional, geometrical, and physical constraints in skull growth. Phys. Rev. Lett. 118, 1–5. (doi:10.1103/PhysRevLett.118.248101) 10.1103/PhysRevLett.118.248101 28665667

[36] Payne SL, Holliday CM, Vickaryous MK 2011 An osteological and histological investigation of cranial joints in geckos. Anat. Rec. 294, 399–405. (doi:10.1002/ar.21329) 10.1002/ar.21329 21254447

[37] Mezzasalma M, Maio N, Guarino FM 2014 To move or not to move: cranial joints in European gekkotans and lacertids, an osteological and histological perspective. Anat. Rec. 297, 463–472. (doi:10.1002/ar.22827) 10.1002/ar.22827 24550139

[38] Herrel A, De Vree F, Delheusy V, Gans C 1999 Cranial kinesis in gekkonid lizards. J. Exp. Biol. 202, 3687–3698. 10574746 10.1242/jeb.202.24.3687

[39] Montuelle SJ, Williams SH 2015 *In vivo* measurement of mesokinesis in *Gekko gecko*: the role of cranial kinesis during gape display, feeding and biting. PLoS ONE 10, e0134710 (doi:10.1371/journal.pone.0134710) 26230087 10.1371/journal.pone.0134710 PMC4521707

[40] Jaslow CR 1990 Mechanical properties of cranial sutures. J. Biomech. 23, 313–321. (doi:10.1016/0021-9290(90)90059-C) 2335529 10.1016/0021-9290(90)90059-c

[41] Kupczik K, Dobson CA, Fagan MJ, Crompton RH, Oxnard CE, O'Higgins P 2007 Assessing mechanical function of the zygomatic region in macaques: validation and sensitivity testing of finite element models. J. Anat. 210, 41–53. (doi:10.1002/ar.21415) 17229282 10.1111/j.1469-7580.2006.00662.x PMC2100262

[42] Rafferty KL, Herring SW 1999 Craniofacial sutures: morphology, growth, and *in vivo* masticatory strains. J. Morphol. 242, 167–179. (doi:10.1016/S1095-6433(01)00472-X) 10521876 10.1002/(SICI)1097-4687(199911)242:2<167::AID-JMOR8>3.0.CO;2-1 PMC2813870

[43] Rafferty KL, Herring SW, Marshall CD 2003 Biomechanics of the rostrum and the role of facial sutures. J. Morphol. 257, 33–44. (doi:10.1002/jmor.10104) 12740894 10.1002/jmor.10104 PMC2819158

[44] Markey MJ, Main RP, Charles RM 2006 *In vivo* cranial suture function and suture morphology in the extant fish *Polypterus*: implications for inferring skull function in living and fossil fish. J. Exp. Biol. 209, 2085–2102. (doi:10.1242/jeb.02266) 16709911 10.1242/jeb.02266

[45] Moreno K, Wroe S, Clausen P, McHenry C, D'Amore DC, Rayfield EJ, Cunningham E 2008 Cranial performance in the Komodo dragon (*Varanus komodoensis*) as revealed by high-resolution 3-D finite element analysis. J. Anat. 212, 736–746. (doi:10.1111/j.1469-7580.2008.00899.x) 18510503 10.1111/j.1469-7580.2008.00899.x PMC2423397

[46] Curtis N, Jones MEH, Shi J, O'Higgins P, Evans SE, Fagan MJ 2011 Functional relationship between skull form and feeding mechanics in *Sphenodon*, and implications for diapsid skull development. PLoS ONE 6, e29804 (doi:10.1371/journal.pone.0029804) 22216358 10.1371/journal.pone.0029804 PMC3247290

[47] McCurry MR, Mahony M, Clausen PD, Quayle MR, Walmsley CW, Jessop TS, Wroe S, McHenry CR 2015 The relationship between cranial structure, biomechanical performance and ecological diversity in varanoid lizards. PLoS ONE 10, e0130625 (doi:10.1371/journal.pone.0130625) 26106889 10.1371/journal.pone.0130625 PMC4479569

[48] Openshaw GH, D'Amore DC, Vidal-Garcia M, Keogh JS 2017 Combining geometric morphometric analysis of multiple 2D observation views improves interpretation of evolutionary allometry and shape diversification in monitor lizard (*Varanus*) crania. Biol. J. Linn. Soc. 120, 539–552. (doi:10.1111/bij.12899)

[49] Moazen M, Curtis N, O'Higgins P, Jones MEH, Evans SE, Fagan MJ 2009 Assessment of the role of sutures in a lizard skull: a computer modelling study. Proc. R. Soc. B 276, 39–46. (doi:10.1098/rspb.2008.0863) 10.1098/rspb.2008.0863 PMC2614251 18765341

[50] Moazen M, Costantini D, Bruner E 2013 A sensitivity analysis to the role of the fronto-parietal suture in *Lacerta bilineata*: a preliminary finite element study. Anat. Rec. 296, 198–209. (doi:10.1002/ar.22629) 10.1002/ar.22629 23192831

[51] Curtis N, Jones MEH, Evans SE, O'Higgins P, Fagan MJ 2013 Cranial sutures work collectively to distribute strain throughout the reptile skull. J. R. Soc. Interface 10, 20130442 (doi:10.1098/rsif.2013.0442) 23804444 10.1098/rsif.2013.0442 PMC3730698

[52] Gröning F, Jones MEH, Curtis N, Herrel A, O'Higgins P, Evans SE, Fagan MJ 2013 The importance of accurate muscle modelling for biomechanical analyses: a case study with a lizard skull. J. R. Soc. Interface 10, 20130216 (doi:10.1098/rsif.2013.0216) 23614944 10.1098/rsif.2013.0216 PMC3673157

[53] Harvey MB, Ugueto GN, Gutberlet RL 2012 Review of teiid morphology with a revised taxonomy and phylogeny. Zootaxa 3459, 1–156. (doi:10.7934/P759)

[54] Tucker DB, Colli GR, Giugliano LG, Hedges SB, Hendry CR, Lemmon EM, Lemmon AR, Sites JW Jr, Pyron RA 2016 Methodological congruence in phylogenomic analyses with morphological support for teiid lizards (Sauria: Teiidae). Mol. Phyl. Evol. 103, 75–84. (doi.org/10.1016/j.ympev.2016.07.002) 10.1016/j.ympev.2016.07.002 27395779

[55] Presch W 1974 A survey of the dentition of the macroteiid lizards (Teiidae: Lacertilia). Herpetologica 30, 344–349.

[56] Dalrymple GH 1979 On the jaw mechanism of the snail-crushing lizards, *Dracaena* Daudin 1802 (Reptilia, Lacertilia, Teiidae). J. Herpetol. 13, 303–311. (doi:10.2307/1563324)

[57] Colli GR, Péres AK Jr, da Cunha HJ 1998 A new species of *Tupinambis* (Squamata: Teiidae) from Central Brazil, with an analysis of morphological and genetic variation in the genus. Herpetologica 54, 477–492.

[58] Fabre AC, Cornette R, Huyghe K, Andrade DV, Herrel A 2014 Linear versus geometric morphometric approaches for the analysis of head shape dimorphism in lizards. J. Morphol. 275, 1016–1026. (doi:10.1002/jmor.20278) 24740578 10.1002/jmor.20278

[59] Hsiou AS 2007 A new Teiidae species (Squamata, Scincomorpha) from the Late Pleistocene of Rio Grande do Sul State, Brazil. Rev. Bras. Paleontol. 10, 181–194. (doi:10.4072/rbp.2007.3.05)

[60] Hsiou AS, Schubert BW, Winck GR, Wujionary-Alves S, Avilla LS 2016 New quaternary teiid (Lepidosauria, Squamata) lizard remains from Gruta do Urso, Tocantins, Brazil. Rev. Bras. Paleontol. 19, 233–242. (doi:10.4072/rbp.2016.2.07)

[61] Elias JA, McBrayer LD, Reilly SM 2000 Prey transport kinematics in *Tupinambis teguixin* and *Varanus exanthematicus*: conservation of feeding behavior in ‘chemosensory-tongued’ lizards. J. Exp. Biol. 203, 791–801. 10648221 10.1242/jeb.203.4.791

[62] Montuelle SJ, Herrel A, Schaerlaeken V, Metzger KA, Mutuyeyezu A, Bels VL 2009 Inertial feeding in the teiid lizard *Tupinambis merianae*: the effect of prey size on the movements of hyolingual apparatus and the cranio-cervical system. J. Exp. Biol. 212, 2501–2510. (doi:10.1242/jeb.026336) 19648393 10.1242/jeb.026336

[63] Tattersall GJ, Leite CAC, Sanders CE, Cadena V, Andrade DV, Abe AS, Milsom WK 2016 Seasonal reproductive endothermy in tegu lizards. Sci. Adv. 2, e1500951 (doi:10.1126/sciadv.1500951) 26844295 10.1126/sciadv.1500951 PMC4737272

[64] Rieppel O 1980 The trigeminal jaw adductor musculature of *Tupinambis*, with comments on the phylogenetic relationships of the Teiidae (Reptilia, Lacertilia). Zool. J. Linn. Soc. 69, 1–29. (doi:10.1111/j.1096-3642.1980.tb01930.x)

[65] Schaerlaeken V, Holanova V, Boistel R, Aerts P, Velensky P, Rehak I, Andrade DV, Herrel A 2012 Built to bite: feeding kinematics, bite forces, and head shape of a specialized durophagous lizard, *Dracaena guianensis* (Teiidae). J. Exp. Zool. 317A, 371–381. (doi:10.1002/jez.1730) 10.1002/jez.1730 22610877

[66] Curtis N, Jones MEH, Evans SE, Shi J, O'Higgins P, Fagan MJ 2010 Predicting muscle activation patterns from motion and anatomy: modelling the skull of *Sphenodon* (Diapsida: Rhynchocephalia). J. R. Soc. Interface 7, 153–160. (doi:10.1098/rsif.2009.0139) 19474084 10.1098/rsif.2009.0139 PMC2839385

[67] Jones MEH, O'Higgins P, Fagan M, Evans SE, Curtis N 2012 Shearing mechanics and the influence of a flexible symphysis during oral food processing in *Sphenodon* (Lepidosauria: Rhynchocephalia). Anat. Rec. 295, 1075–1091. (doi:10.1002/ar.22487) 10.1002/ar.22487 22644955

[68] Gröning F, Fagan MJ, O'Higgins P 2011 The effects of the periodontal ligament on mandibular stiffness: a study combining finite element analysis and geometric morphometrics. J. Biomech. 44, 1304–1312. (doi:10.1016/j.jbiomech.2011.01.008) 21292267 10.1016/j.jbiomech.2011.01.008

[69] Davis JL, Dumont ER, Strait DS, Grosse IR 2011 An efficient method of modeling material properties using a thermal diffusion analogy: an example based on craniofacial bone. PLoS ONE 6, e17004 (doi:10.1371/journal.pone.0017004) 21347288 10.1371/journal.pone.0017004 PMC3037934

[70] Chamoli U, Wroe S 2011 Allometry in the distribution of material properties and geometry of the felid skull: why larger species may need to change and how they may achieve it. J. Theoret. Biol. 283, 217–226. (doi:10.1016/j.jtbi.2011.05.020) 21651916 10.1016/j.jtbi.2011.05.020

[71] Cuff AR, Bright JA, Rayfield EJ 2015 Validation experiments on finite element models of an ostrich (*Struthio camelus*) cranium. Peer J. 3, e1294 (doi:10.7717/peerj.1294) 26500813 10.7717/peerj.1294 PMC4614885

[72] Ferguson VL, Bushby AJ, Boyde A 2003 Nanomechanical properties and mineral concentration in articular calcified cartilage and subchondral bone. J. Anat. 203, 191–202. (doi:10.1046/j.1469-7580.2003.00193.x) 12924819 10.1046/j.1469-7580.2003.00193.x PMC1571155

[73] Porter ME, Beltran JL, Koob TJ, Summer AP 2006 Material properties and biochemical composition of mineralized vertebral cartilage in seven elasmobranch species (Chondrichthyes). J. Exp. Biol. 209, 2920–2928. (doi:10.1242/jeb.02325) 16857876 10.1242/jeb.02325

[74] Burgin LV 2003 Impact loading of articular cartilage. PhD thesis, University of Aberdeen. 10.1053/joca.2002.0803 12127840

[75] Edelsten L, Jeffrey JE, Burgin LV, Aspden RM 2010 Viscoelastic deformation of articular cartilage during impact loading. Soft Matter 6, 5206–5212. (doi:10.1039/c0sm00097c)

[76] Columbo V, Čadová M, Gallo LM 2013 Mechanical behaviour of bovine nasal cartilage under static and dynamic loading. J. Biomech. 46, 2137–2144. (doi:10.1016/j.jbiomech.2013.07.001) 23915577 10.1016/j.jbiomech.2013.07.001

[77] Burgin LV, Edelsten L, Aspden RM 2014 The mechanical and material properties of elderly human articular cartilage subject to impact and slow loading. Med. Engin. Phys. 36, 226–232. (doi:10.1016/j.medengphy.2013.11.002) 10.1016/j.medengphy.2013.11.002 24275561

[78] Peters AE, Comerford EJ, Macaulay S, Bates KT, Akhtar R 2017 Micromechanical properties of canine femoral articular cartilage following multiple freeze–thaw cycles. J. Mech. Behav. Biomed. Mat. 71, 114–121. (doi:10.1016/j.jmbbm.2017.03.006) 10.1016/j.jmbbm.2017.03.006 PMC5429396 28285060

[79] Flam E 1974 The tensile and flexural properties of bovine nasal cartilage. J. Biomed. Mater. Res. 8, 277–282. (doi:10.1002/jbm.820080509) 4426905 10.1002/jbm.820080509

[80] Griffin MF, Premakumar Y, Scifalian AM, Szarko M, Butler PEM 2016 Biomechanical characterisation of the human nasal cartilages; implications for tissue engineering. J. Mater. Sci. Mater. Med. 27, 1–6. (doi:10.1007/s10856-015-5619-8) 26676857 10.1007/s10856-015-5619-8 PMC4681753

[81] Jeffrey JE, Aspden RM 2006 The biophysical effects of a single impact load on human and bovine articular cartilage. J. Mech. E. 220, 677–686. (doi:10.1243/09544119JEIM31) 10.1243/09544119JEIM31 16961187

[82] Ahrens J, Gevici B, Law C 2005 Paraview: an end-user tool for large data visualization. In The visualization handbook (eds Hansen CD, Johnson CR), pp. 717–732. Burlington, NJ: Butterworth-Heinemann.

[83] R Development Core Team. 2008 R: a language and environment for statistical computing. Vienna, Austria: R Foundation for Statistical Computing.

[84] Curtis N, Witzel U, Fitton L, O'Higgins P, Fagan M 2011 The mechanical significance of the temporal fasciae in *Macaca fascicularis*: an investigation using finite element analysis. Anat. Rec. 294, 1178–1190. (doi:10.10.1002/ar.21415) 10.1002/ar.21415 21618443

[85] Martin RB 2000 Toward a unifying theory of bone remodeling. Bone 26, 1–6. (doi:10.1016/S8756-3282(99)00241-0) 10617150 10.1016/s8756-3282(99)00241-0

[86] Wang Q, Smith AL, Strait D, Wright BE, Richmond BG, Grosse IR, Byron CD, Zapata U 2010 The global impact of sutures assessed in a finite element model of a macaque cranium. Anat. Rec. 293, 1477–1491. (doi:10.1002/ar.21203) 10.1002/ar.21203 20652940

