# Supplementary material for: The biomechanical role of the chondrocranium and sutures in a lizard cranium
Source: J R Soc Interface. 2017 Dec 20;14(137):20170637. doi: 10.1098/rsif.2017.0637 (PMC5746569; doi:10.1098/rsif.2017.0637)
Supplement: Additional figures of the models and contour plots [file rsif20170637supp1.pdf]

Supplementary Information – additional figures

This document provides additional images of models and contour plots to improve context .

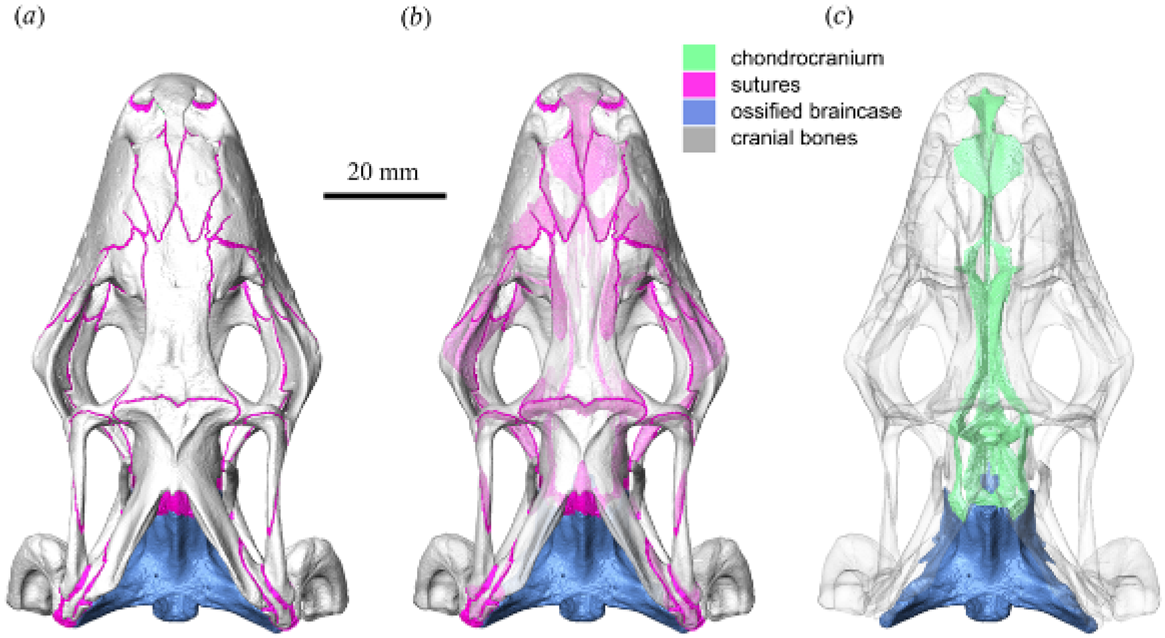

SI Figure 1. Fully segmented skull of *Salvator merianae* (=*Tupinambis merianae*) based on X-ray computed tomography in dorsal view with the cranial bones (a) opaque, (b) transparent to show the sutural overlaps, and (c) transparent to show detail of the chondrocranium. Scale bar = 20 mm.

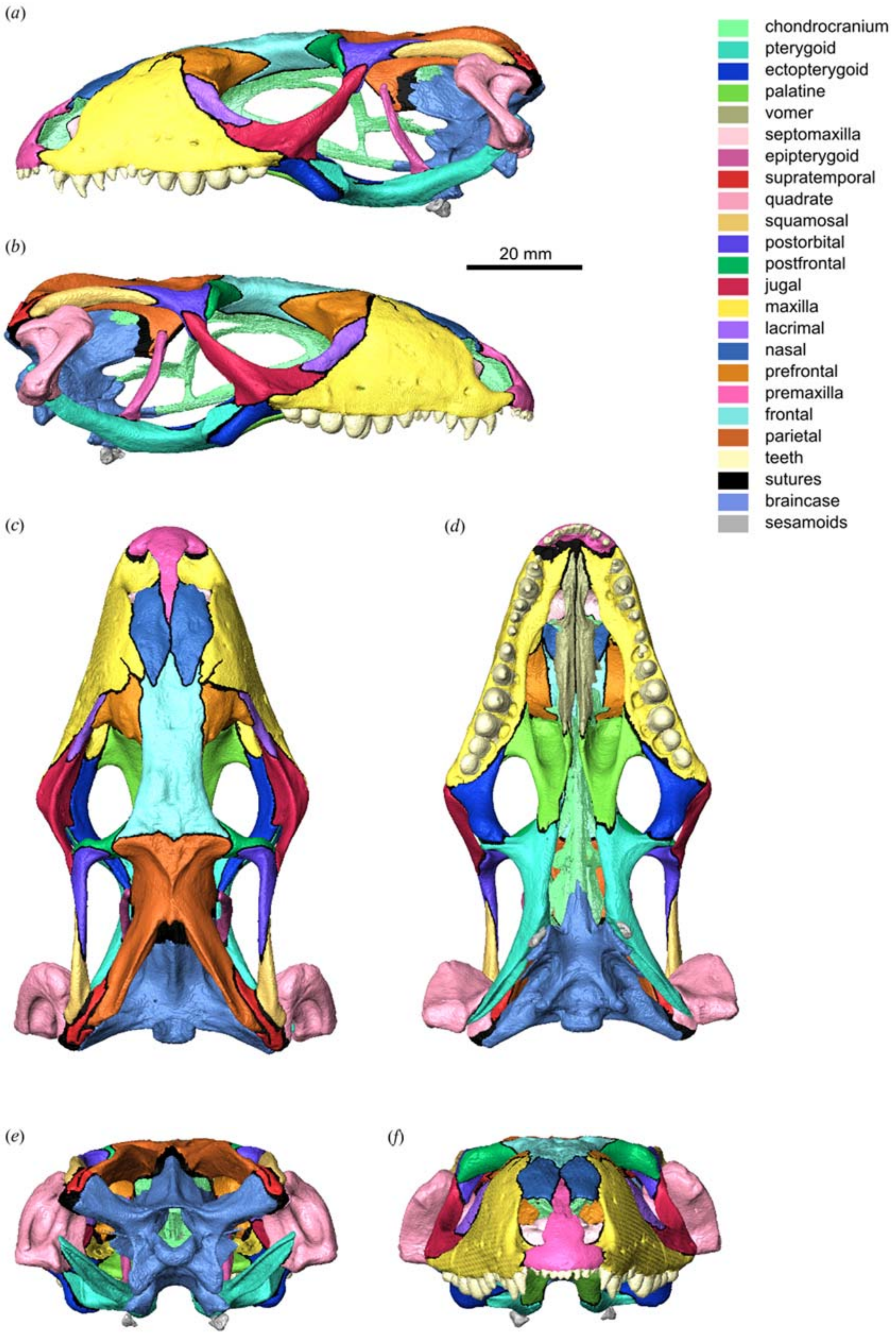

(a)
(b)
20 mm
(c)
(d)
(e)
(f)
chondrocranium
pterygoid
ectopterygoid
palatine
vomer
septomaxilla
epipterygoid
supratemporal
quadrate
squamosal
postorbital
postfrontal
jugal
maxilla
lacrimal
nasal
prefrontal
premaxilla
frontal
parietal
teeth
sutures
braincase
sesamoids

SI Figure 2. Fully segmented skull model of *Salvator* (=*Tupinambis*) *merianae* based on X-ray computed tomography in (a) left lateral, (b) right lateral, (c) dorsal, (d) ventral, (e) posterior, and (f) anterior view. Scale bar = 20 mm.

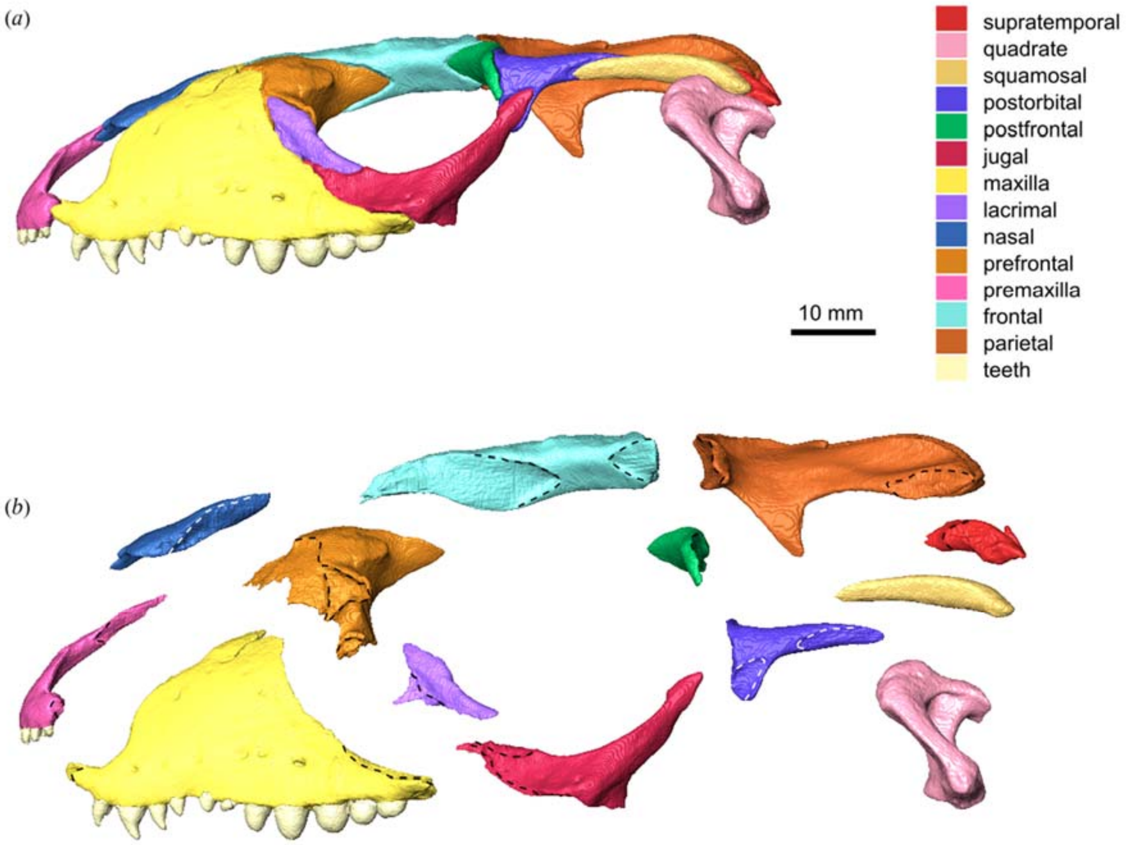

SI Figure 3. Most of the bones of the left half of the cranium in left lateral view when (a) articulated and (b) disarticulated. Scale bar = 10 mm.

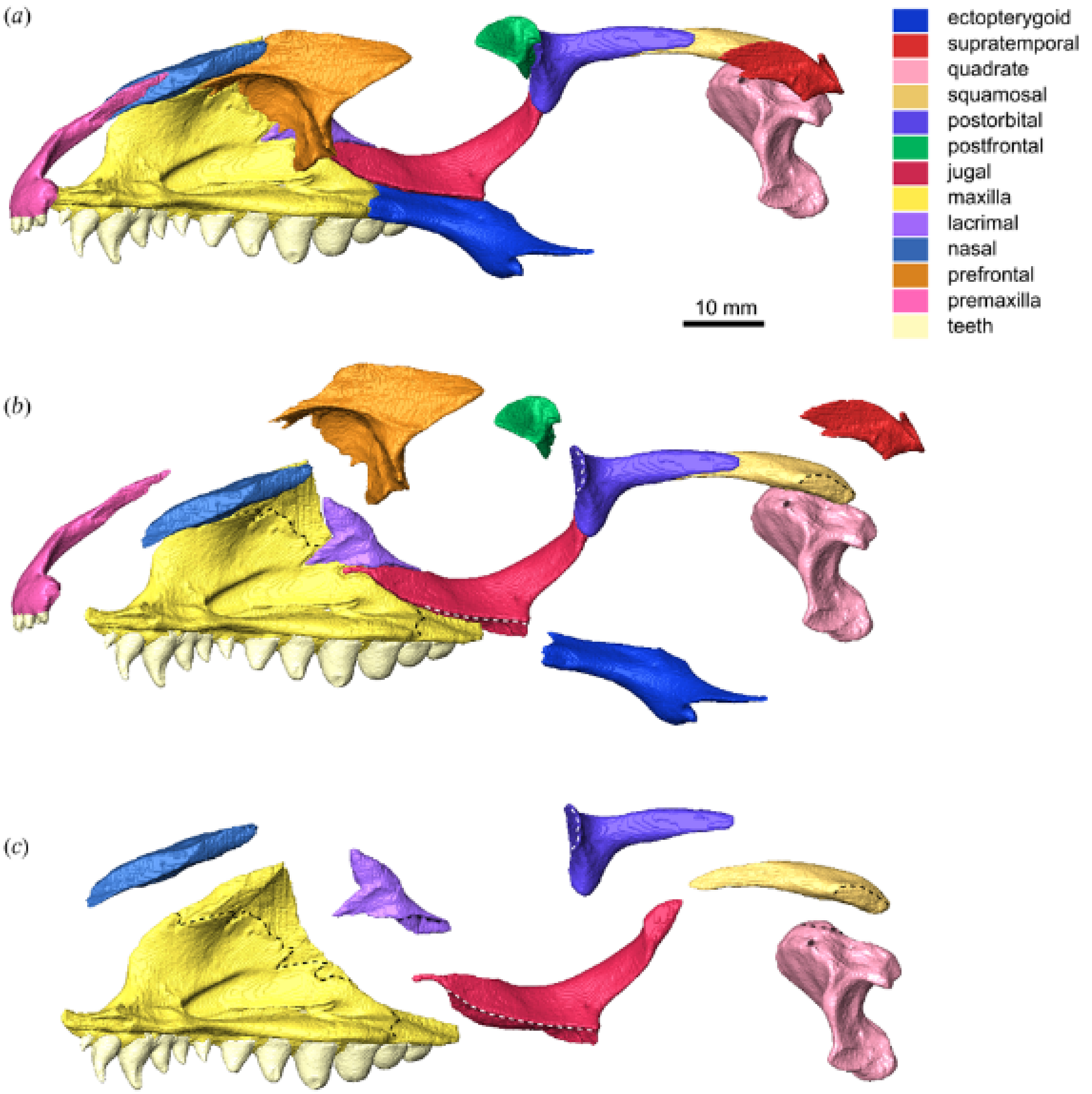

SI Figure 4. Most of the bones of the right half of the cranium in left medial view when (a) articulated, (b) partially disarticulated (b), and fully disarticulated. Scale bar = 10 mm.

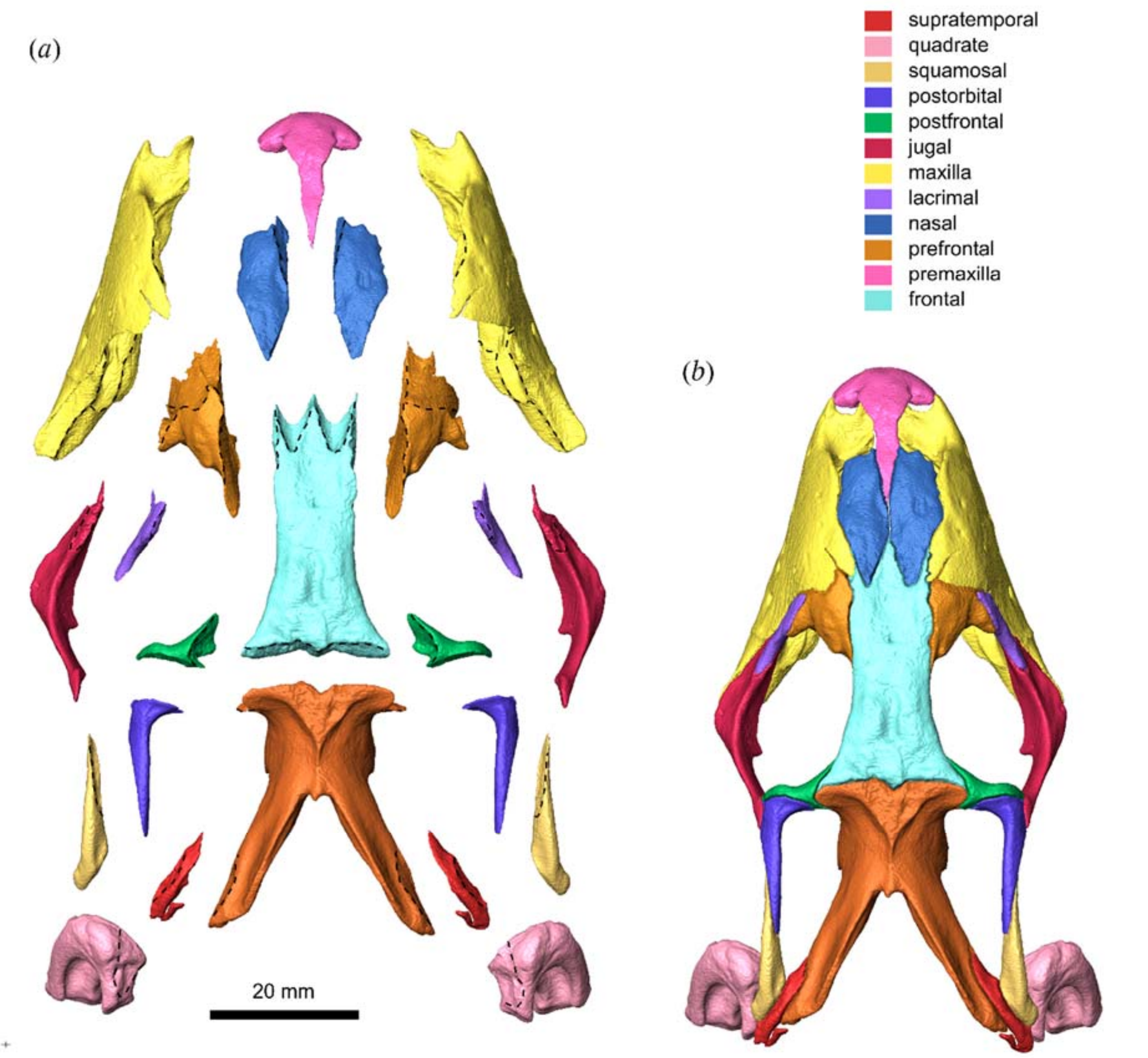

SI Figure 5. Most of the cranial bones as seen in dorsal view when (a) disarticulated and (b) articulated. Scale bar = 20 mm.

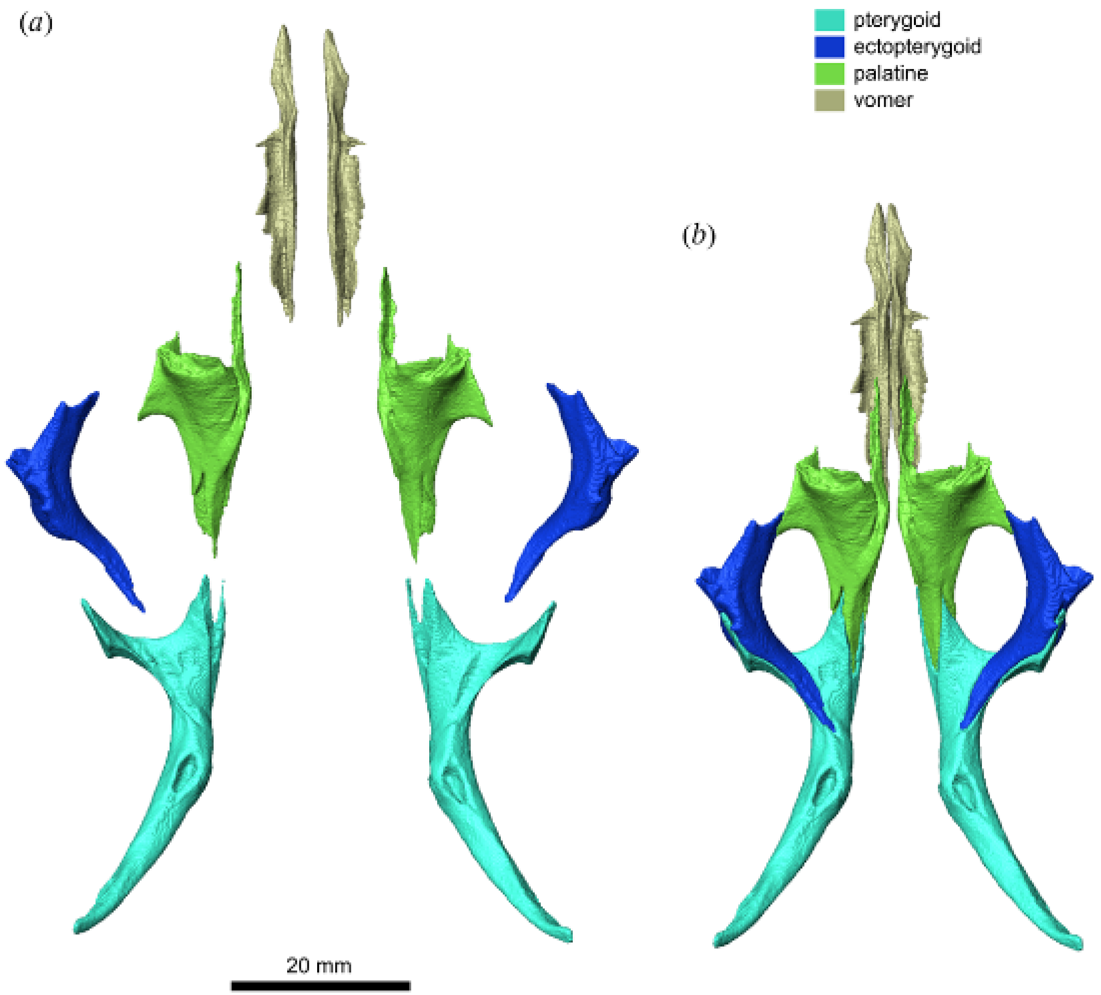

SI Figure 6. A dorsal view of the palate (a) disarticulated as well as in (b) articulation. Scale bar = 20 mm.

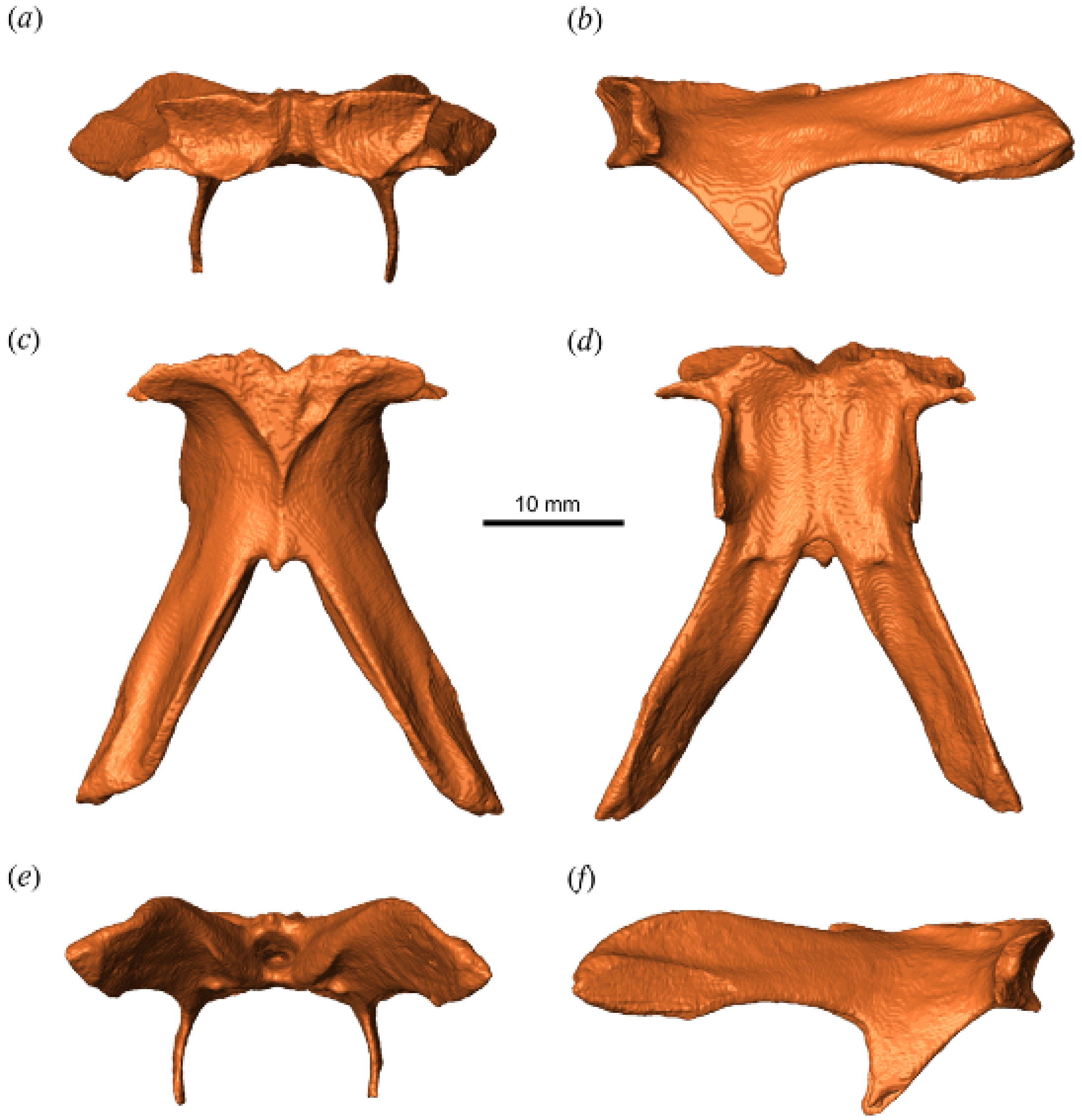

SI Figure 7. The parietal in (a) anterior, (b) left lateral, (c) dorsal, (d) ventral, (e) posterior, and (f) right lateral view. Scale bar = 10 mm.

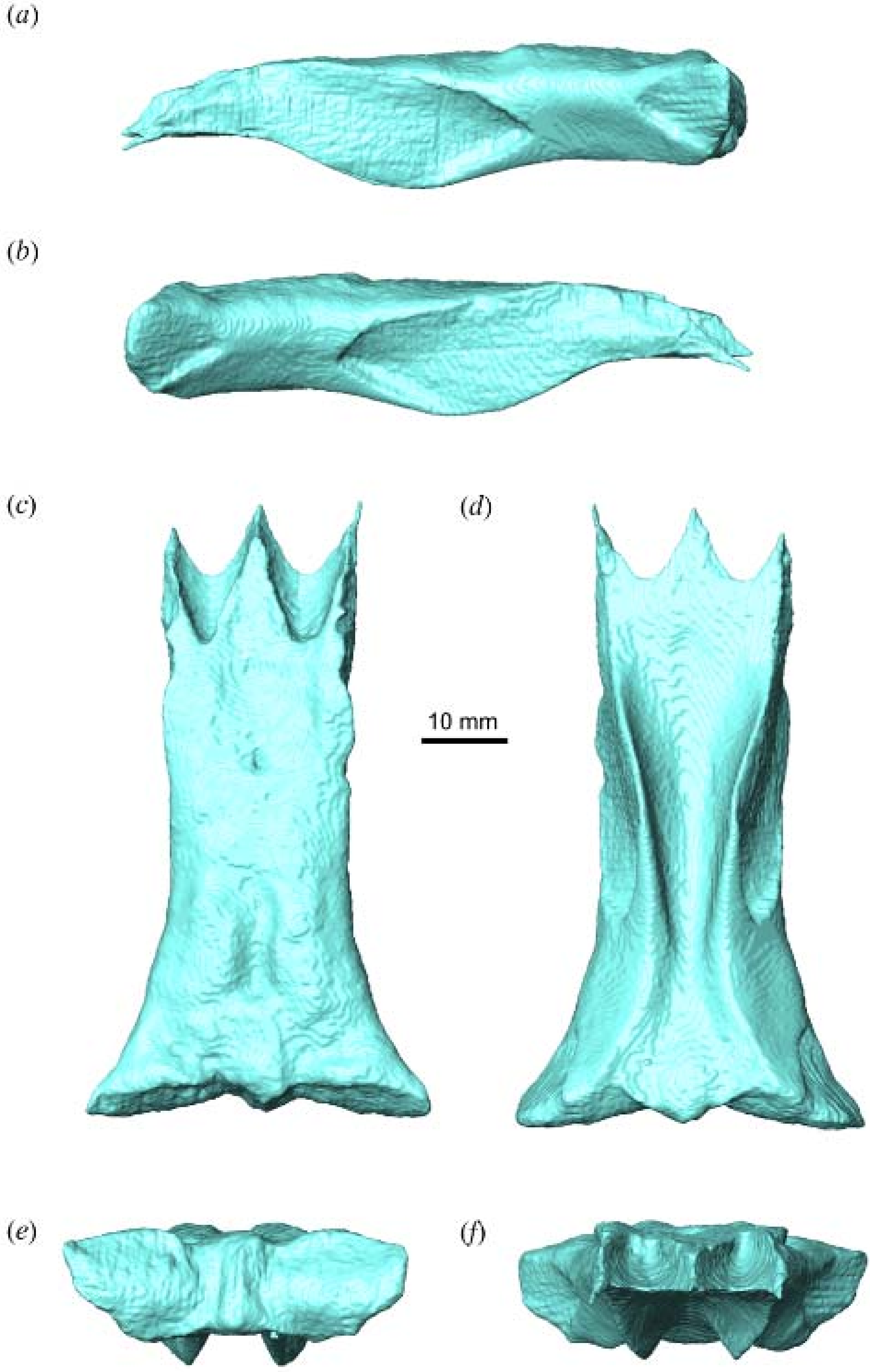

SI Figure 8. The frontal in (a) left lateral, (b) right lateral, (c) dorsal, (d) ventral, (e) posterior, and (f) anterior view. Scale bar = 10 mm.

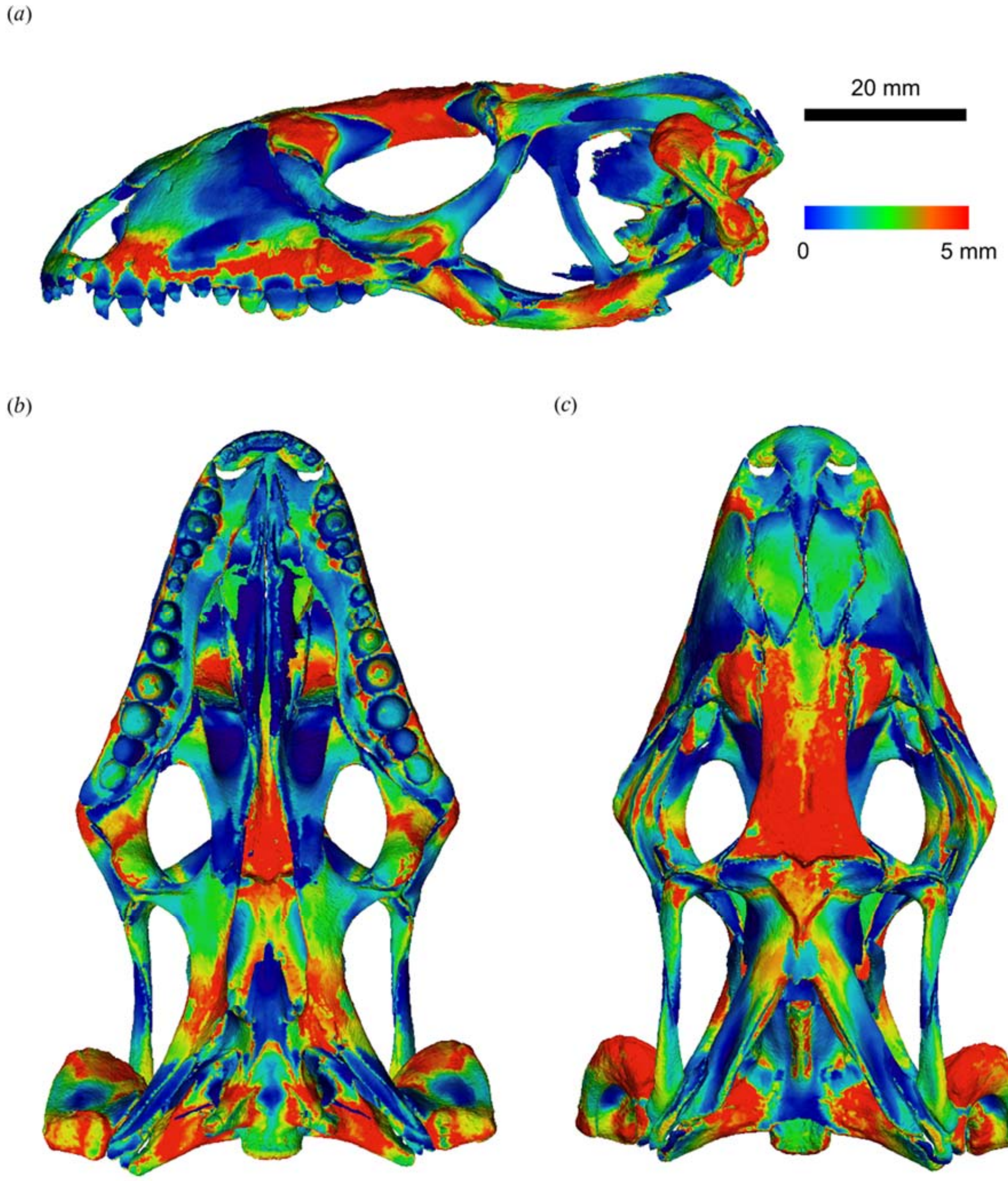

SI Figure 9. Thickness of the bones as modelled in the FEA. (a) left lateral, (b) ventral, (c) dorsal. Scale bar = 20 mm.

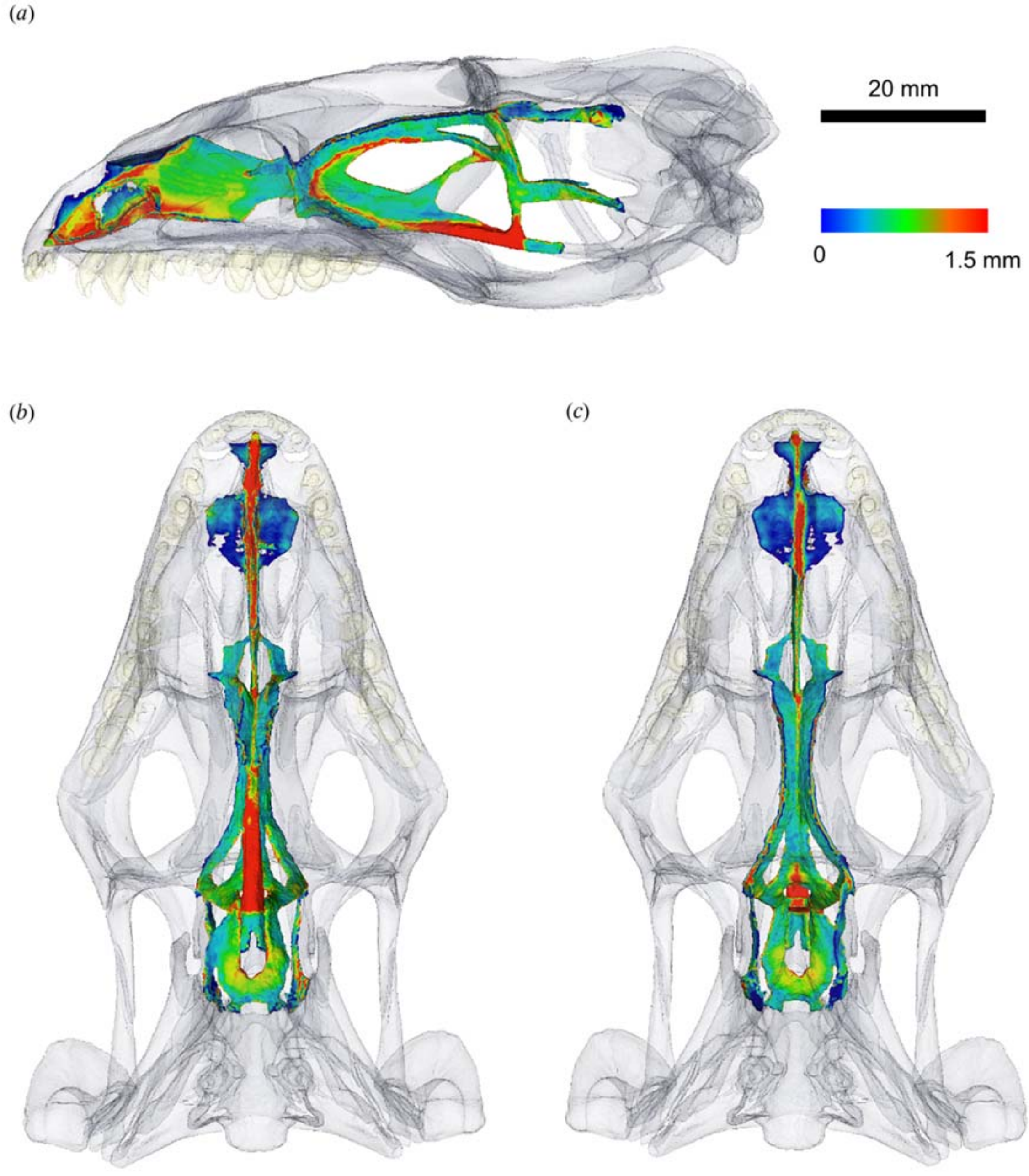

SI Figure 10. Thickness of the chondrocranium as modelled in the FEA. (a) left lateral, (b) ventral, (c) dorsal. The cranium and teeth are shown with 90% transparency. Scale bar = 20 mm.

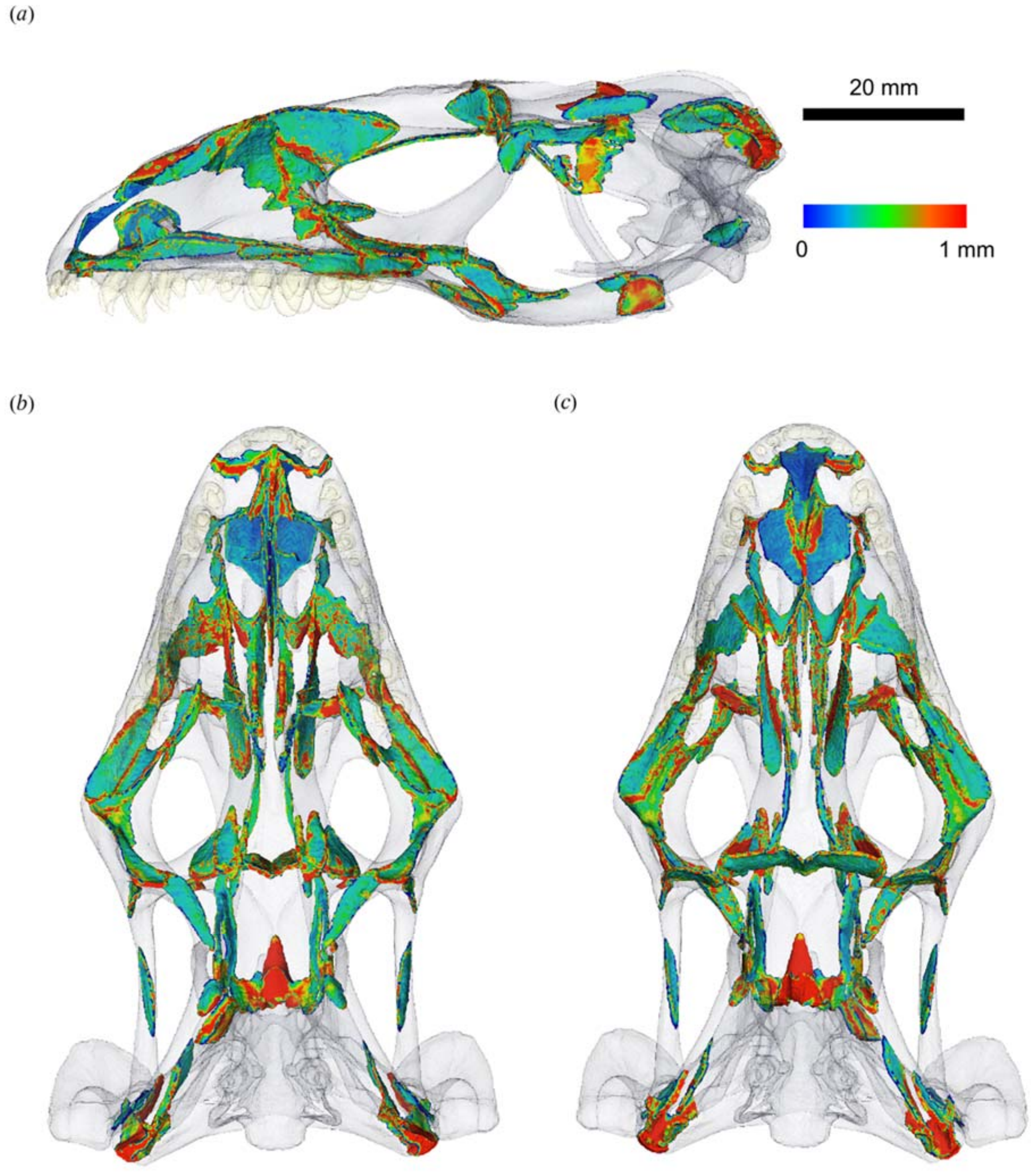

SI Figure 11. Thickness of the sutures as modelled in the FEA. (a) left lateral, (b) ventral, (c) dorsal. The cranium and teeth are shown with 90% transparency. Scale bar = 20 mm.

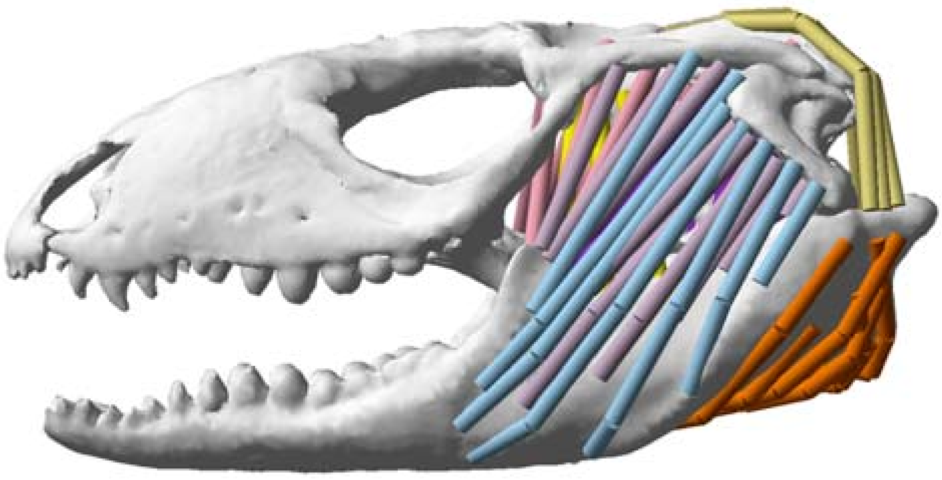

SI Figure 12. The multibody model as described in Gröning et al. (2013).

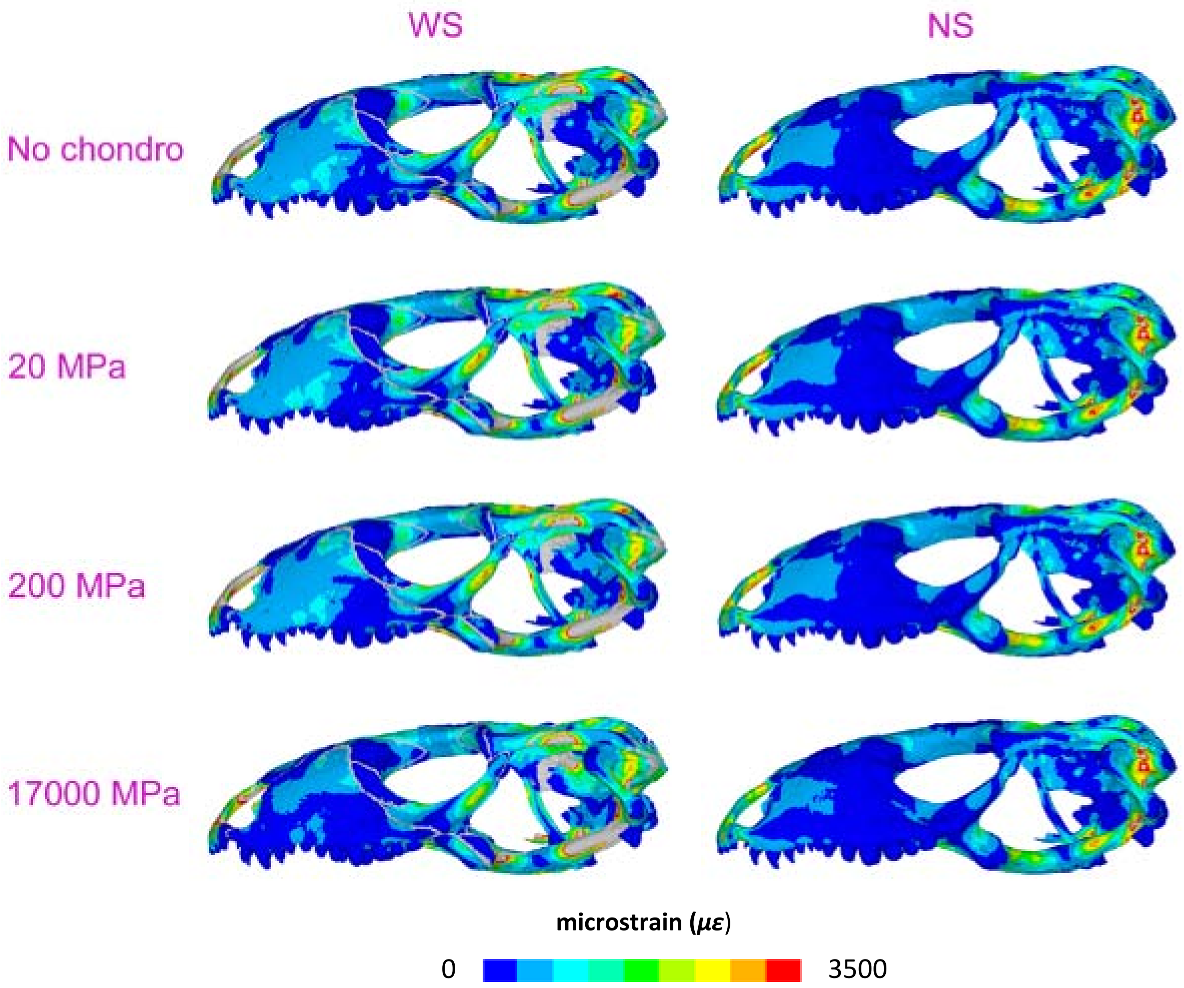

SI Figure 13. Contour plots of von Mises strain in the cranium under anterior biting in lateral view. The left hand column includes models with sutures (WS) and the right hand column includes models without sutures (NS). The first row includes models without a chondrocranium (no chondro) whereas the second, third, and fourth rows include models with a chondrocranium with a Young's modulus of 20 MPa, 200 MPa, and 17000 MPa respectively.

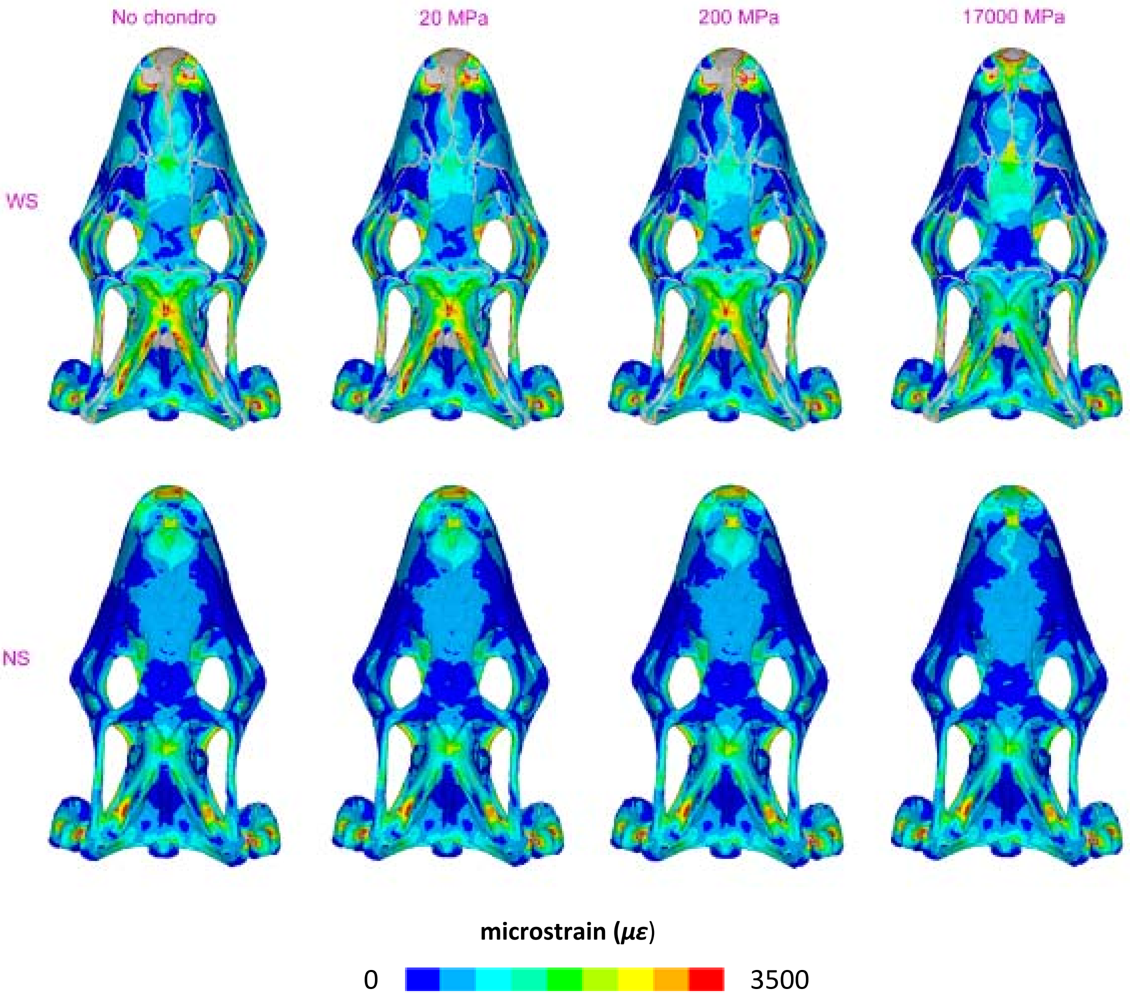

SI Figure 14. Contour plots of von Mises Strain in the cranium under anterior biting in dorsal view. The first row includes models with sutures (WS) and the second row models without sutures (NS). The left hand column includes models without a chondrocranium (no chondro) whereas the second, third, and fourth column include models with a chondrocranium with a Young's modulus of 20 MPa, 200 MPa, and 17000 MPa respectively.

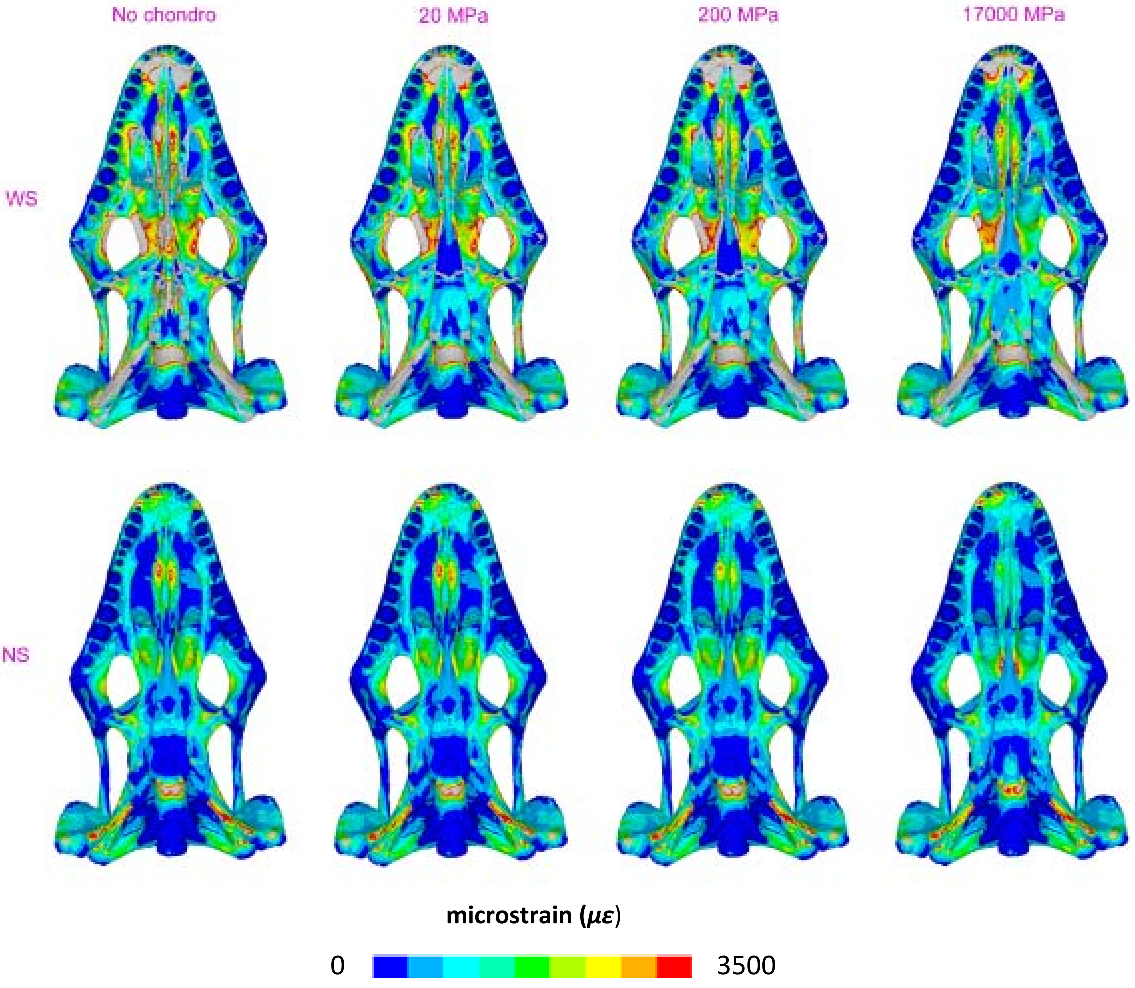

SI Figure 15. Contour plots of von Mises Strain in the cranium under anterior biting in ventral view.

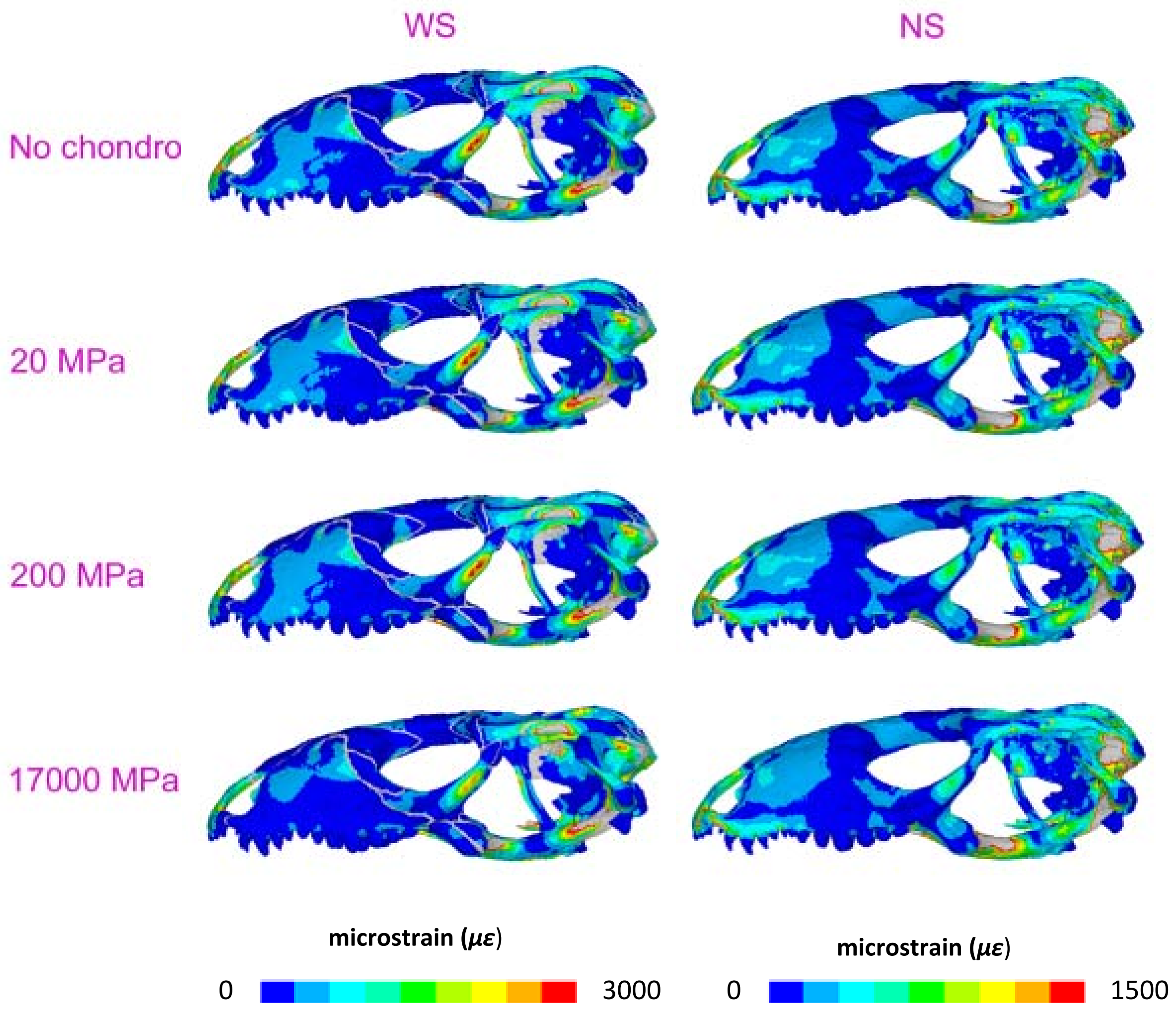

SI Figure 16. Contour plots of 1st principal strain in the cranium under anterior biting in lateral view.

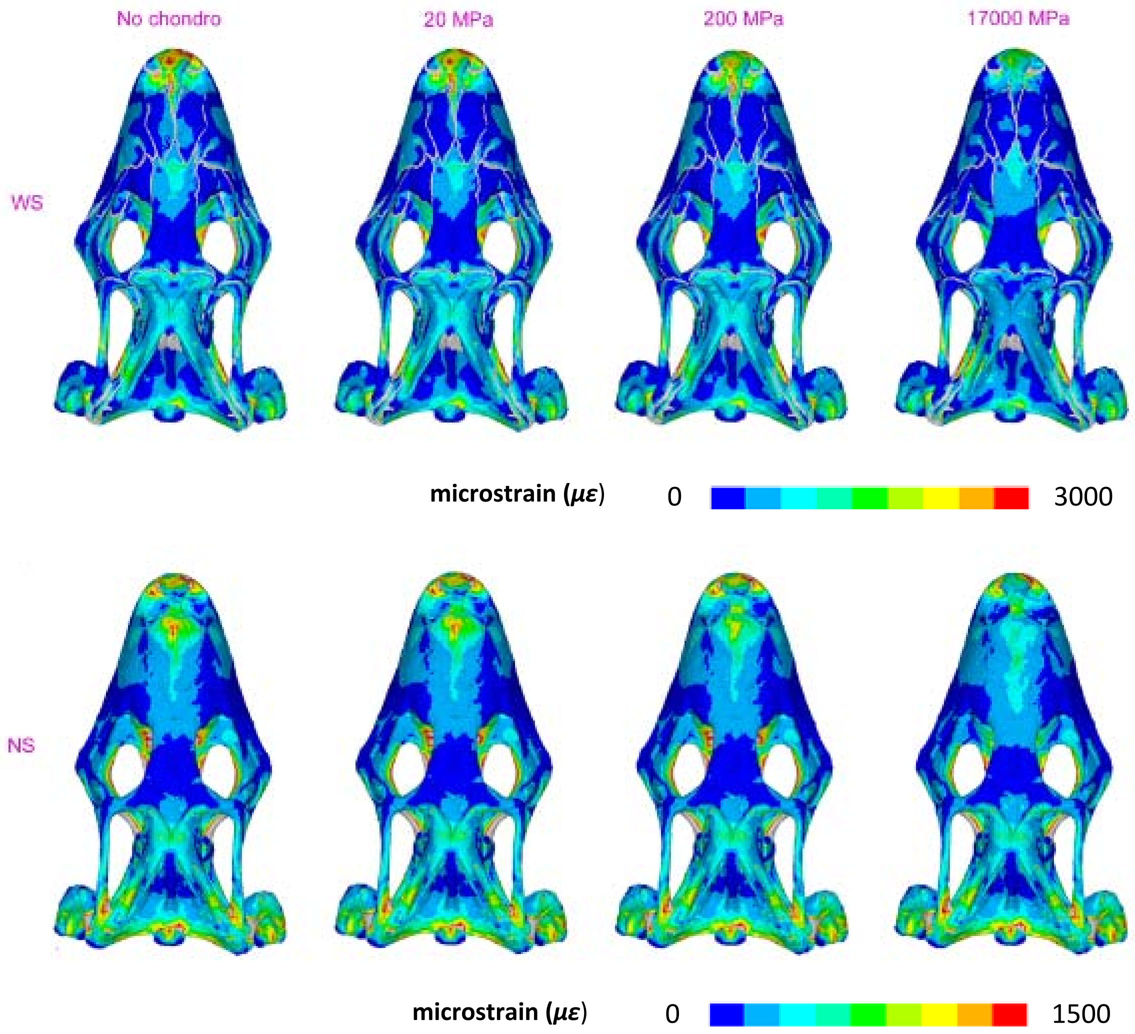

SI Figure 17. Contour plots of 1st principal strain in the cranium under anterior biting in dorsal view. Note that the strain magnitudes are different so that green in the models with sutures is equivalent to red in the models without sutures.

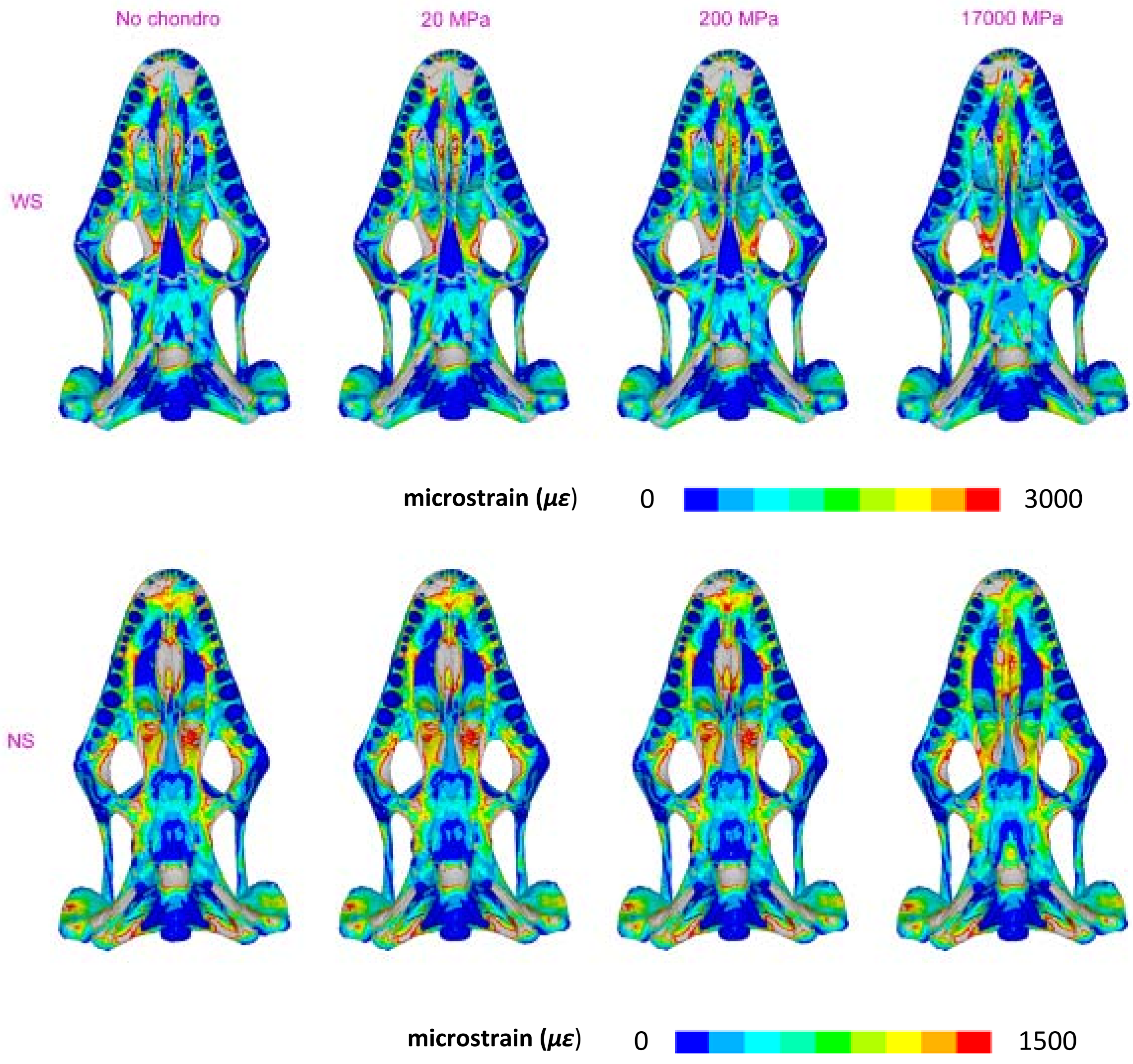

SI Figure 18. Contour plots of 1st principal strain in the cranium under anterior biting in ventral view. Note that the strain magnitudes are different so that green in the models with sutures is equivalent to red in the models without sutures.

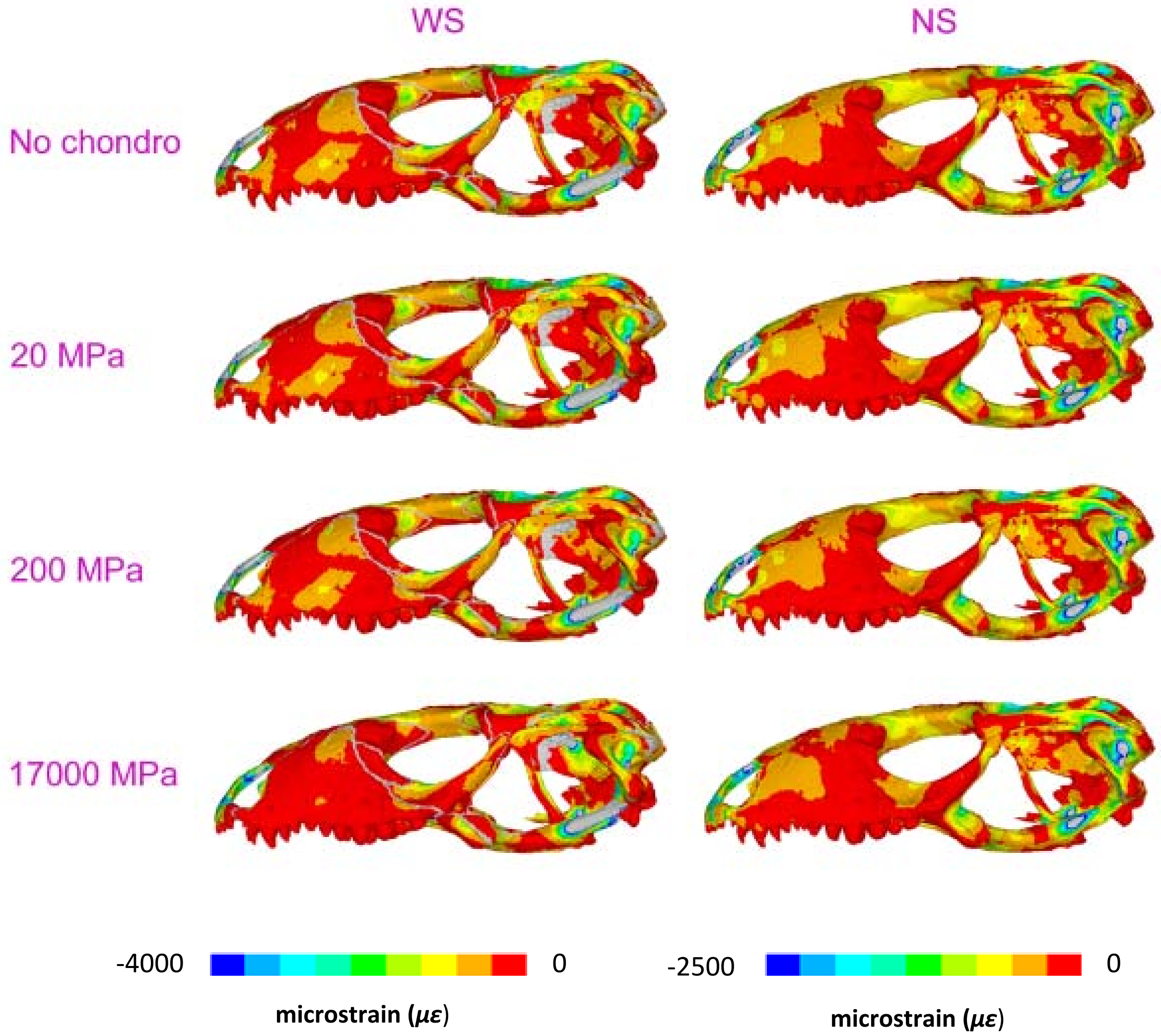

SI Figure 19. Contour plots of 3rd principal strain in the cranium under anterior biting in lateral view.

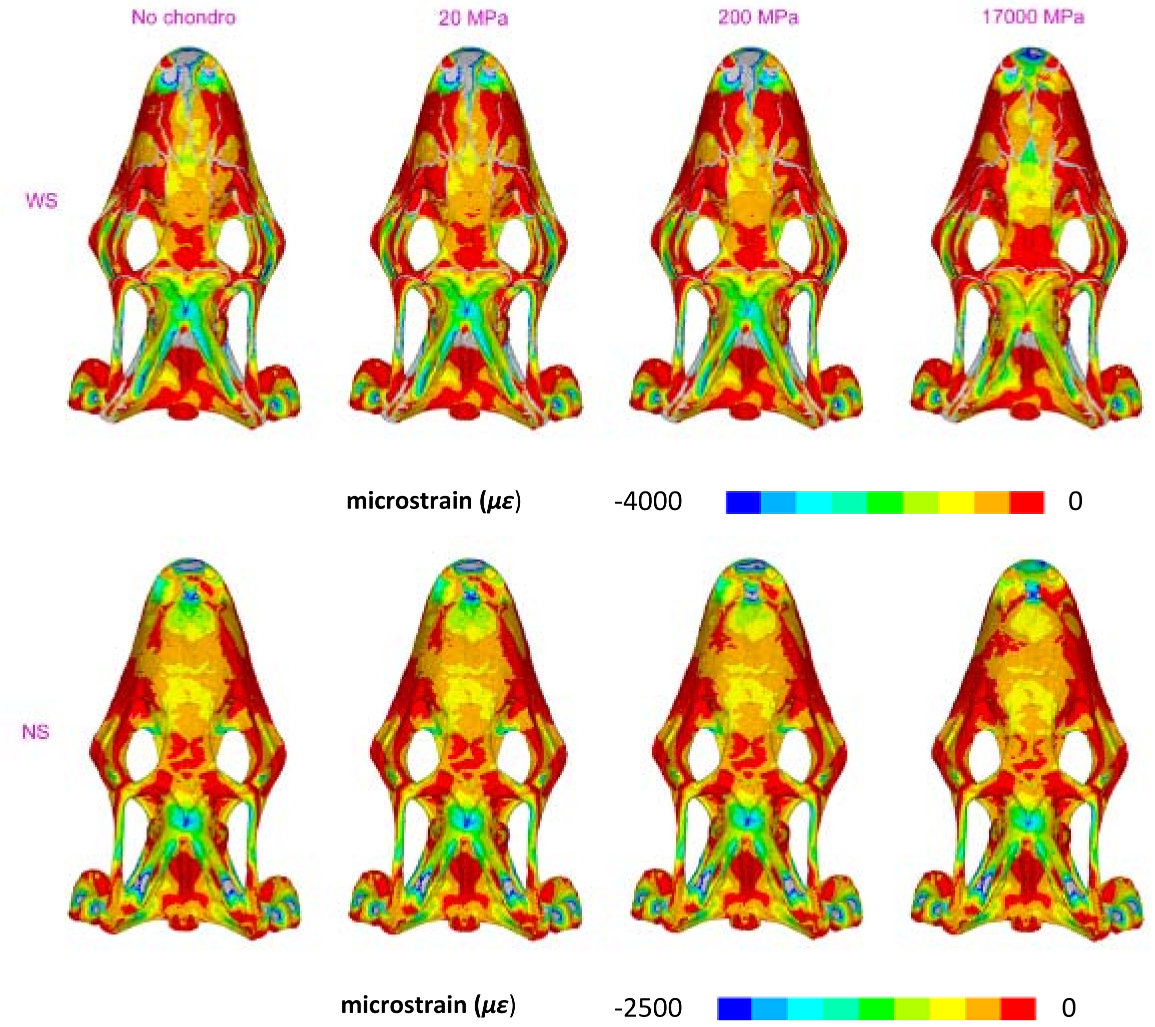

SI Figure 20. Contour plots of 3rd principal strain in the cranium under anterior biting in dorsal view.

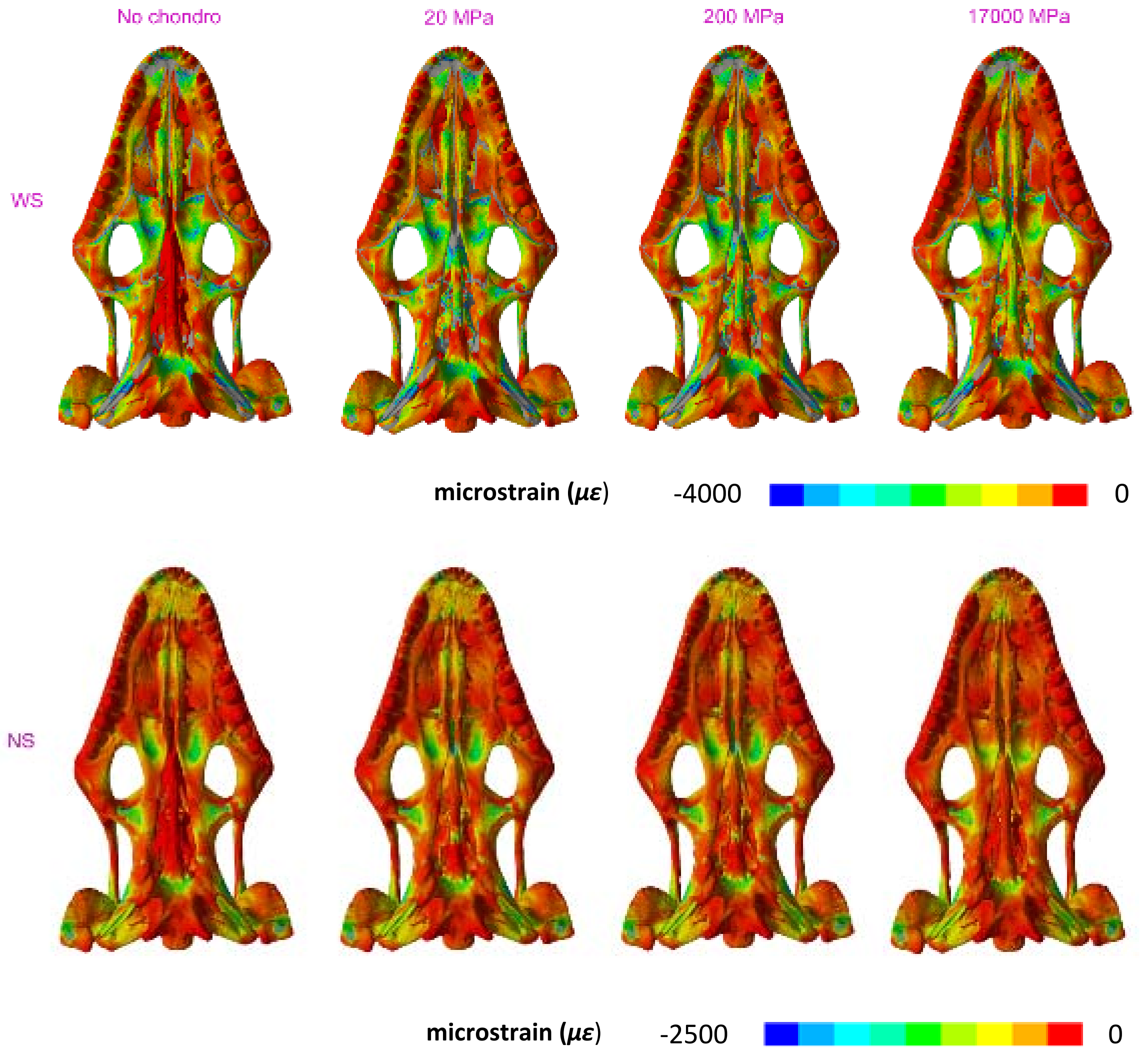

SI Figure 21. Contour plots of 3rd principal strain in the cranium under anterior biting in ventral view.

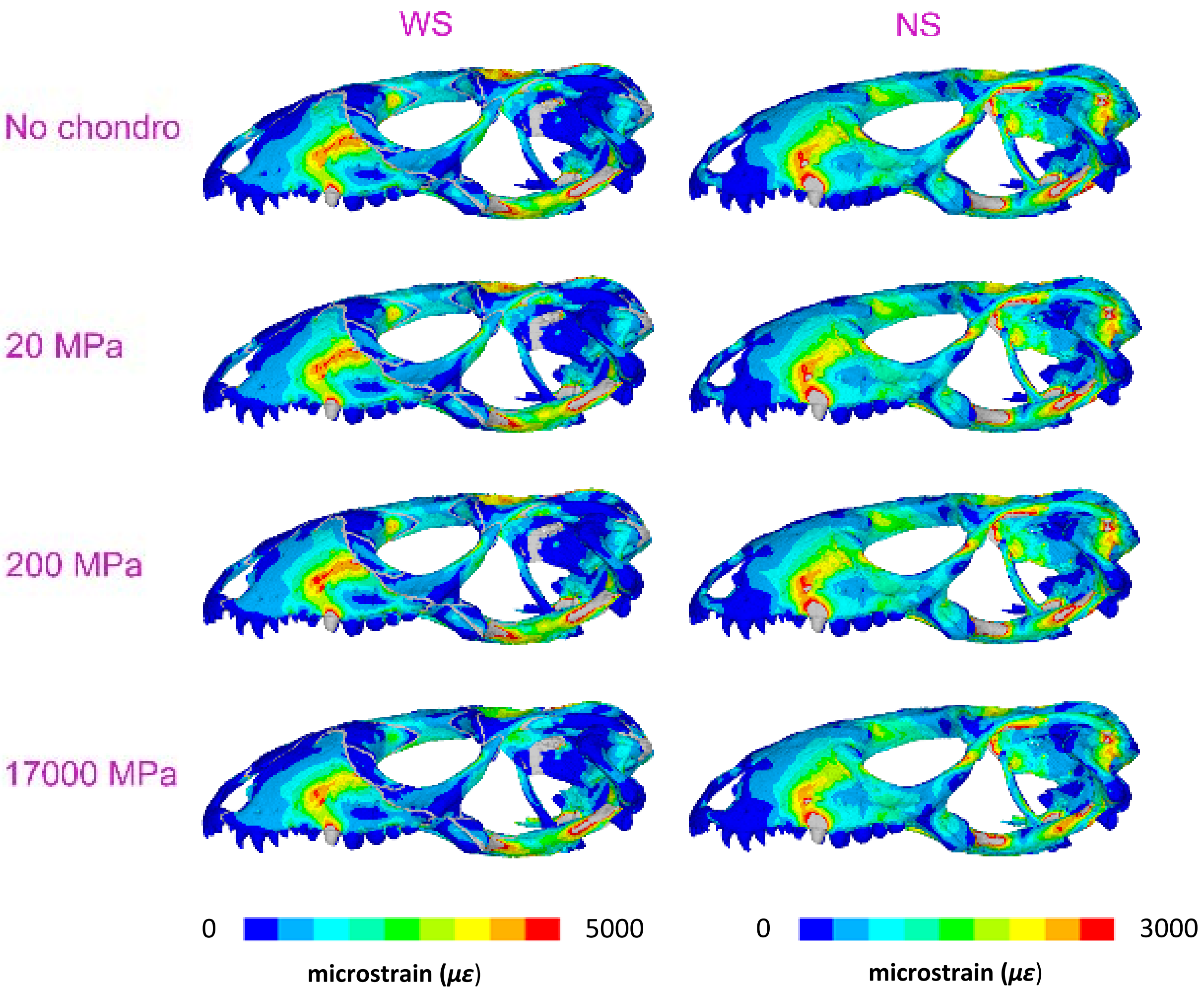

SI Figure 22. Contour plots of von Mises strain in the cranium under posterior (unilateral) left side biting in lateral view.

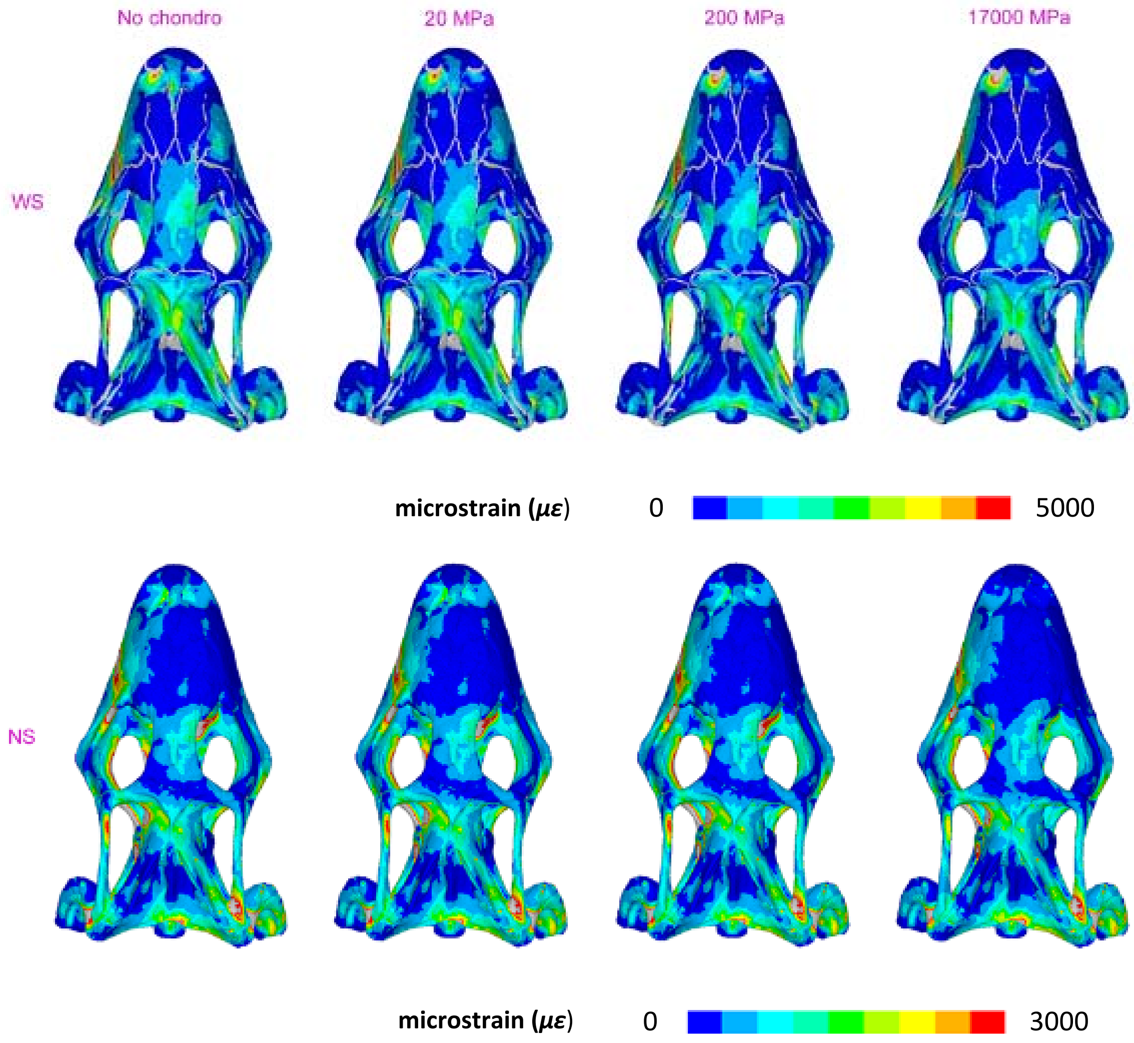

SI Figure 23. Contour plots of von Mises strain in the cranium under posterior (unilateral) biting in dorsal view.

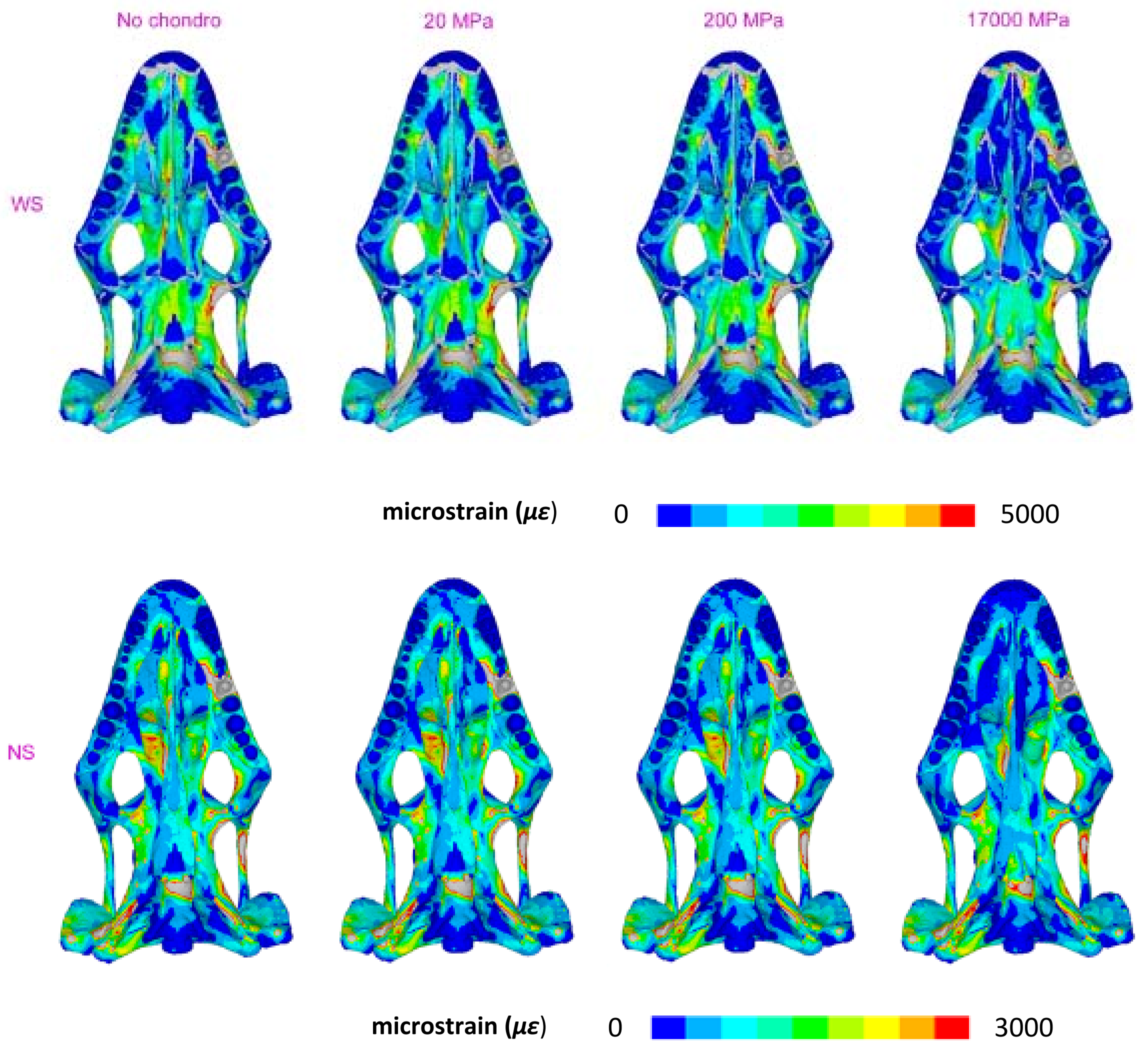

SI Figure 24. Contour plots of von Mises Strain in the cranium under posterior (unilateral) biting in ventral view.

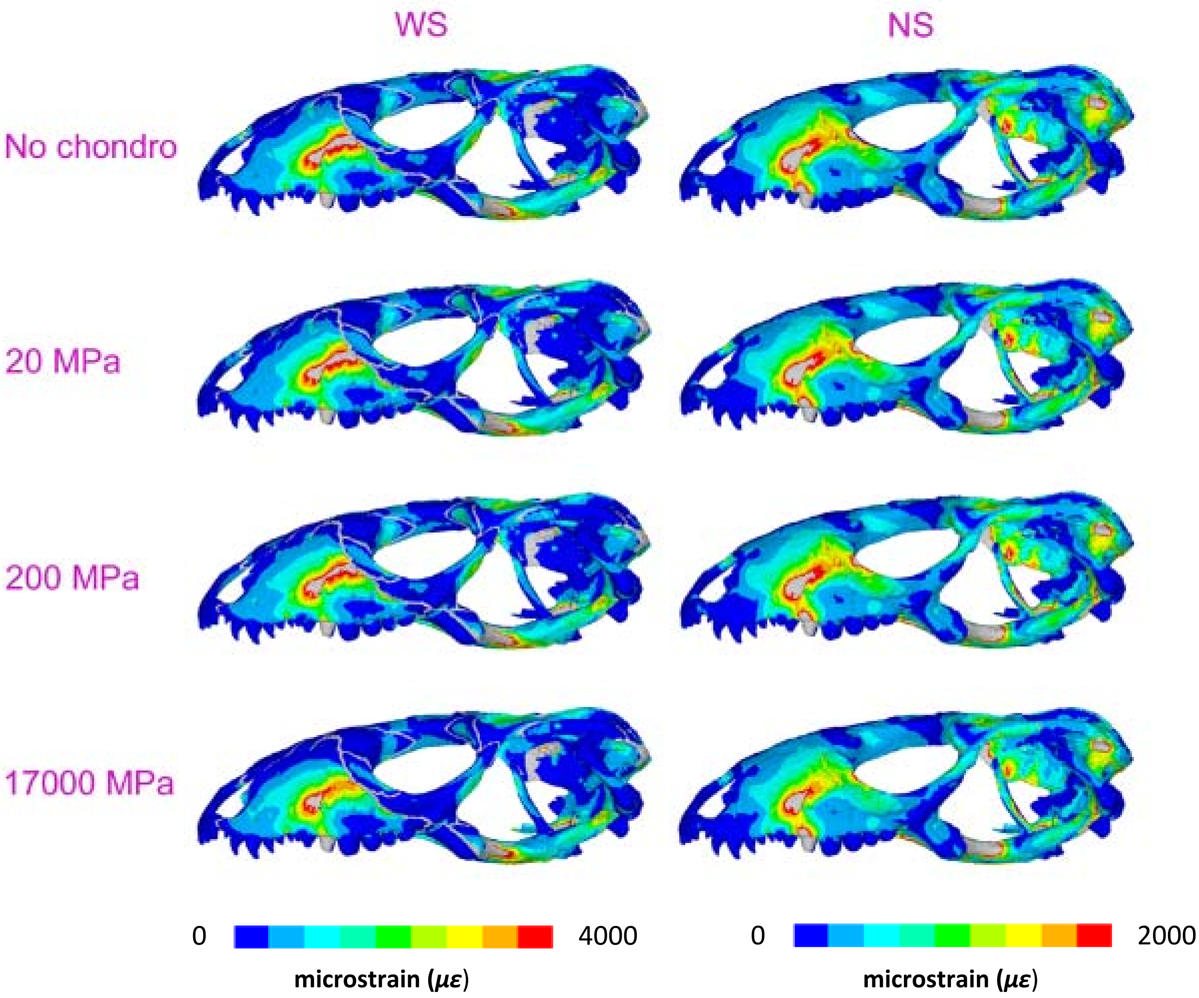

SI Figure 25. Contour plots of 1st principal strain in the cranium under posterior (unilateral) left side biting in lateral view.

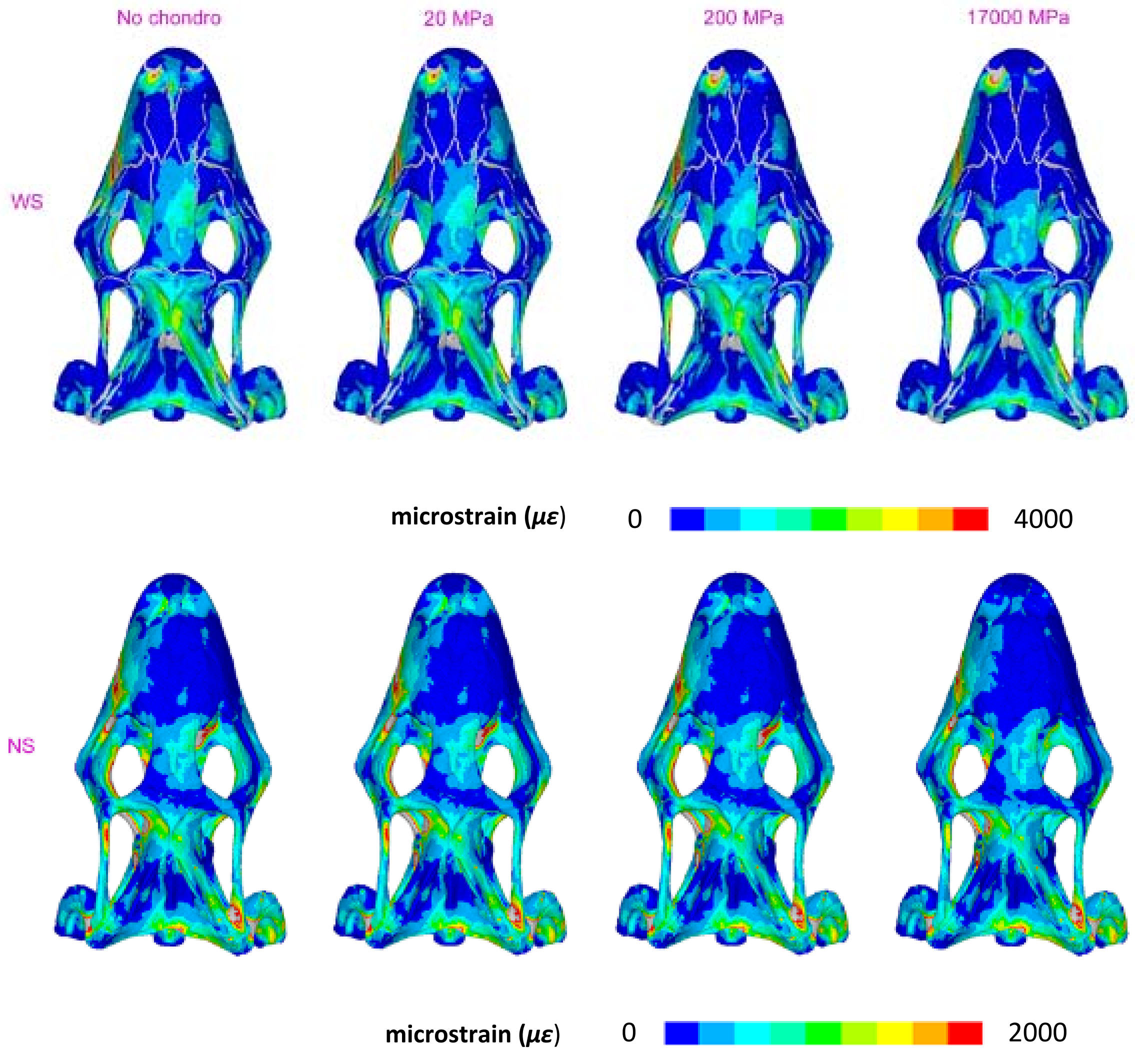

SI Figure 26. Contour plots of 1st principal strain in the cranium under posterior (unilateral) biting in dorsal view.

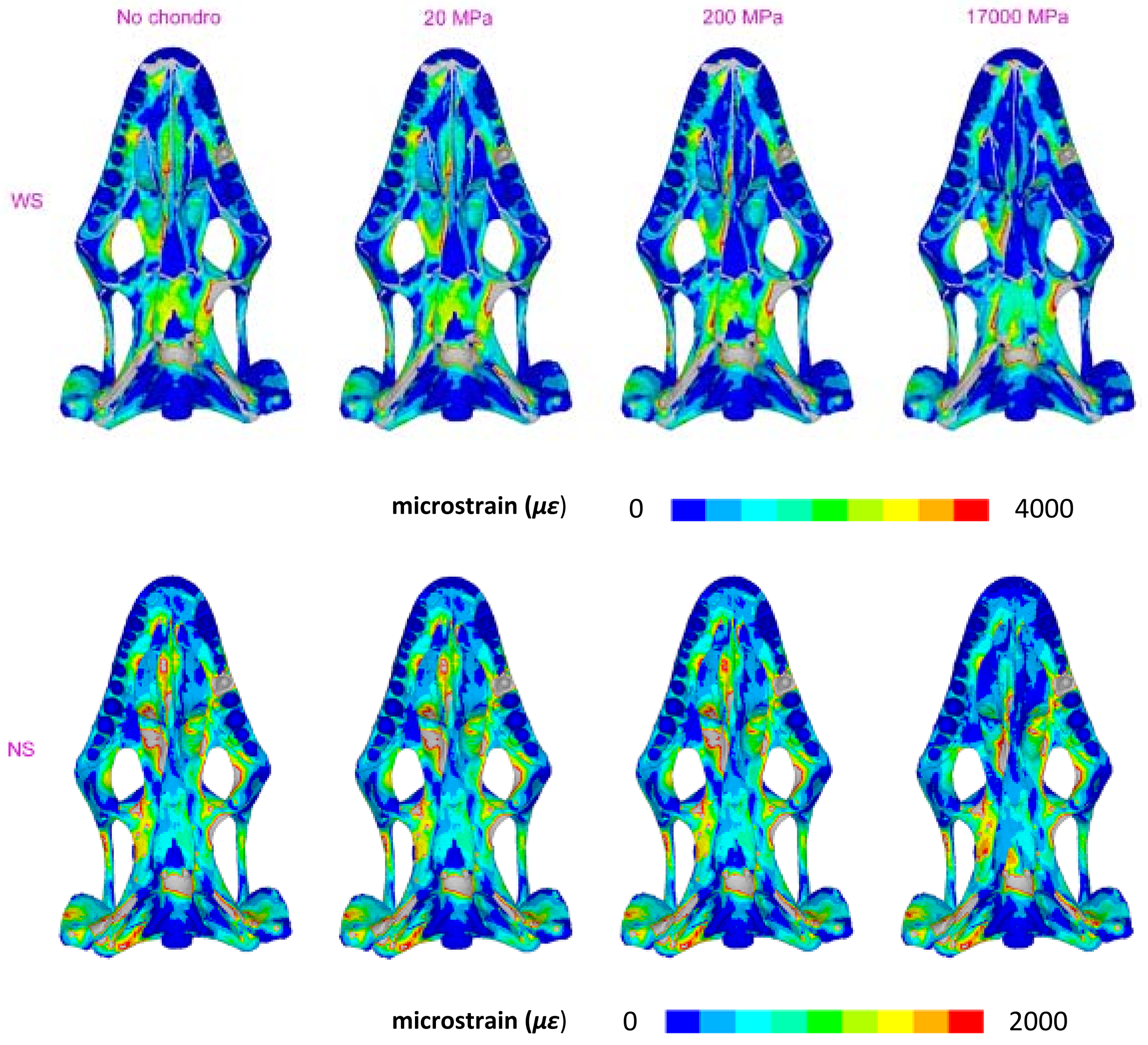

SI Figure 27. Contour plots of 1st principal strain in the cranium under posterior (unilateral) biting in ventral view.

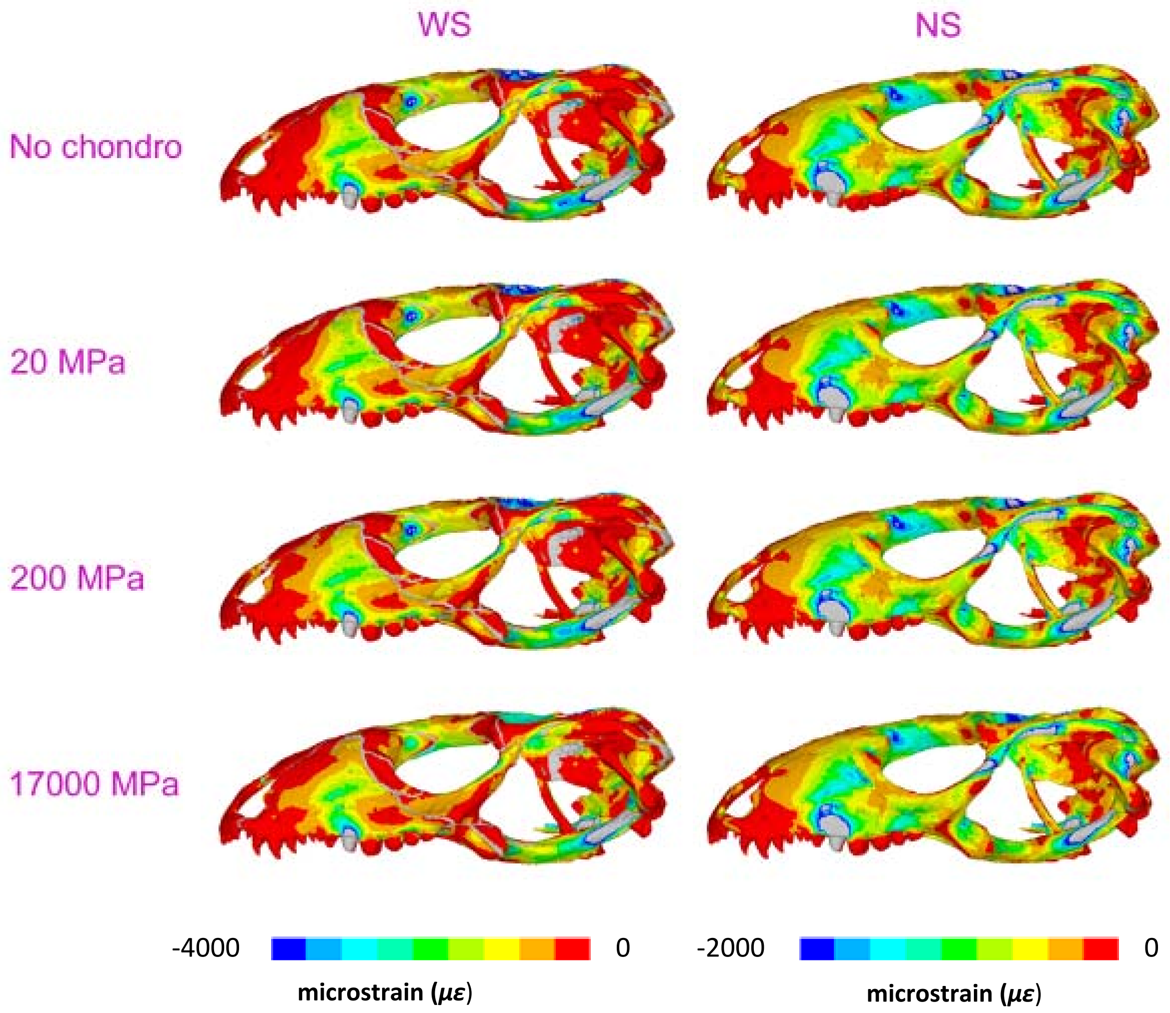

SI Figure 28. Contour plots of 3rd principal strain in the cranium under posterior (unilateral) biting in lateral view.

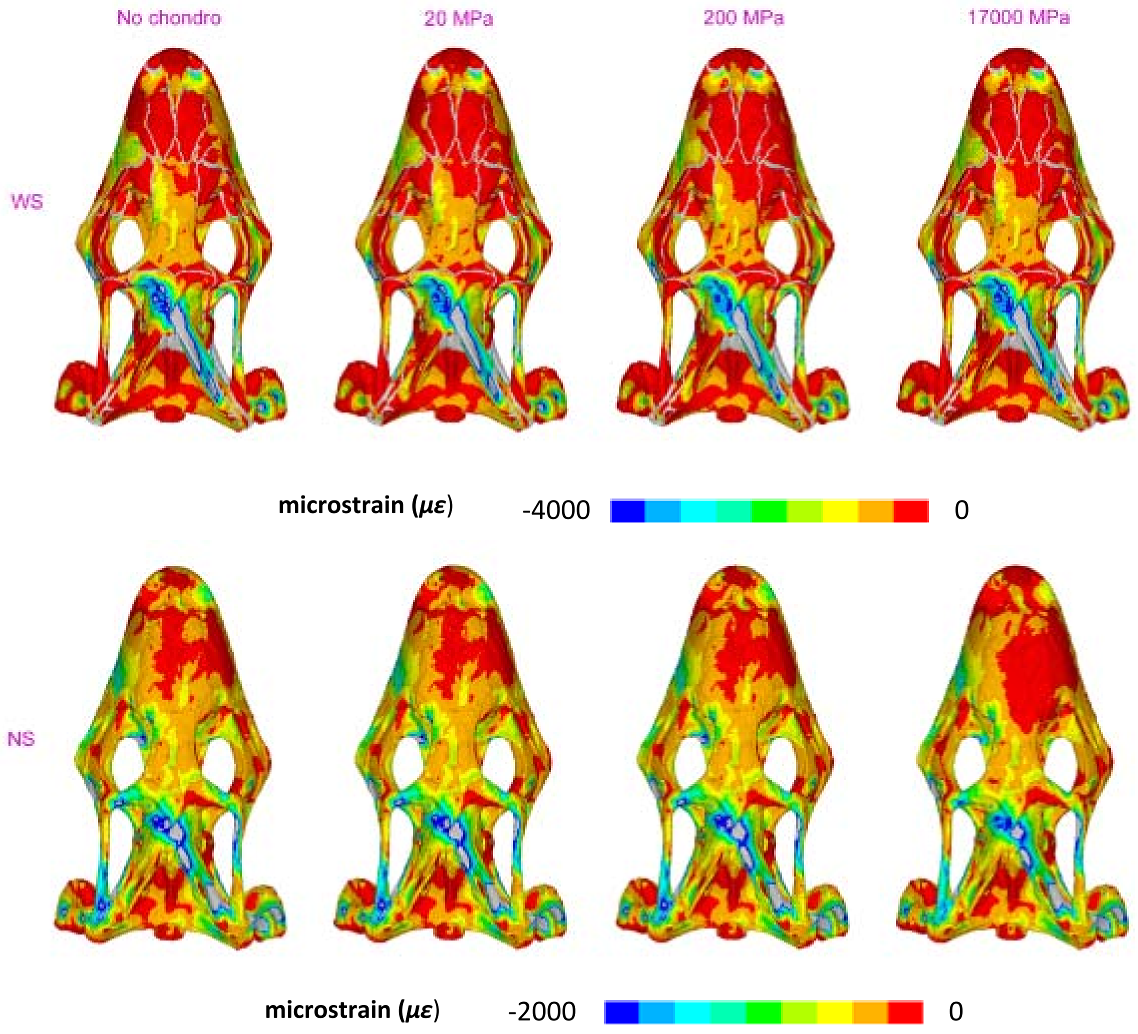

SI Figure 29. Contour plots of 3rd principal strain under posterior (unilateral) biting in dorsal view.

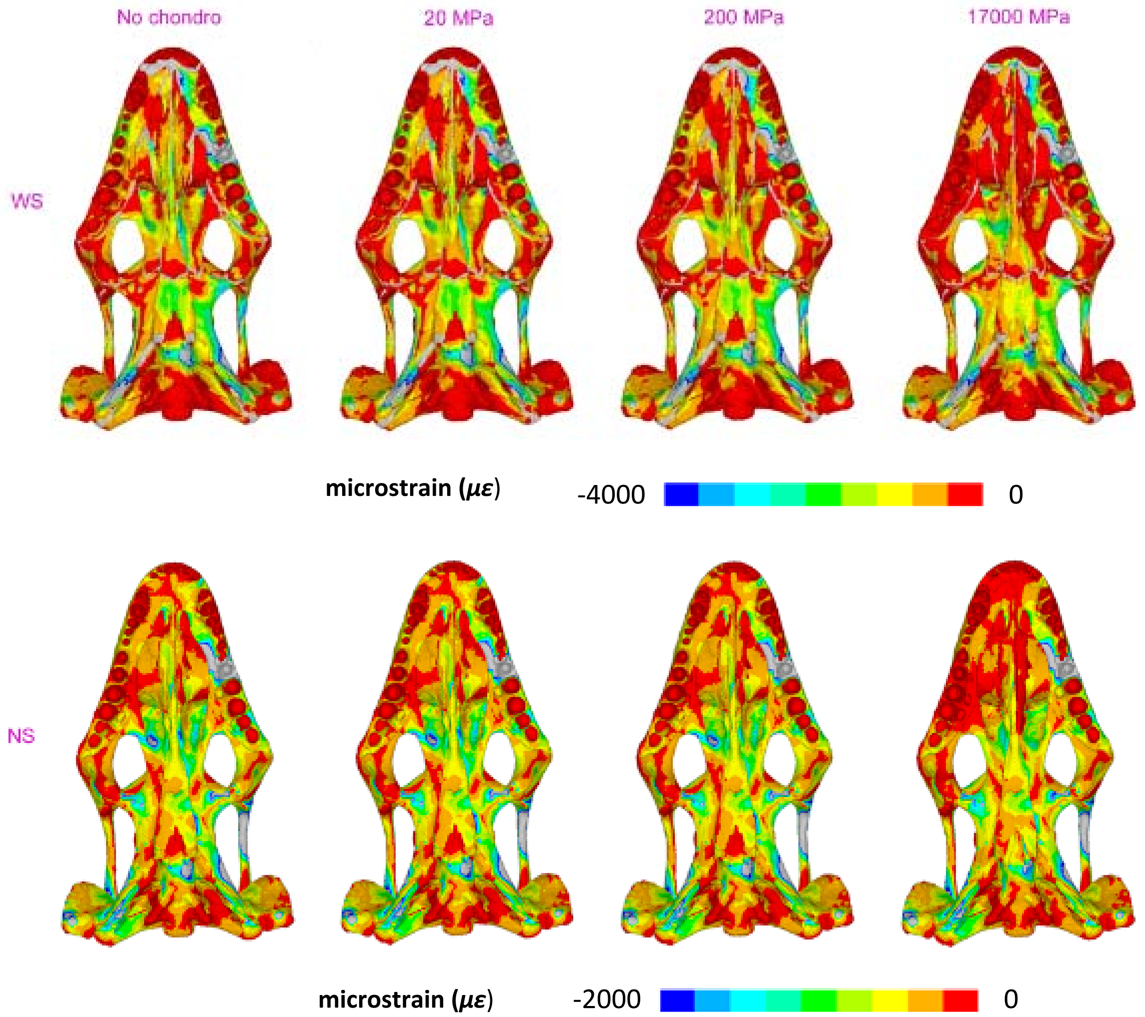

SI Figure 30. Contour plots of 3rd principal strain under posterior (unilateral) biting in ventral view.

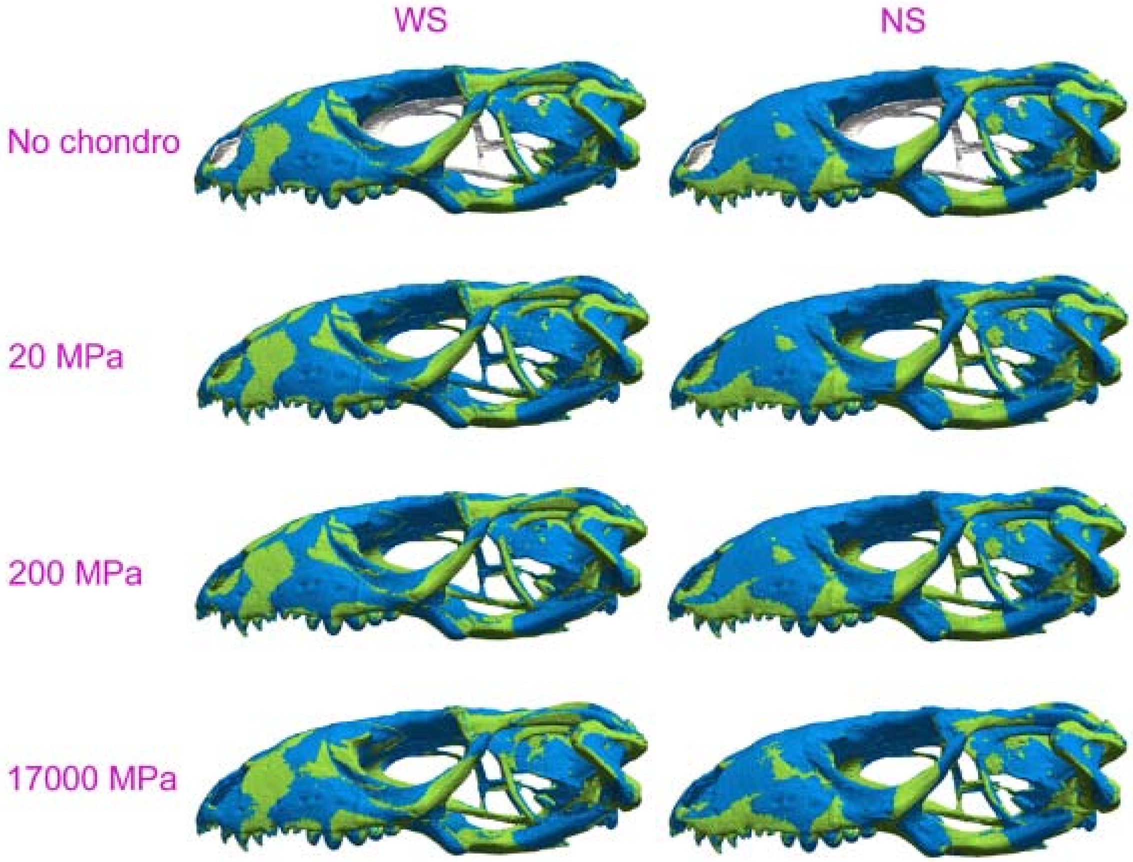

SI Figure 31. Plots showing whether compression (blue) or tension (green) dominates during anterior biting in left lateral view.

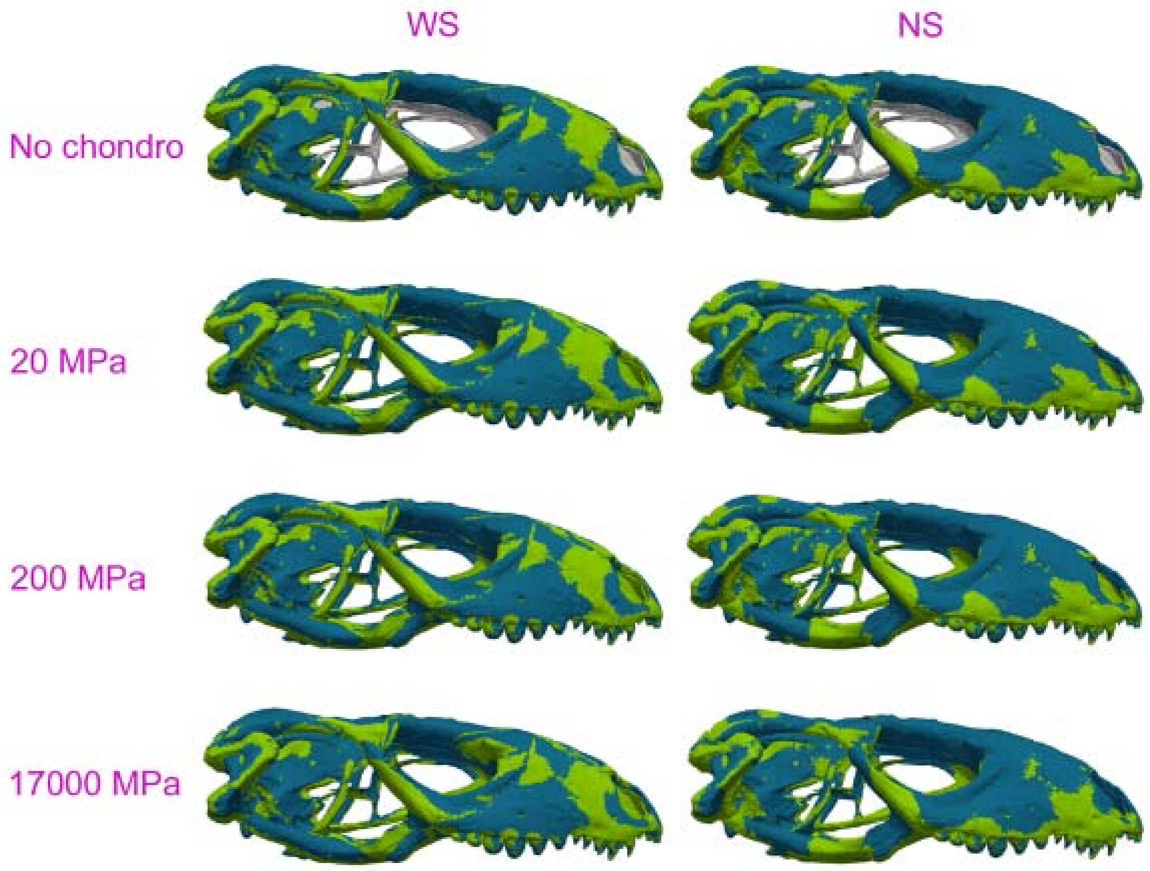

SI Figure 32. Plots showing whether compression (blue) or tension (green) dominates during anterior biting right lateral view.

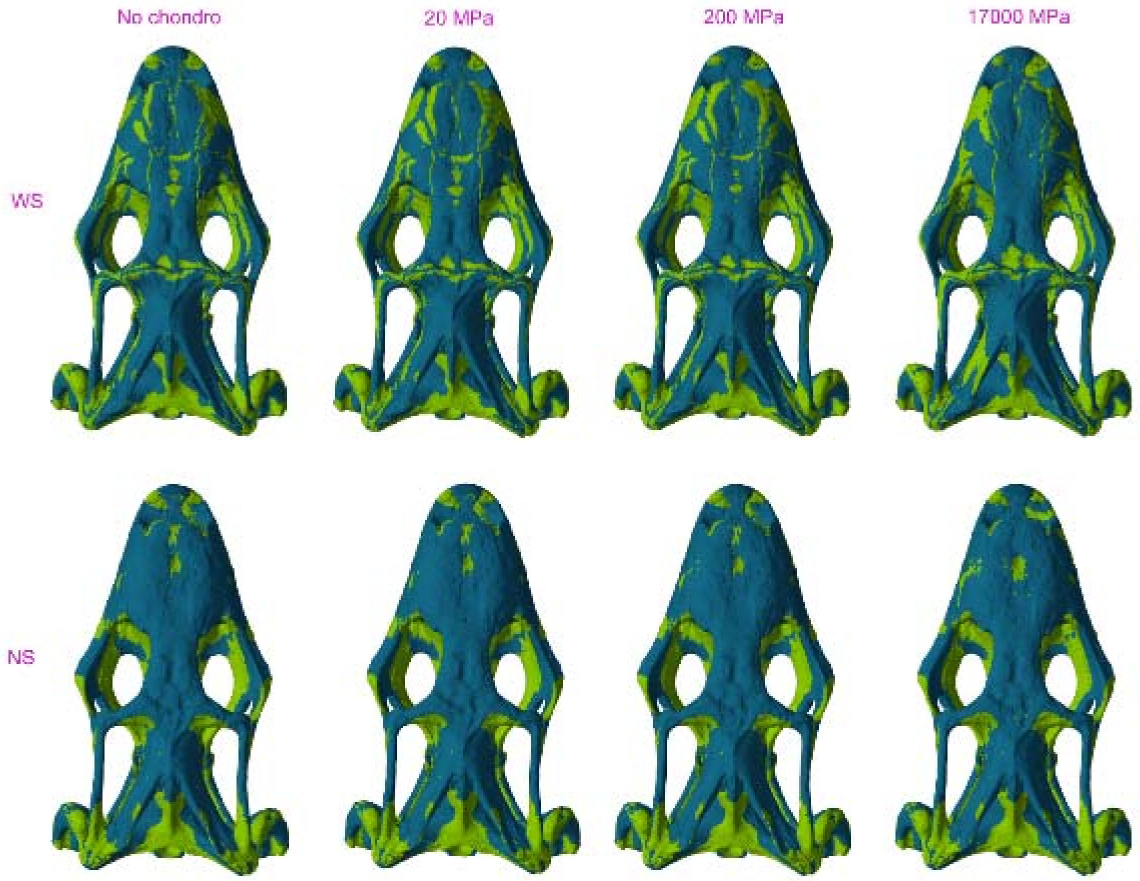

SI Figure 33. Plots showing whether compression (blue) or tension (green) dominates during anterior biting in dorsal view.

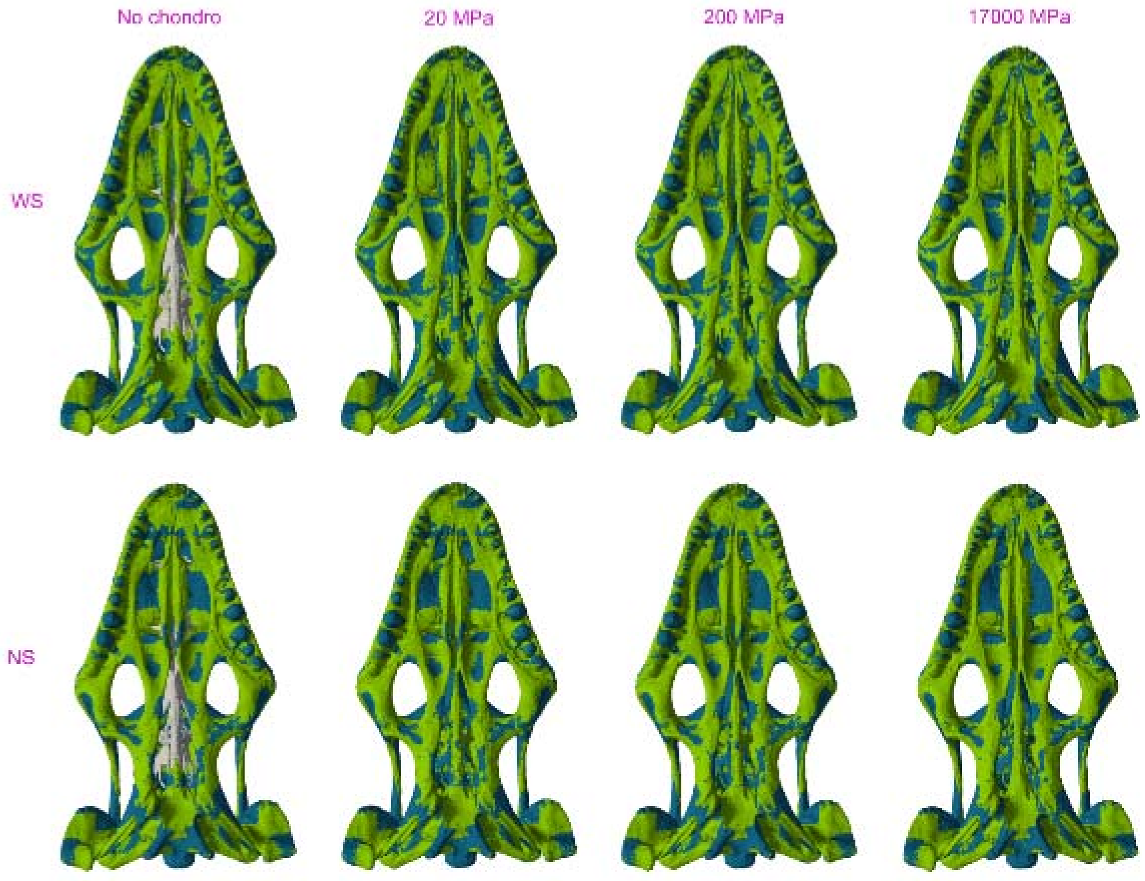

SI Figure 34. Plots showing whether compression (blue) or tension (green) dominates during anterior biting in ventral view.

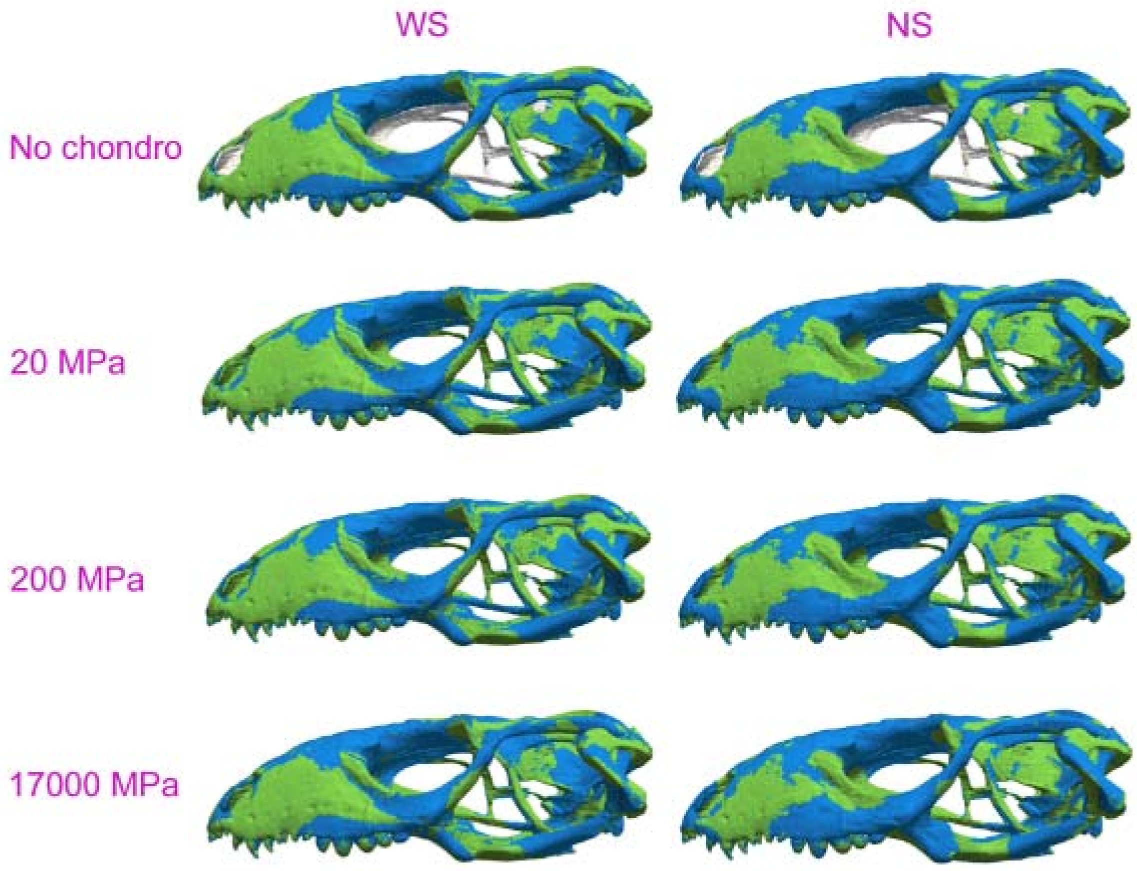

SI Figure 35. Plots showing whether compression (blue) or tension (green) dominates during left side posterior biting in left lateral view.

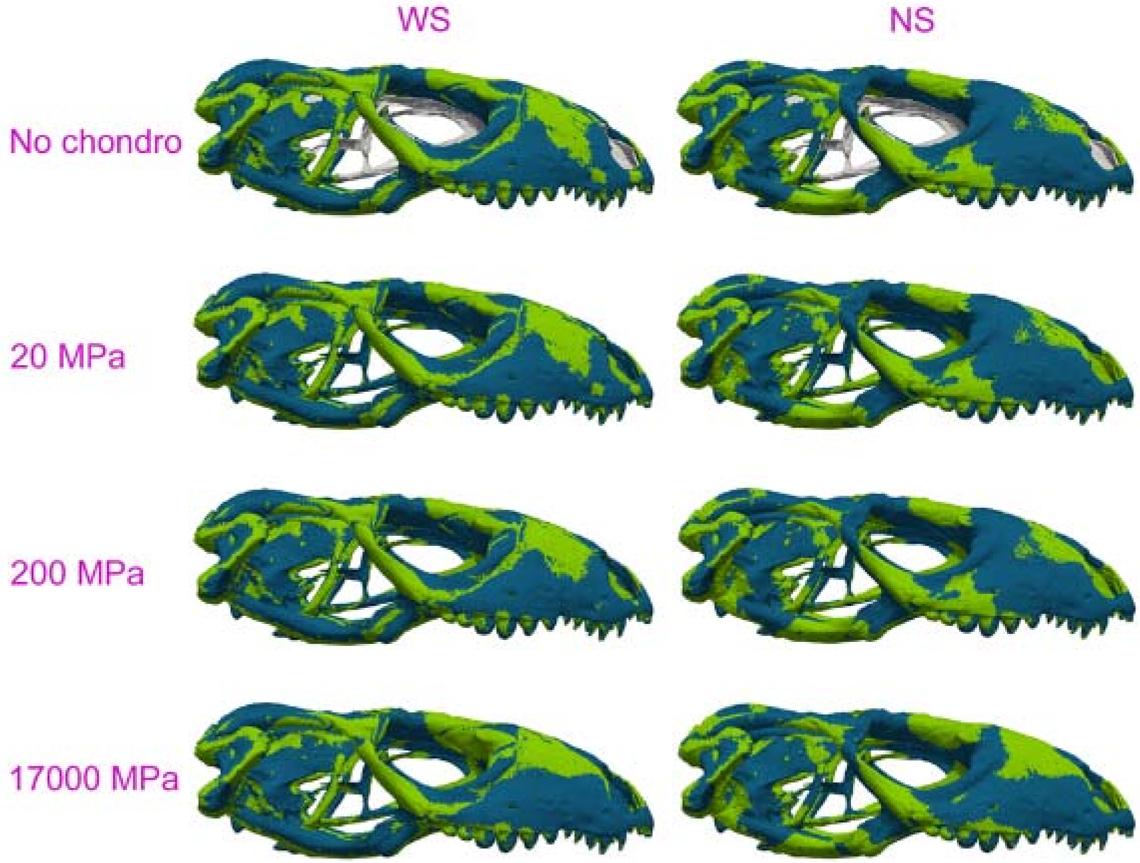

SI Figure 36. Plots showing whether compression (blue) or tension (green) dominates during left side posterior biting in right lateral view.

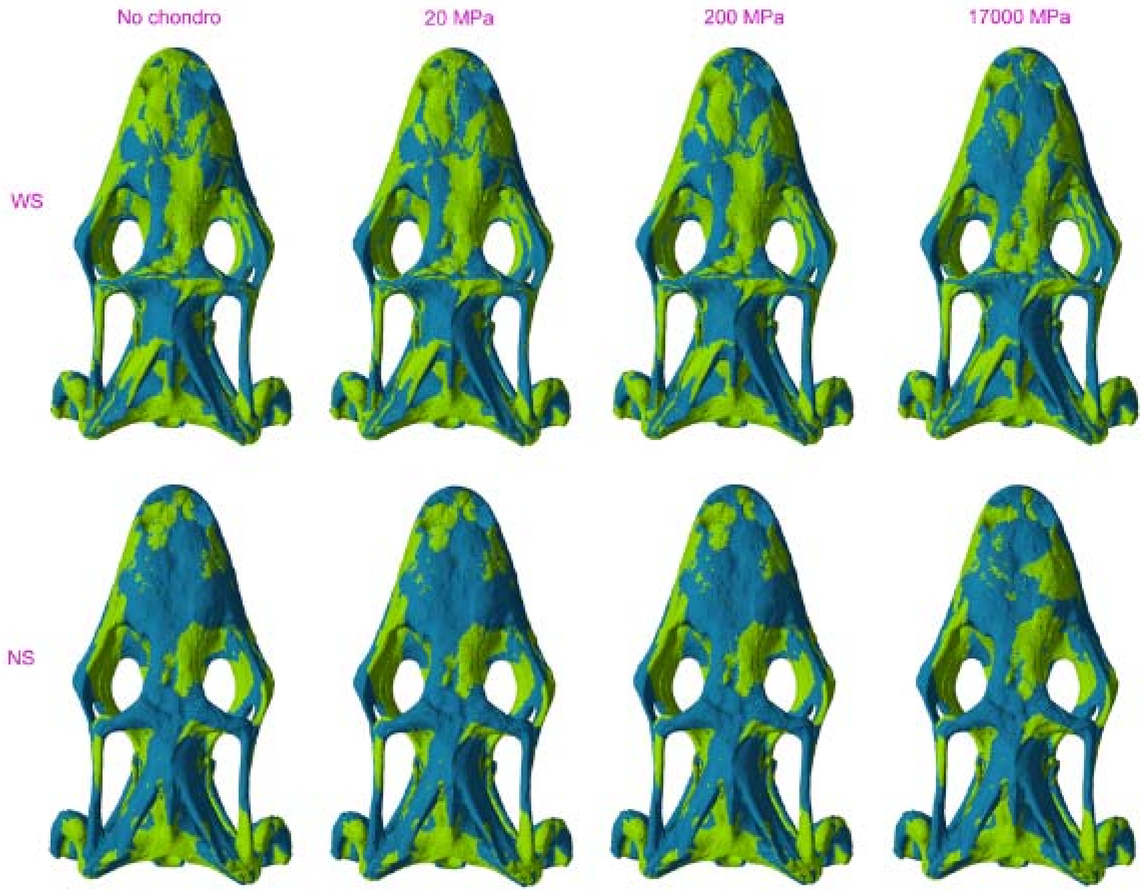

SI Figure 37. Plots showing whether compression (blue) or tension (green) dominates during posterior biting in dorsal view.

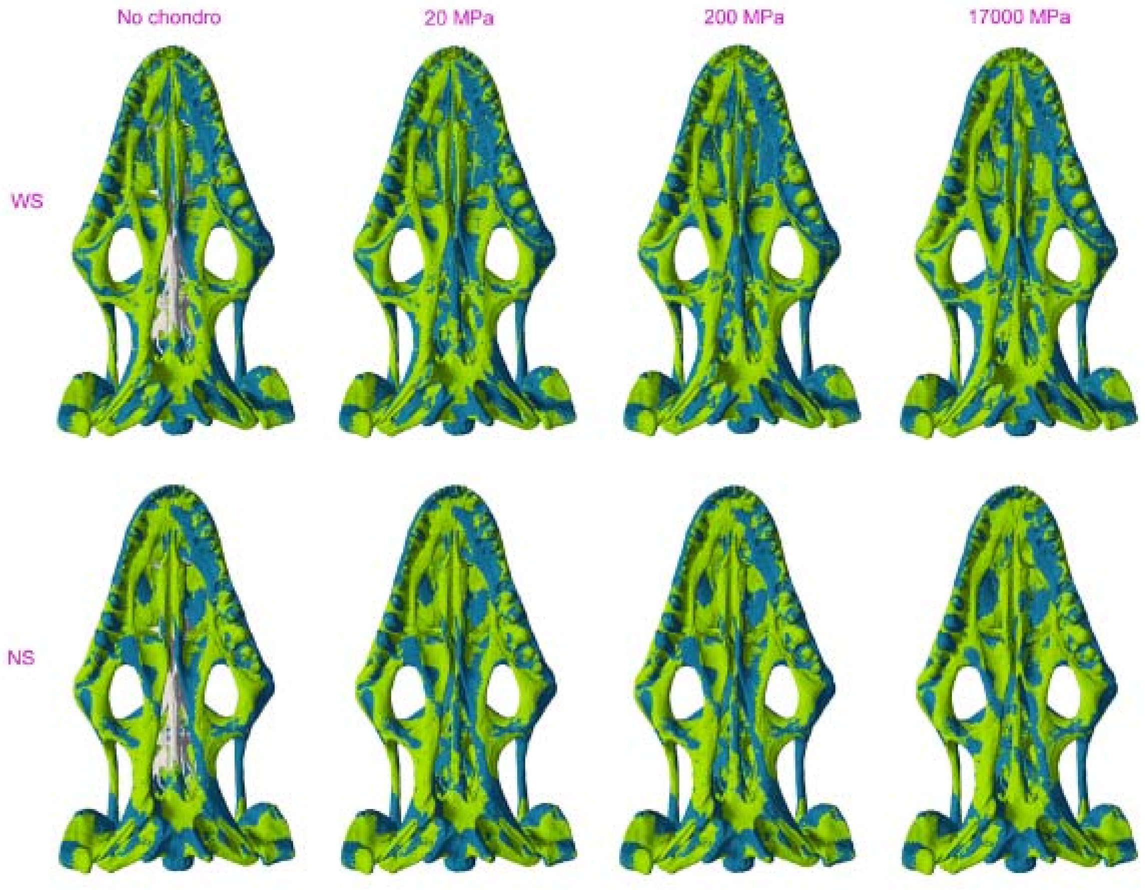

SI Figure 38. Plots showing whether compression (blue) or tension (green) dominates during posterior biting in ventral view.

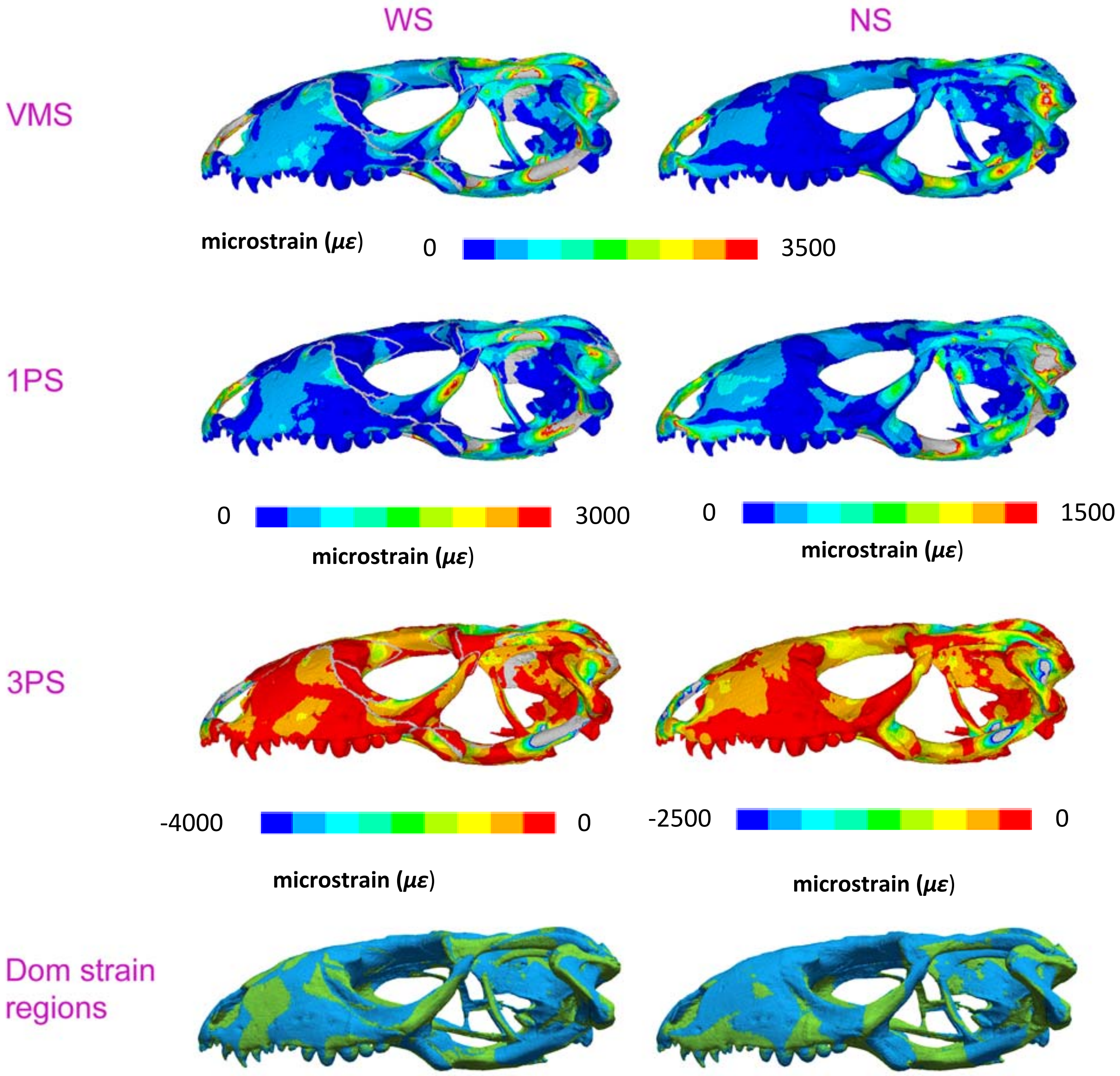

SI Figure 39. Contour strain plots and tension-compression dominance plots for a model (that includes a chondrocranium modelled with material properties of 200 MPa) during anterior biting in lateral view. (VMS) Von Mises strain, (1PS) 1st principal strain, (3PS) 3rd principal strain.

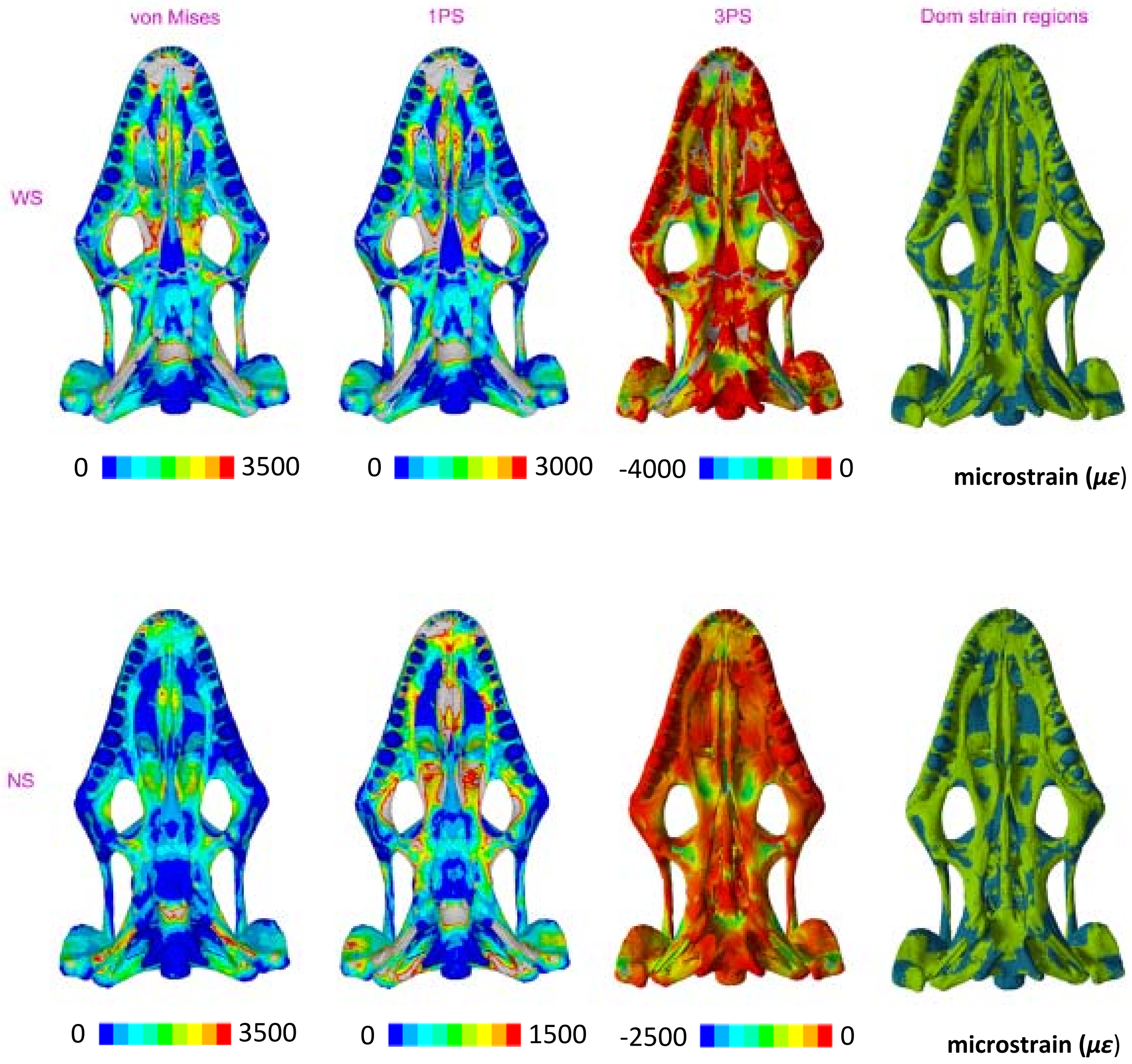

SI Figure 40. Contour strain plots and tension-compression dominance plots for a model (that includes a chondrocranium modelled with material properties of 200 MPa) during anterior biting in ventral view. (VMS) Von Mises strain, (1PS) 1st principal strain, (3PS) 3rd principal strain.

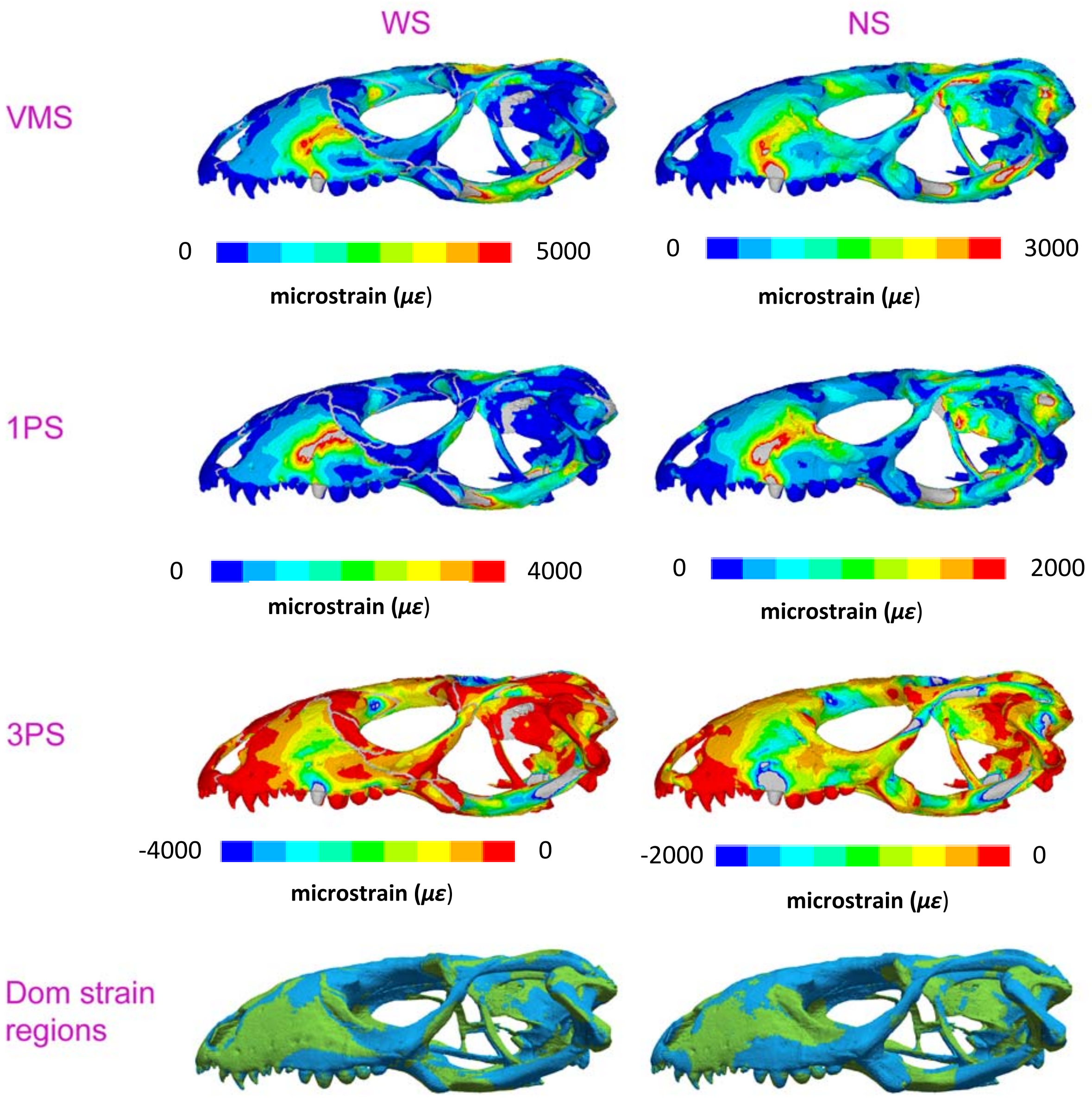

SI Figure 41. Contour strain plots and tension-compression dominance plots for a model (that includes a chondrocranium modelled with material properties of 200 MPa) during posterior biting in lateral view. (VMS) Von Mises strain, (1PS) 1st principal strain, (3PS) 3rd principal strain.

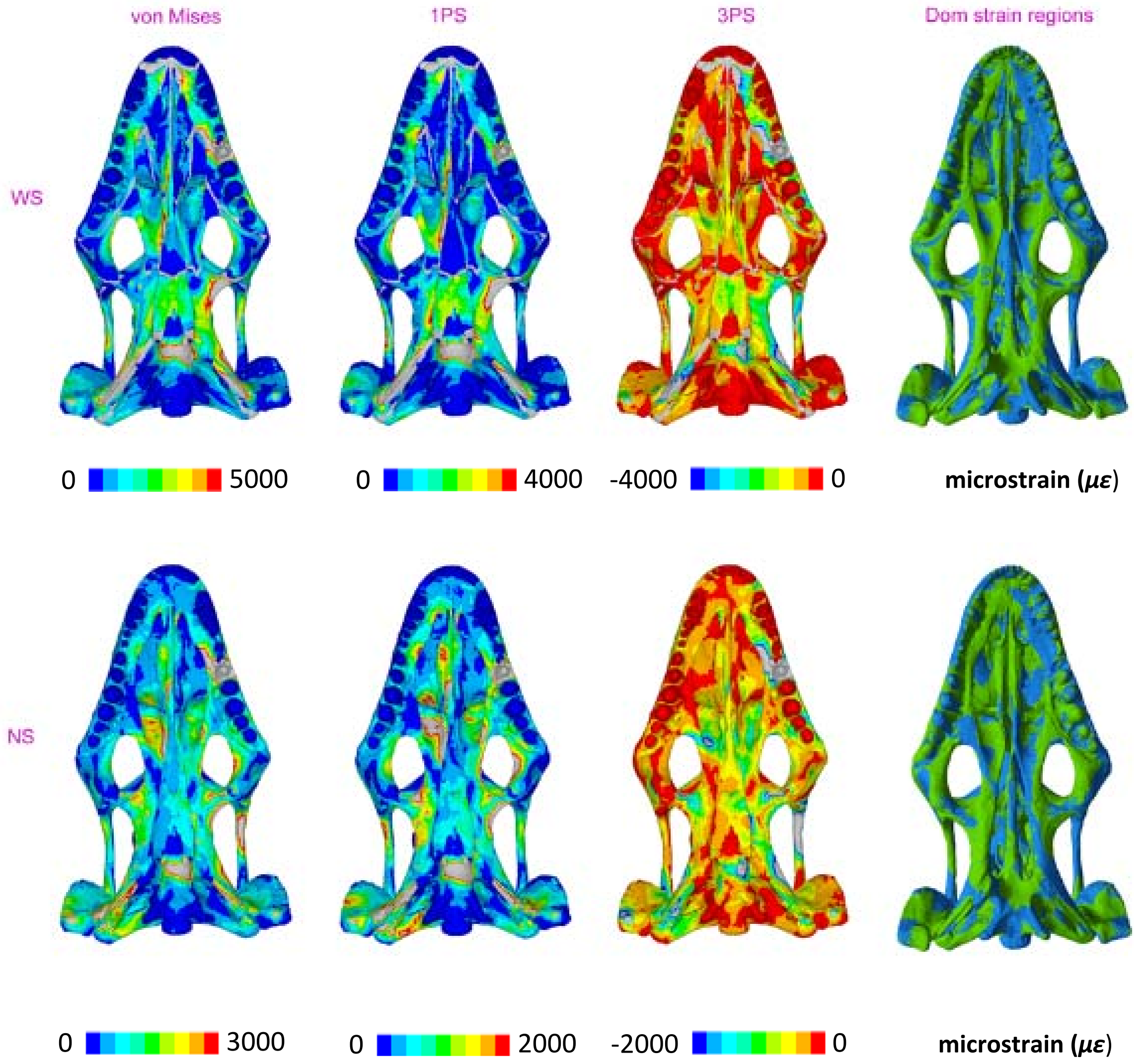

SI Figure 43. Contour strain plots and tension-compression dominance plots for a model (that includes a chondrocranium modelled with material properties of 200MPa) during posterior biting in ventral view. (VMS) Von Mises strain, (1PS) 1st principal strain, (3PS) 3rd principal strain.
