# Supplementary material for: The biomechanical role of the chondrocranium and sutures in a lizard cranium
Source: J R Soc Interface. 2017 Dec 20;14(137):20170637. doi: 10.1098/rsif.2017.0637 (PMC5746569; doi:10.1098/rsif.2017.0637)
Supplement: SI Table 1 [file rsif20170637supp2.pdf]

SI Table 1. Muscle characteristics used in the multi-body dynamics analysis (MDA) and finite element analysis (FEA).

Muscle	Number of strands/side	Weight (g)	Angle of pennation (°)	Mean fascicle length (cm)	Muscle density ($g.cm^{-3}$)	PCSA (cm^2)	Intrinsic strength ($N.cm^{-2}$)	Max. muscle force (N)	Force/strand (N)
		measured	measured	measured	calculated	calculated		calculated	calculated
**mAMEMant**	3	0.7	5.0	1.2	1.0564	0.60	40	24.2	8.1
**mAMEMpost**	9	1.0	0.0	1.1	1.0564	0.98	40	39.1	4.3
**mAMEP3a**	3	0.2	40.0	0.8	1.0564	0.20	40	7.9	2.6
**mAMEP3b**	5	1.1	15.0	0.8	1.0564	1.42	40	56.8	11.4
**mAMEP3c**	3	0.7	10.0	1.0	1.0564	0.70	40	28.0	9.3
**mAMES**	7	3.3	35.0	1.6	1.0564	1.80	40	72.0	10.3
**mAMP**	3	0.5	20.0	0.7	1.0564	0.75	40	30.1	10.0
**mPstP**	4	1.7	2.5	1.8	1.0564	0.98	40	39.0	9.8
**mPstS**	5	1.7	10.0	1.2	1.0564	1.49	40	59.4	11.9
**mPt**	13	14.0	27.5	1.9	1.0564	7.03	40	281.4	21.6
**mDM**	3	0.6	2.5	1.3	1.0564	0.49	40	19.6	6.5
