# Supplementary material for: The biomechanical role of the chondrocranium and sutures in a lizard cranium
Source: J R Soc Interface. 2017 Dec 20;14(137):20170637. doi: 10.1098/rsif.2017.0637 (PMC5746569; doi:10.1098/rsif.2017.0637)
Supplement: SI Table 2 [file rsif20170637supp3.pdf]

SI Table 2. Comparison between bite force estimates from multi-body dynamics, finite element analysis, and *in vivo* measurements.

	Bite Force (N)	Bite Force (N)	Bite Force (N)
Bite point	*In vivo*	ADAMS	ANSYS
Anterior	211	219	215
Posterior (unilateral)	314	299	295

Bite point	Δ ADAMS/*in vivo*	Δ ANSYS/*in vivo*	Δ ANSYS/ADAMS
Anterior	0.04	0.02	0.02
Posterior (unilateral)	0.05	0.06	0.01

In vivo	Measured from the same animal using a Kistler transducer and custom holder
ADAMS	Multibody Dynamics Analysis
ANSYS	Finite Element Analysis
